# Supplementary material for: Cross-species insights into placental evolution and diseases at the single-cell resolution
Source: Nat Commun. 2026 May 9;17:6259. doi: 10.1038/s41467-026-72652-w (PMC13377192; doi:10.1038/s41467-026-72652-w)
Supplement: Supplementary file 1 — Supplementary Information [file 41467_2026_72652_MOESM1_ESM.pdf]

Title

Cross-species insights into placental evolution and diseases at the single-cell resolution

Authors

Guanghui Tan[1, 2†], Ao Zhang[1, 2†], Xuesha Cao[1, 2†], Jingyu Yang[3†], Youjie Cui[1], Fei Wang[1, 2], Tao Shi[1, 2], Hengkuan Li[1, 2], Haoping Wang[1, 2], Huiquan Shan[1, 2], Jilong Ren[1, 2], Yaqi Zhou[1, 2], Menghan Wang[1, 2], Funong Luo[1, 2], Xi Guo[1, 2], Wuqiang Huo[1, 2], Yingran Liu[1, 2], Zhannur Niyazbekova[4], Xihong Wang[1, 2*], Zhenyu Xiao[3*], Yi Zheng[1*], and Yu Jiang[1, 2*]

Supplementary Figs

Supplementary Fig.1: Placental development, cellular composition, and data quality control.
Supplementary Fig.2: Similarity of gene expression in placental cell types across species.
Supplementary Fig.3: Cross-species pseudotime analysis of trophoblast differentiation.
Supplementary Fig.4: Cross-species pseudotime analysis of canine and bovine trophoblast differentiation.
Supplementary Fig.5: Analysis of porcine trophoblast differentiation.
Supplementary Fig.6: Visualization of the alignment of genes grouped together in each cluster along the EVT pathway.
Supplementary Fig.7: Visualization of the alignment of genes grouped together in each cluster along the STB pathway.
Supplementary Fig.8: Changes in gene expression and metabolic activity in the fusion metabolic pathway of trophoblasts.
Supplementary Fig.9: Conservation and divergence of gene expression and KEGG activity in the trophoblast cells.
Supplementary Fig.10: Biased expression of genes in trophoblasts across species.
Supplementary Fig.11: The differentiation activity and expression changes of genes related to pregnancy loss across species.
Supplementary Fig.12: GWAS combined with single-cell transcriptomic analysis revealed the placental cell types associated with recurrent pregnancy loss.
Supplementary Fig.13: The correlation between identified TFs and (recurrent) pregnancy loss.
Supplementary Fig.14: Genome-wide identification of the binding motifs of TGIF1.
Supplementary Fig.15: Uncropped immunoblots for Supplementary Fig.13E.

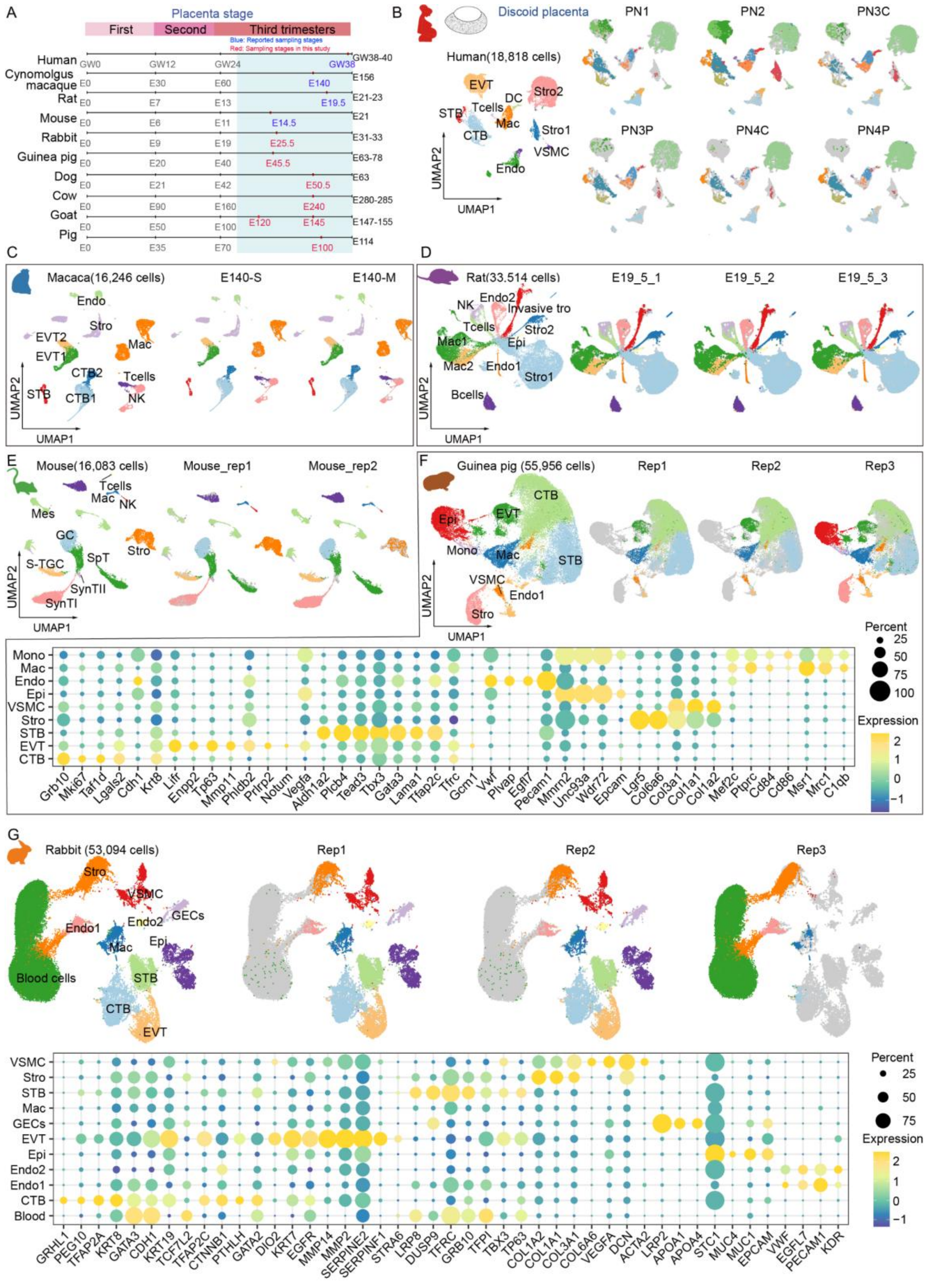

A
Placenta stage
First
Second
Third trimesters
Blue: Reported sampling stages
Red: Sampling stages in this study
Human
GW0
GW12
GW24
GW38
GW38-40
Cynomolgus macaque
E0
E30
E60
E140
E156
Rat
E7
E13
E19.5
E21-23
Mouse
E6
E11
E14.5
E21
Rabbit
E9
E19
E25.5
E31-33
Guinea pig
E20
E40
E45.5
E63-78
Dog
E21
E42
E50.5
E63
Cow
E90
E160
E240
E280-285
Goat
E50
E100
E120
E145
E147-155
Pig
E35
E70
E100
E114
B
Discoid placenta
Human(18,818 cells)
EVT
Stro2
DC
STB
Tcells
Mac
CTB
Stro1
VSMC
Endo
UMAP2
UMAP1
PN1
PN2
PN3C
PN3P
PN4C
PN4P
C
Macaca(16,246 cells)
E140-S
E140-M
Endo
Stro
EVT2
Mac
EVT1
CTB2
Tcells
STB
CTB1
NK
D
Rat(33,514 cells)
E19_5_1
E19_5_2
E19_5_3
NK
Endo2
Invasive tro
Tcells
Stro2
Mac1
Epi
Mac2
Endo1
Stro1
Bcells
E
Mouse(16,083 cells)
Mouse_rep1
Mouse_rep2
Tcells
Mac
NK
Mes
GC
Stro
SpT
S-TGC
SynTII
SynTI
F
Guinea pig (55,956 cells)
Rep1
Rep2
Rep3
CTB
Epi
EVT
Mono
Mac
STB
VSMC
Endo1
Stro
Mono
Mac
Endo
Epi
VSMC
Stro
STB
EVT
CTB
Grb10
Mki67
Taf1d
Lgals2
Cdh1
Krt8
Lifr
Enpp2
Tp63
Mmp11
Phldb2
Prlrp2
Notum
Vegfa
Aldh1a2
Plcb4
Tead3
Tbx3
Gata3
Lama1
Tfap2c
Tfrc
Gcm1
Vwf
Plvap
Egfl7
Pecam1
Mmrn2
Unc93a
Wdr72
Epcam
Lgr5
Col6a6
Col3a1
Col1a1
Col1a2
Mef2c
Ptprc
Cd84
Cd86
Msr1
Mrc1
C1qb
Percent
25
50
75
100
Expression
2
1
0
−1
G
Rabbit (53,094 cells)
Rep1
Rep2
Rep3
Stro
VSMC
GECs
Endo2
Endo1
Epi
Mac
Blood cells
STB
CTB
EVT
VSMC
Stro
STB
Mac
GECs
EVT
Epi
Endo2
Endo1
CTB
Blood
GRHL1
PEG10
TFAP2A
KRT8
GATA3
CDH1
KRT19
TCF7L2
TFAP2C
CTNNB1
PTHLH
GATA2
DIO2
KRT7
EGFR
MMP14
MMP2
SERPINE2
SERPINF1
STRA6
LRP8
DUSP9
TFRC
GRB10
TFPI
TBX3
TP63
COL1A2
COL1A1
COL3A1
COL6A6
VEGFA
DCN
ACTA2
LRP2
APOA1
APOA4
STC1
MUC4
MUC1
EPCAM
VWF
EGFL7
PECAM1
KDR
Percent
25
50
75
Expression
2
1
0
−1

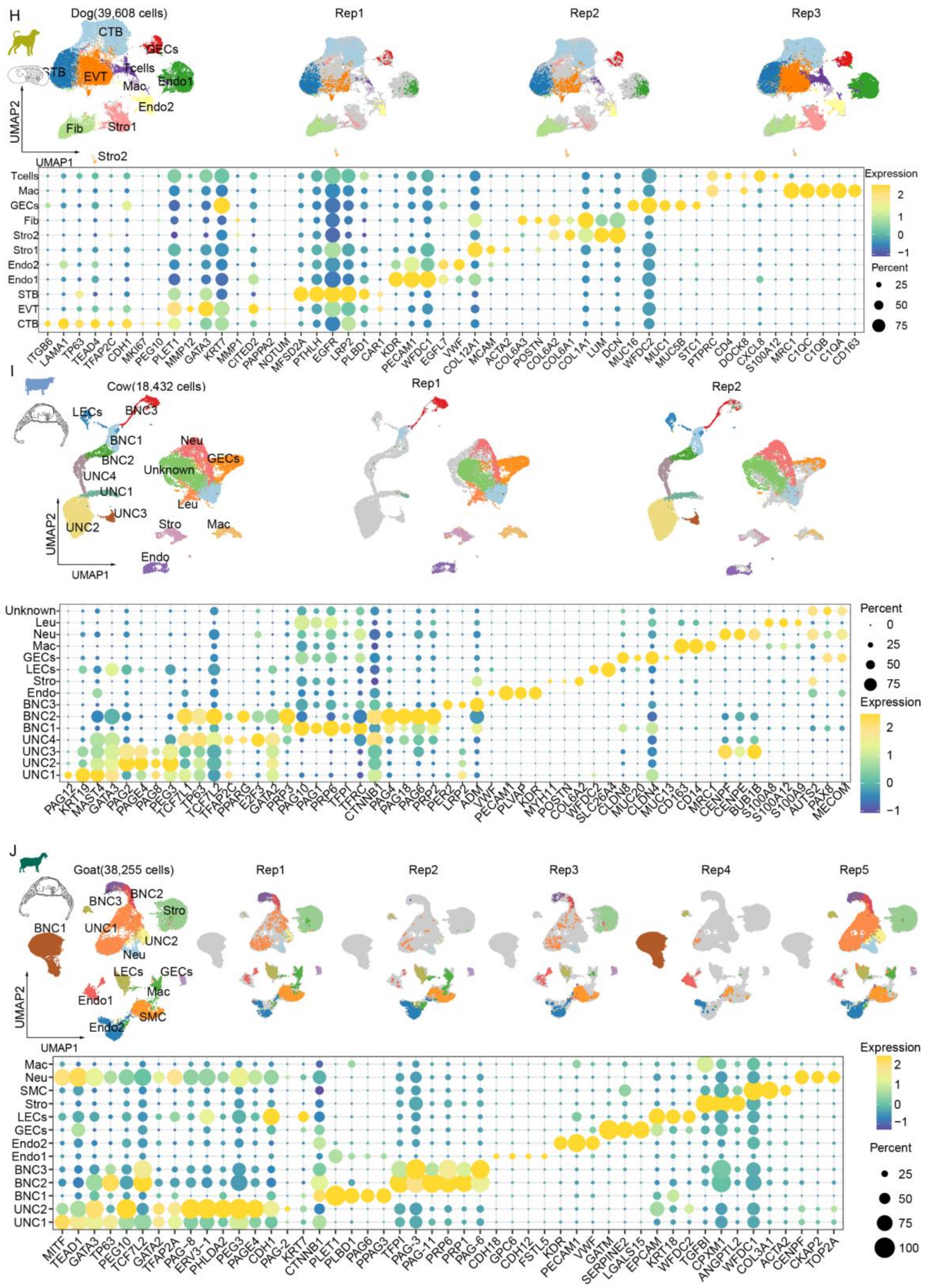

H
Dog(39,608 cells)
Rep1
Rep2
Rep3
CTB
STB
EVT
GECs
Tcells
Endo1
Mac
Endo2
Stro1
Fib
Stro2
UMAP2
UMAP1
Tcells
Mac
GECs
Fib
Stro2
Stro1
Endo2
Endo1
STB
EVT
CTB
Expression
Percent
25
50
75
ITGB6
LAMA1
TP63
TEAD4
TFAP2C
CDH1
MKI67
PEG10
PLET1
MMP12
GATA3
KRT7
MMP1
CITED2
PAPPA2
NOTUM
MFSD2A
PTHLH
EGFR
LRP2
PLBD1
CAR1
KDR
PECAM1
WFDC1
EGFL7
VWF
COL12A1
MCAM
ACTA2
COL6A3
POSTN
COL6A2
COL6A1
COL1A1
LUM
DCN
MUC16
WFDC2
MUC1
MUC5B
STC1
PTPRC
CD4
DOCK8
CXCL8
S100A12
MRC1
C1QC
C1QB
C1QA
CD163
I
Cow(18,432 cells)
Rep1
Rep2
LECs
BNC3
BNC1
BNC2
Neu
GECs
Unknown
UNC4
UNC1
UNC3
Leu
UNC2
Stro
Mac
Endo
UMAP2
UMAP1
Unknown
Leu
Neu
Mac
GECs
LECs
Stro
Endo
BNC3
BNC2
BNC1
UNC4
UNC3
UNC2
UNC1
Percent
0
25
50
75
Expression
2
1
0
−1
PAG12
KRT19
MASTL
GATA3
PAG2
PAGE4
PAG8
PEG3
TCF7L1
TP63
TCF7L2
TFAP2C
PPARG
E2F3
GATA2
PRP3
PAG10
PAG1
PRP6
TFPI
TFRC
CTNNB1
PAG4
PAG18
PAG6
PRP2
PER2
LRP2
ADM
VWF
PECAM1
PLVAP
KDR
MYH11
POSTN
COL6A2
WFDC2
SLC26A4
CLDN8
MUC20
CLDN4
MUC13
CD163
CD14
MRC1
CENPF
CENPE
BUB1B
S100A8
S100A12
S100A9
AUTS2
PAX8
MECOM
J
Goat(38,255 cells)
Rep1
Rep2
Rep3
Rep4
Rep5
BNC2
BNC3
Stro
BNC1
UNC1
UNC2
Neu
LECs
GECs
Endo1
Mac
SMC
Endo2
UMAP2
UMAP1
Mac
Neu
SMC
Stro
LECs
GECs
Endo2
Endo1
BNC3
BNC2
BNC1
UNC2
UNC1
Expression
2
1
0
−1
Percent
25
50
75
100
MITF
TEAD1
GATA3
TP63
PEG10
TCF7L2
GATA2
TFAP2A
PAG-8
ERV3-1
PHLDA2
PEG3
PAGE4
CDH1
PAG-2
KRT7
CTNNB1
PLET1
PLBD1
PAG6
PPAG3
TFPI
PAG-3
PAG-11
PRP6
PRP1
PAG-6
CDH18
GPC6
CDH12
FSTL5
KDR
PECAM1
VWF
GATM
SERPINE2
LGALS15
EPCAM
KRT18
WFDC2
TGFBI
CPXM1
ANGPTL2
WFDC1
COL3A1
ACTA2
CENPF
CKAP2
TOP2A

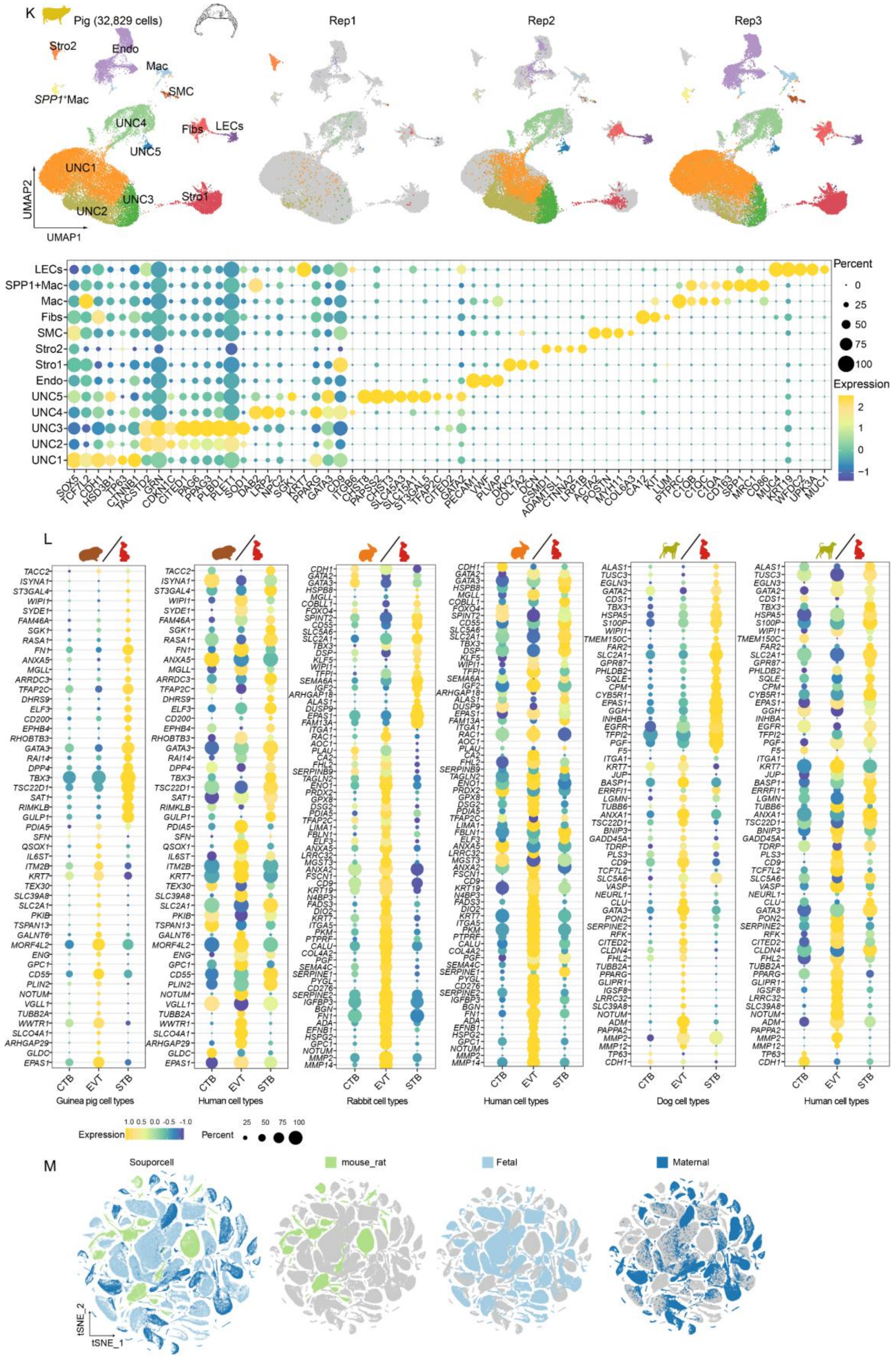

Supplementary Fig.1: Placental development, cellular composition, and data quality control.

(A) Placental development in different mammalian species during pregnancy. Each horizontal line represents a timeline from the beginning (E0 or GW0) to the late pregnancy, with early, middle, and late

stages of placental development marked in different colors. The red markers indicate the sampling points in this and previous studies, including species such as humans, macacas, rats, mice, rabbits, guinea pigs, dogs, cows, goats, and pigs. (B-E) UMAP dimensional reduction visualization of single-cell / -nucleus RNA sequencing (sc- / snRNA-seq) data, where each point represents an individual nucleus. Cell annotations for these species are applied based on labels documented in the literature. In the figure, samples are segregated on the right side, with cells categorized and color-coded by types. (F-K) UMAP dimensional reduction visualization of species-specific placental single-nucleus transcriptomic data, where each point represents an individual nucleus. Cell type annotations were identified based on markers previously reported. The first UMAP for each species displays an integrated map of all biological samples, followed by separate maps for each sample, with cell types distinguished by different colors. (L) The expression of partially conserved genes in EVTs and STB based on orthologous gene conversion in two species. (M) The t-SNE plot showing the fetal or maternal origin of the filtered single cells by the SNP analysis. Notably, the maternal-fetal distinction in mice and rats has not been reported.

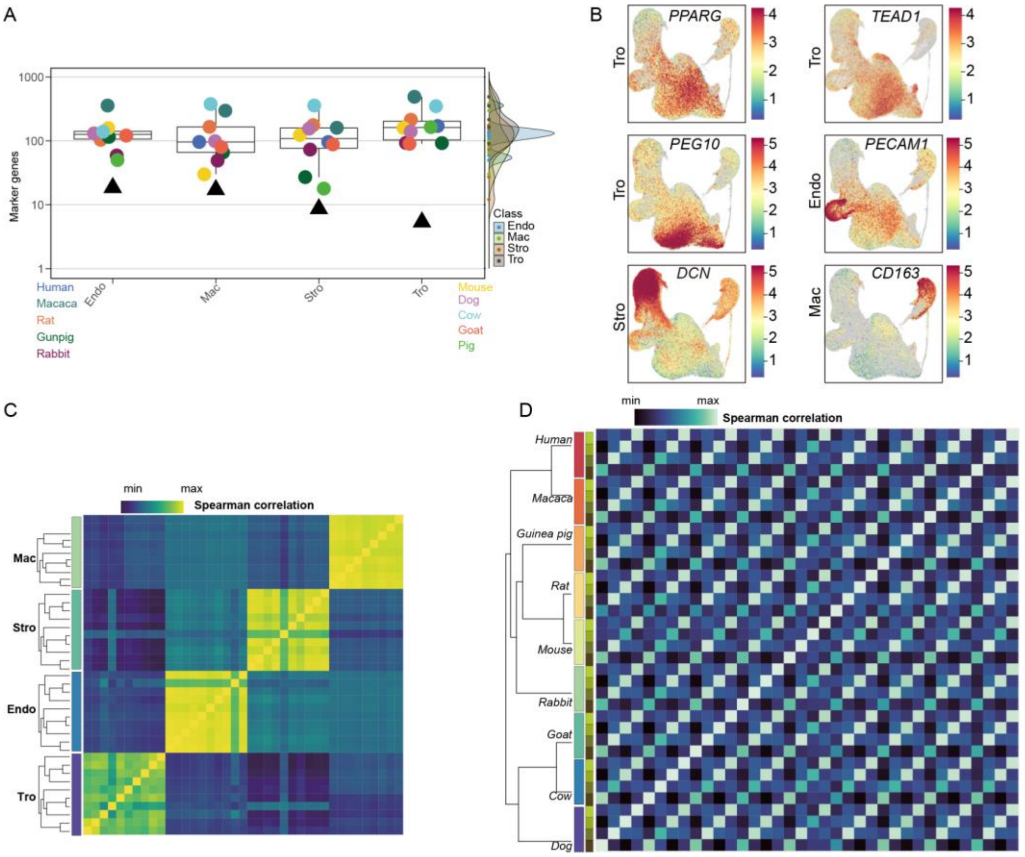

Supplementary Fig.2: Similarity of gene expression in placental cell types across species.

(A) The box-and-line plot showing the number of markers for each cell type in different species. Each point represents the number of markers for a particular species in the corresponding cell type, and the colors distinguish different species. Black triangles represent the number of conserved markers. The density plot on the right side shows the overall number of markers. (B) The UMAP plot showing the expression of six genes in a cell population. The colors indicate the intensity of gene expression. (C) A heatmap showing Spearman correlation of markers (columns) in different cell types (rows). The rows are grouped by cell type and the colors indicate the level of gene expression correlation. The correlation-based clustering of cell types is shown on the left side, revealing the similarity structure among different cell types, and the order of the clustering tree is consistent with that in Fig.2B. (D) A heatmap showing Spearman correlation

of markers in ten mammalian species. Species are grouped by gene expression correlation cluster, and colors indicate the degree of gene expression correlation between species. The clustering tree on the left demonstrates the similarity among the different species, and the species are ordered in a way consistent with the phylogenetic relationships.

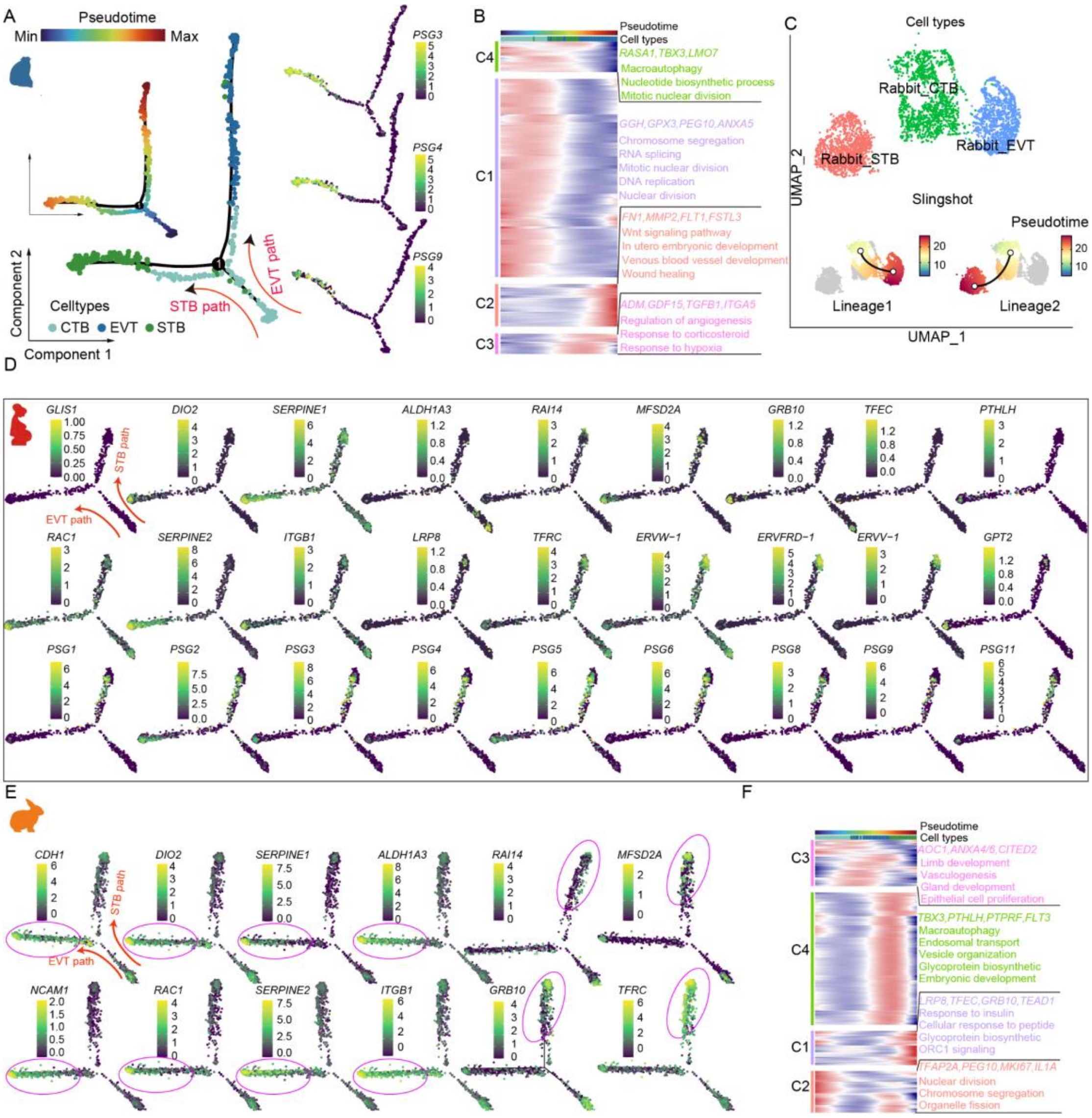

Supplementary Fig.3: Cross-species pseudotime analysis of trophoblast differentiation.

(A) A bivariate scatter plot illustrating the developmental trajectory of trophoblasts in macacas that differentiates into two major pathways: EVTs and STB. (B) A heatmap illustrating the dynamic changes in gene expression related to key biological processes during cell differentiation (Supplementary Data 4). (C) The differentiation trajectory of rabbit placental trophoblasts reconstructed with Slingshot. (D and E) Visualization of specific gene expression patterns along the human (D) and rabbit (E) trophoblast differentiation pathways. (F) A heatmap illustrating the dynamic changes in gene expression related to key biological processes during cell differentiation (Supplementary Data 4).

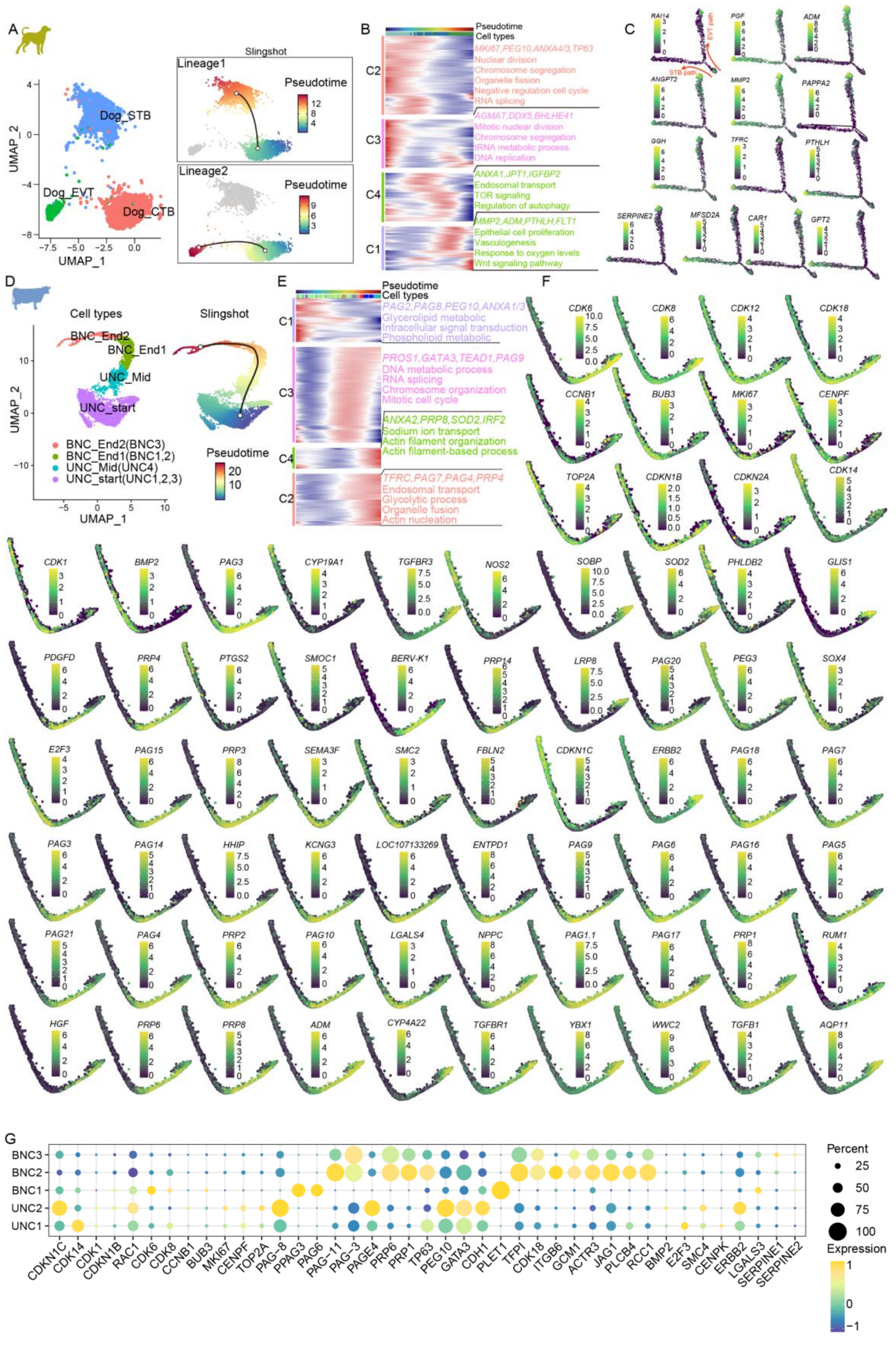

Supplementary Fig.4: Cross-species pseudotime analysis of canine and bovine trophoblast differentiation.

(A) The differentiation trajectory of canine placental trophoblasts reconstructed with Slingshot. (B) A

heatmap illustrating the dynamic changes in gene expression related to key biological processes during cell differentiation (Supplementary Data 4). (C) Visualization of specific gene expression patterns along the canine trophoblast differentiation pathway. (D) The differentiation trajectory of bovine placental trophoblasts reconstructed with Slingshot. (E) A heatmap illustrating the dynamic changes in gene expression related to key biological processes during cell differentiation (Supplementary Data 4). (F) Visualization of specific gene expression patterns along the differentiation pathway of bovine trophoblasts. (G) Expression patterns of differentiation-related genes in caprine trophoblasts.

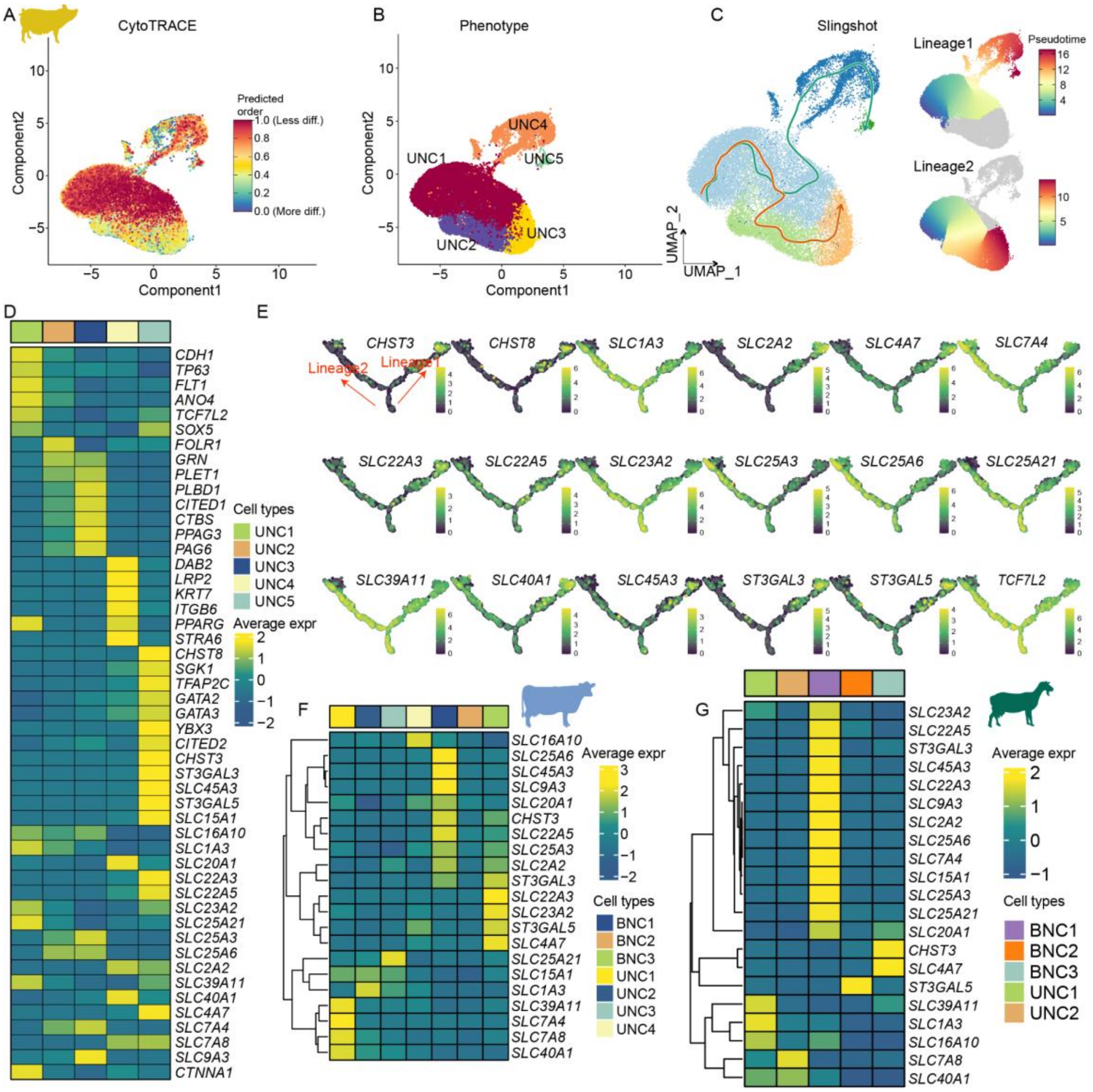

Supplementary Fig.5: Analysis of porcine trophoblast differentiation.

(A) The CytoTRACE plot showing the differentiation status of cell types, with colors ranging from red (more undifferentiated) to blue (more differentiating) representing the degree of differentiation. (B) The UMAP plot displaying the distribution of different cell types (UNC1, UNC2, UNC3, and UNC5) in the porcine placenta. (C) The Slingshot analysis was employed to map the cell lineages, and the colors in the UMAP plot represent the pseudotime progression along the differentiation trajectory. The bottom panels display pseudotime cell distributions along two differentiation pathways (lineages 1 and 2). (D) Specifically expressed genes in each trophoblast subtype in pigs. (E) The expression of genes related to nutrition, metabolic regulation, and maternal fetal material exchanges during the differentiation of porcine trophoblasts. (F and G) Corresponding expression of maternal fetal material exchange-related genes that

were highly expressed in porcine trophoblasts in bovine (F) and caprine (G) trophoblasts.

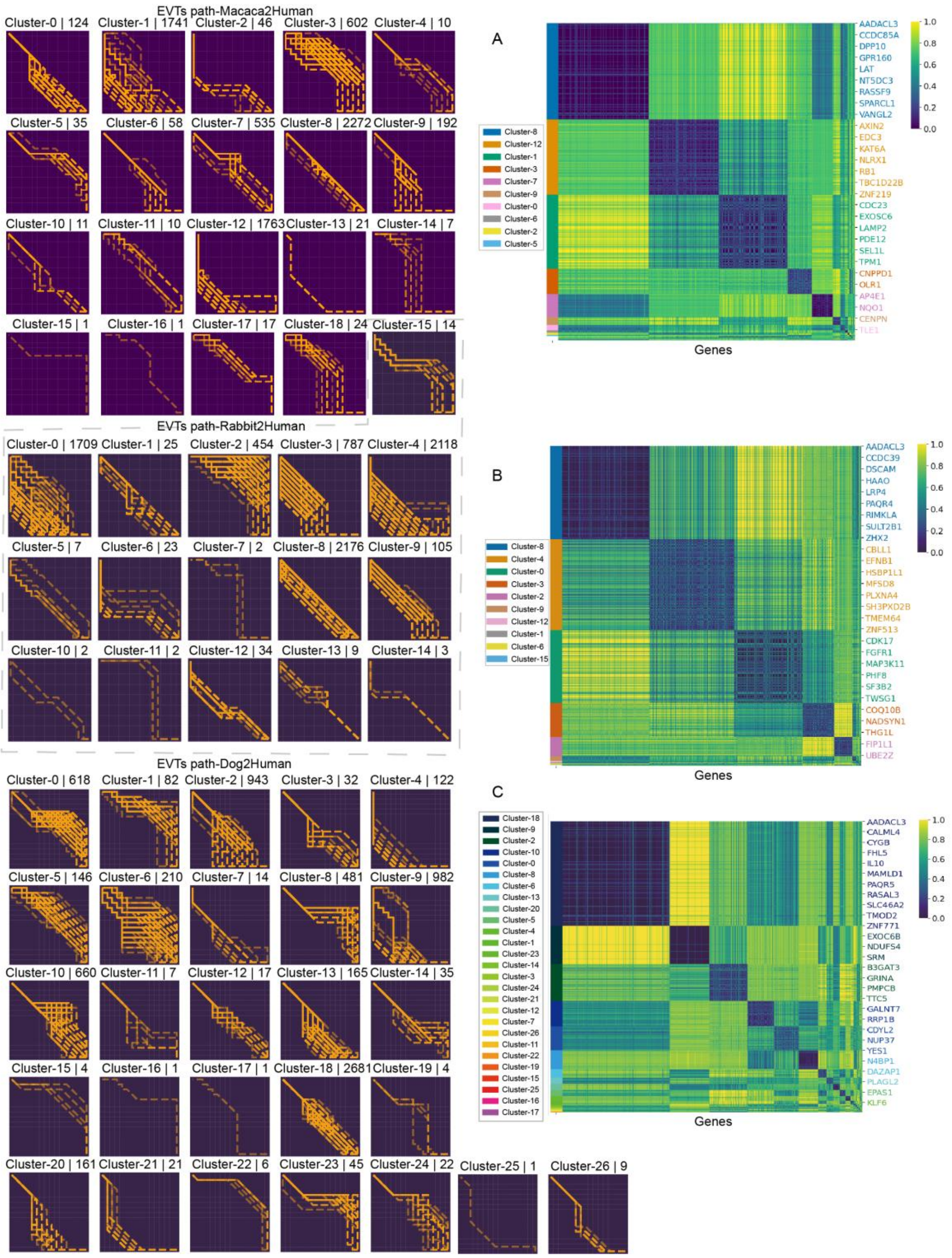

Supplementary Fig.6: Visualization of the alignment of genes grouped together in each cluster along the EVT pathway.

(A-C) The left shows the alignment trajectories of genes in different clusters, with each plot representing the pseudotime alignment of the corresponding genes between different species and humans. Dashed lines indicate differences in gene alignment between species, and solid lines close to the diagonal indicate higher conservation. The right displays annotations and the similarity matrix of highly similar genes in each cluster, with colors ranging from purple to yellow indicating levels of gene similarity.

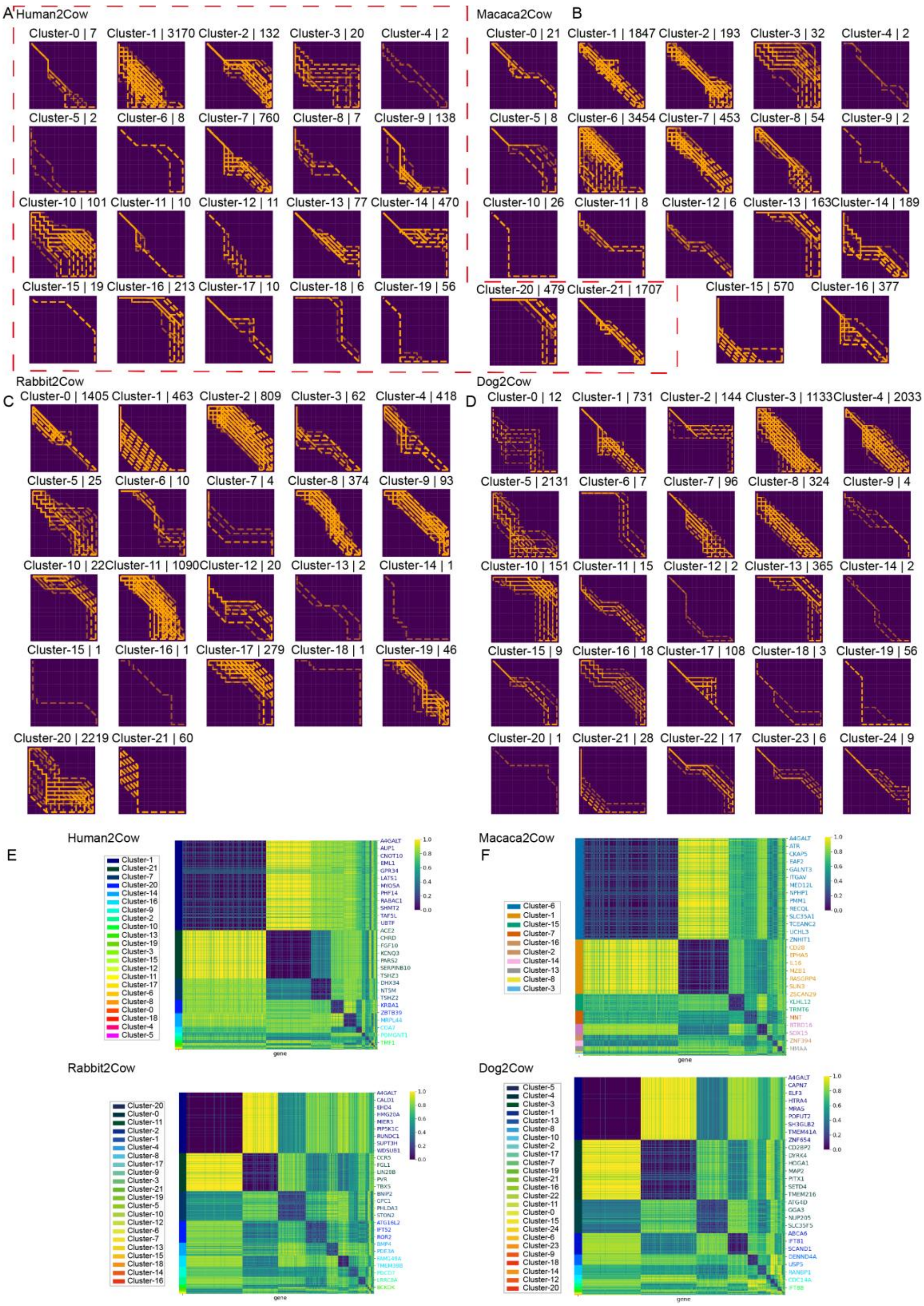

Supplementary Fig.7: Visualization of the alignment of genes grouped together in each cluster along the STB pathway.

(A-D) The alignment trajectories of genes in different clusters, with each plot representing the pseudotime alignment of the corresponding genes between different species and cows. Dashed lines indicate differences in gene alignment between species, and solid lines close to the diagonal indicate higher conservation. (E and F) Annotations and the similarity matrix of highly similar genes in each cluster are

displayed, with colors ranging from purple to yellow indicating levels of gene similarity.

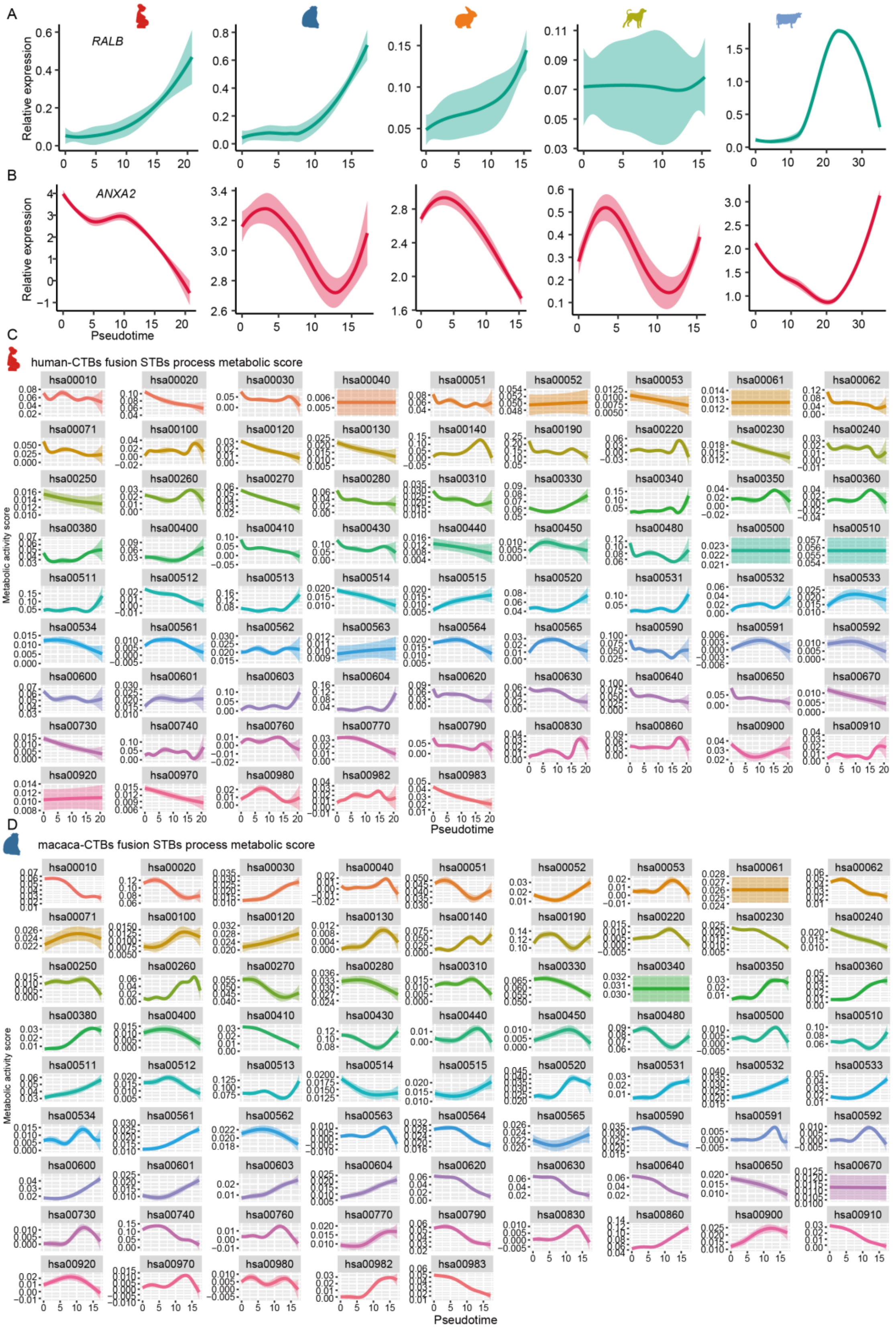

E

rabbit-CTBs fusion STBs process metabolic score

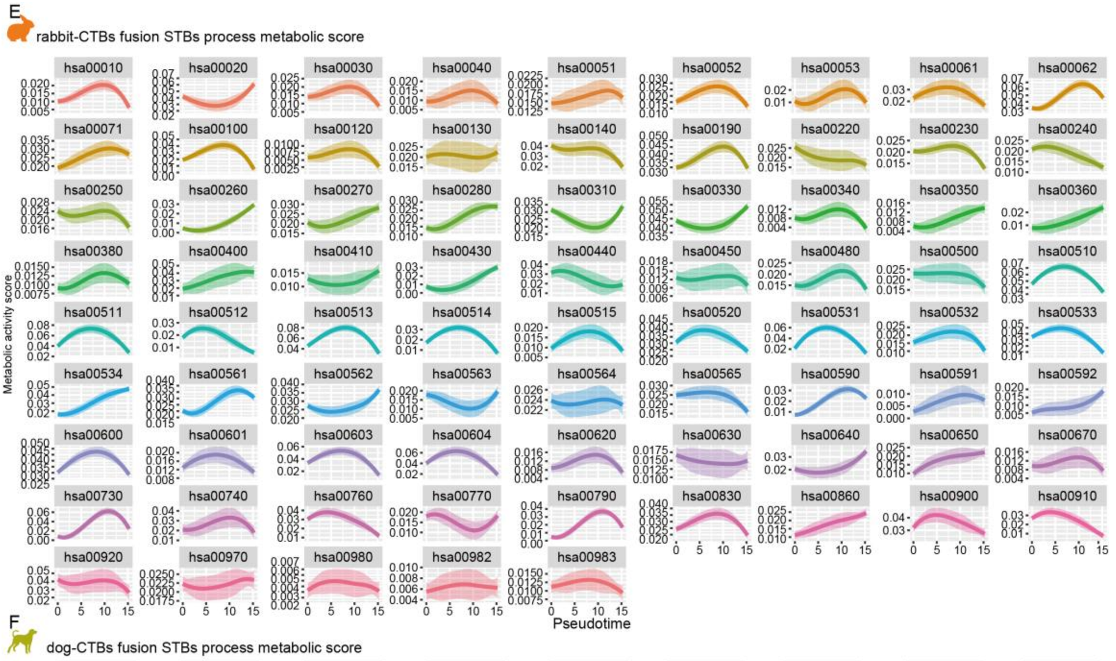

F

dog-CTBs fusion STBs process metabolic score

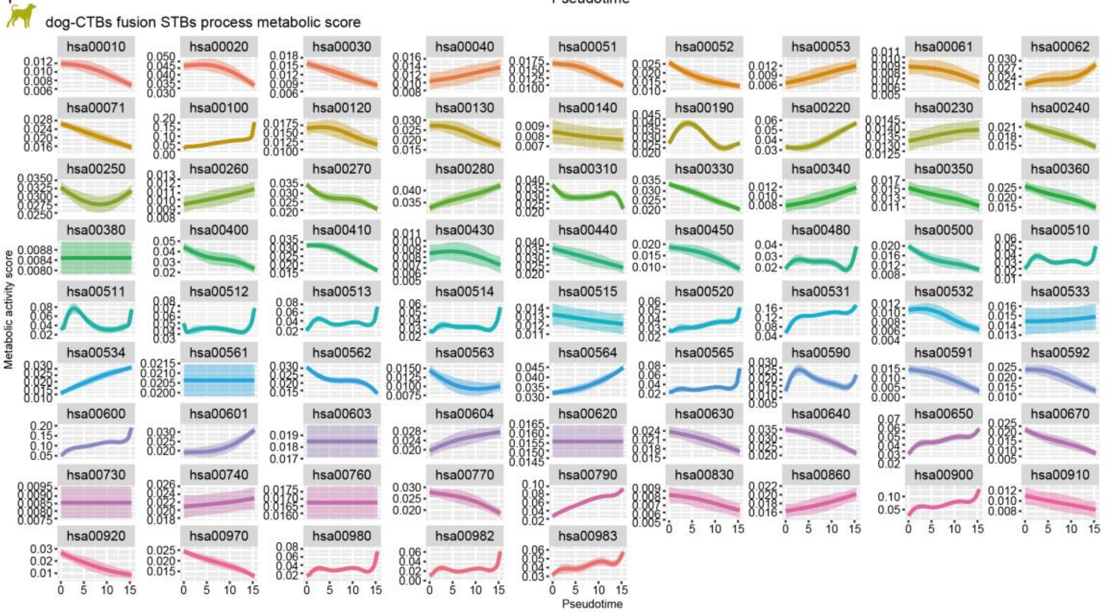

G

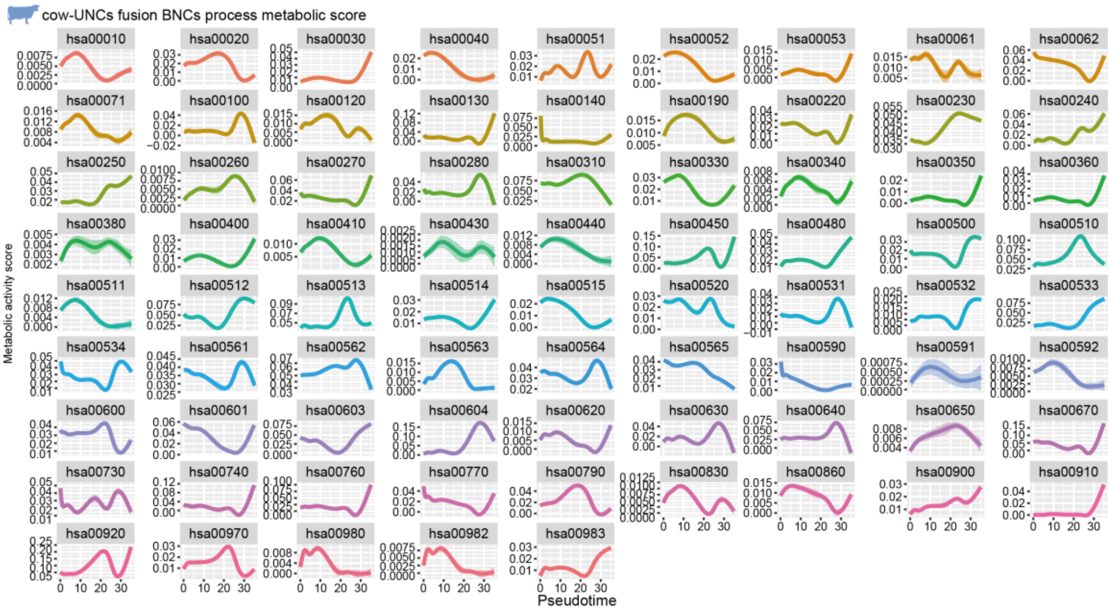

Supplementary Fig.8: Changes in gene expression and metabolic activity in the fusion metabolic pathway of trophoblasts.

(A and B) Two-line graphs showing the expression trend of *RALB* and *ANXA2* during trophoblast fusion in different species. Each curve represents the gene expression changes in a species, and the shaded area represents the confidence interval of expression. (C-G) Cross-species comparisons of changes in metabolic pathway activity during trophoblast fusion. The metabolic pathway information for humans is obtained from the KEGG database, and the KEGG pathways for other species are converted to their human orthologs. In panels A-G, lines indicate smoothed trends along pseudotime, and shaded bands represent 95% confidence intervals.

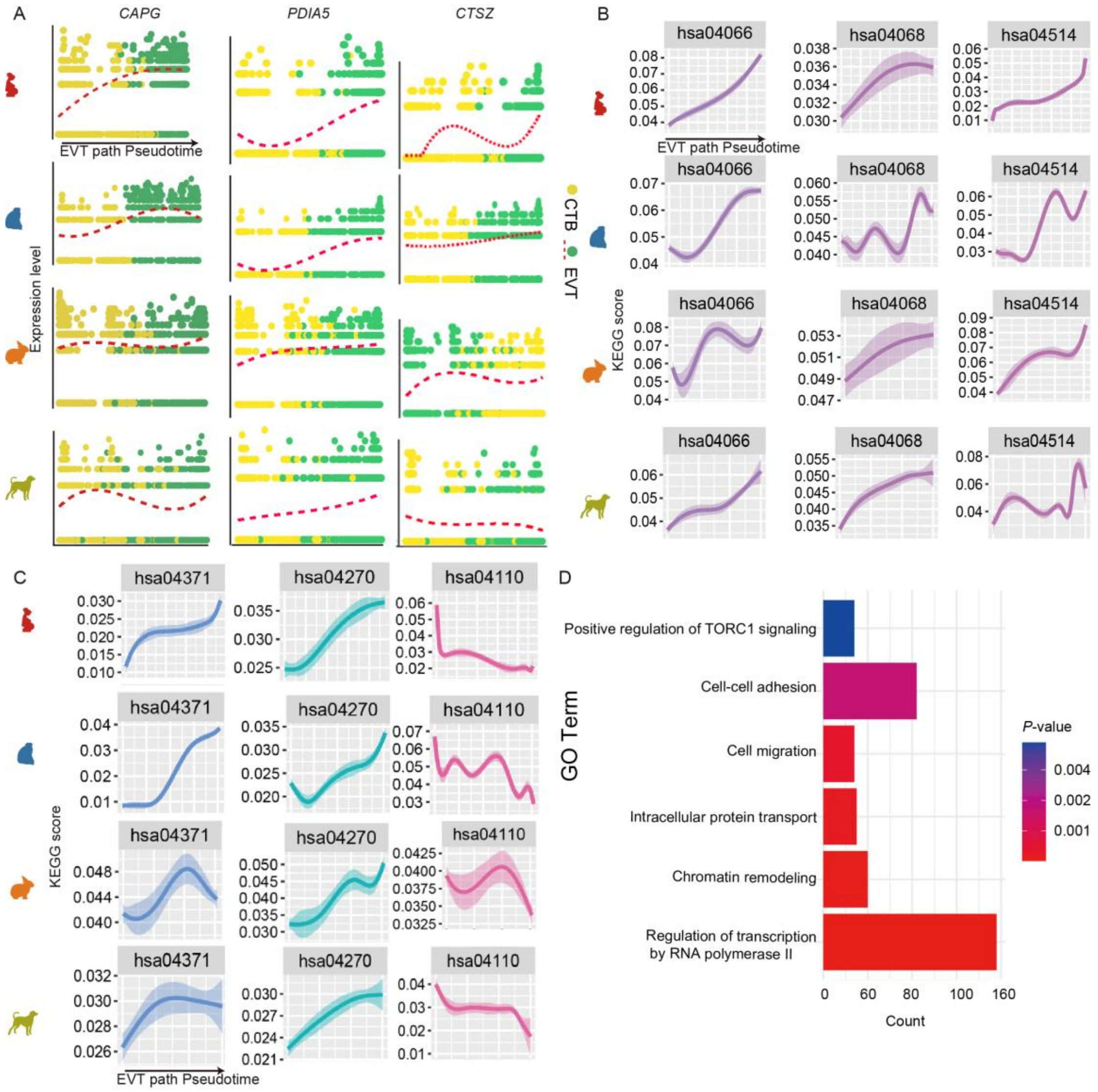

Supplementary Fig.9: Conservation and divergence of gene expression and KEGG activity in the trophoblast cells.

(A) The expression levels of *CAPG* along the pseudotime trajectory during CTB to EVT differentiation in different species (humans, macacas, rabbits, and dogs). The red dashed line represents the trend of *CAPG* expression in each species. (B and C) The changes in KEGG activity scores for three pathways along the pseudotime during CTB to EVT differentiation across different species. Each row represents the trends of KEGG activity in different species (humans, macacas, rabbits, and dogs), with the purple line showing the fitted curve. Lines indicate smoothed trends along pseudotime, and shaded bands represent 95% confidence intervals. (D) A bar plot of the GO functional analysis for genes altered in the EVT differentiation trajectory in dogs. Different colors represent different *P* values, and the length of the bars indicates the number of genes associated with each GO term. The GO enrichment analysis was performed using clusterProfiler with Benjamini-Hochberg-adjusted *P* values (Q values), and terms with Q < 0.05 were considered significant.

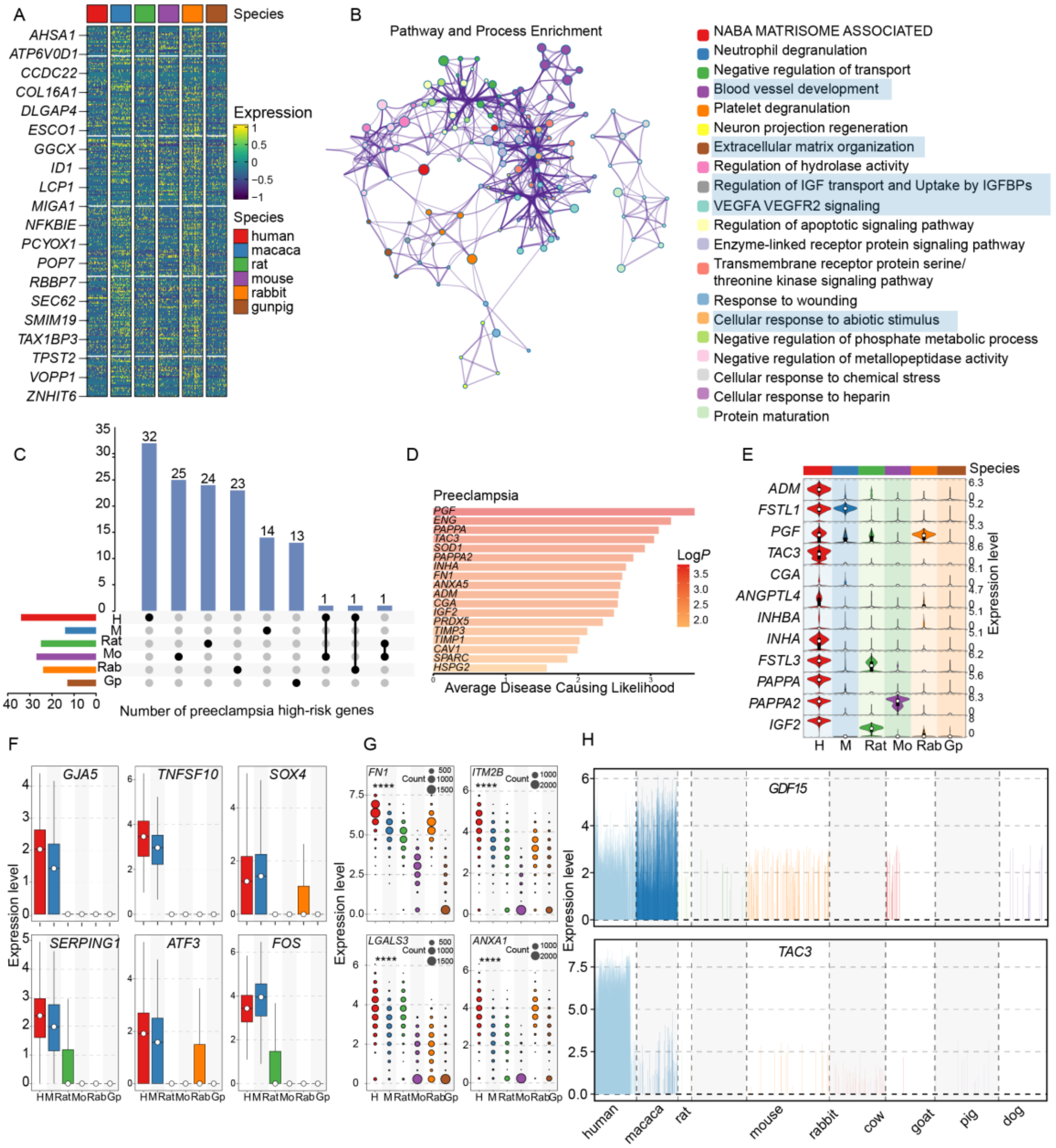

Supplementary Fig.10: Biased expression of genes in trophoblasts across species.

(A) Expression of 2,365 conserved genes in invasive trophoblasts from six species, specified in Supplementary Data 8. (B) The enrichment analysis of human biased genes using the online tool Metascape (https://metascape.org/gp/index.html#/main/step1). (C) The number and biased expression of PE risk genes in different species, specified in Supplementary Data 7. (D) Assessment of the average disease-causing likelihood of preeclampsia-associated genes using the VarElect algorithm implemented in GeneCards (https://ve.genecards.org/#input). Statistical significance was based on the *P* values returned by VarElect, shown as $-\log_{10}$(*P* value). Bar length indicates the average disease-causing likelihood score. Detailed results are provided in Supplementary Data 7. (E-G) Expression of PE risk genes in invasive trophoblasts across species. (H) Expression of PE risk genes across all trophoblast subtypes in different species.

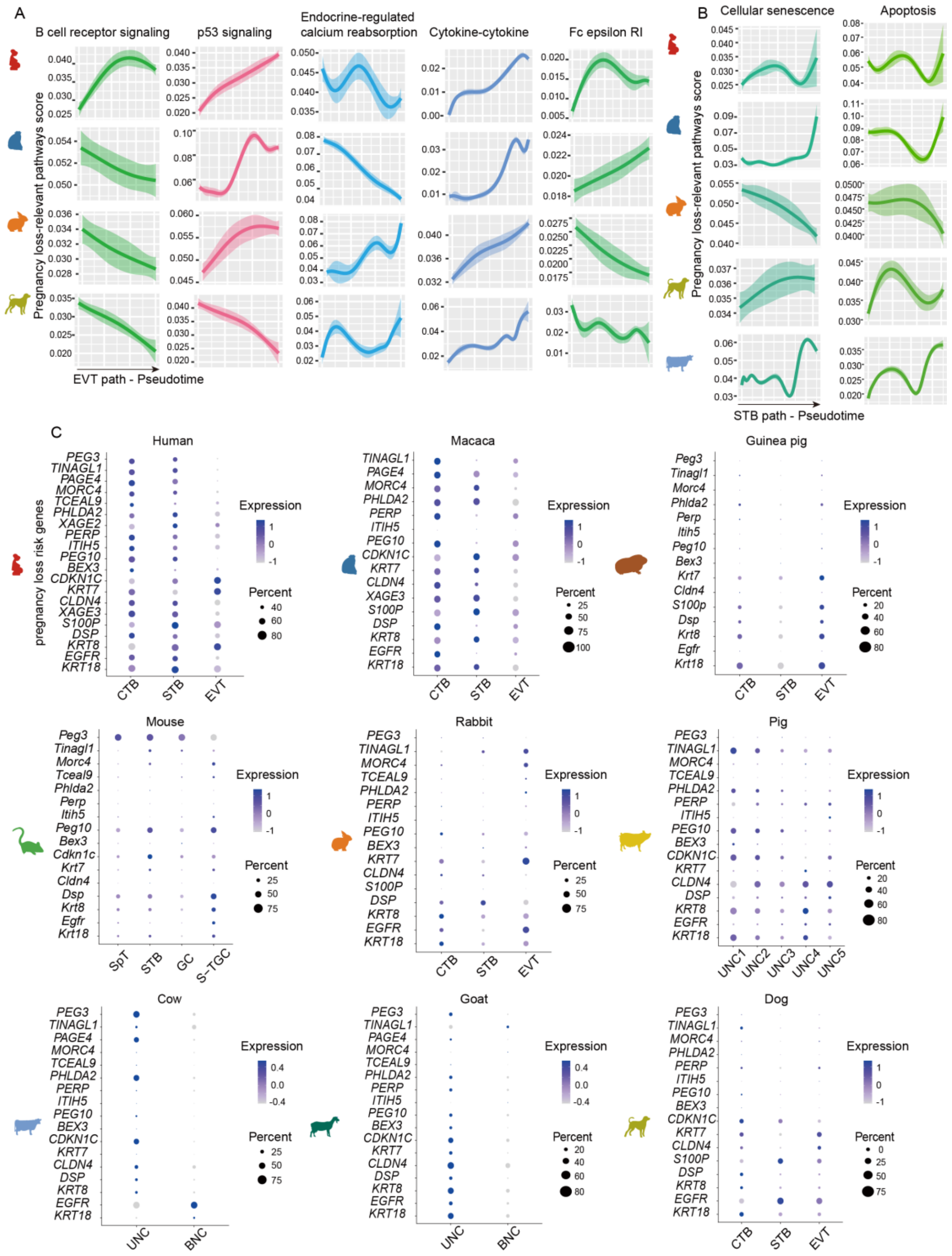

Supplementary Fig.11: The differentiation activity and expression changes of genes related to pregnancy loss across species.

(A) The changes in activity of pregnancy loss-related pathways during CTB differentiation to EVTs across different species (humans, macacas, rabbits, and dogs). (B) The changes in activity of pregnancy loss-related pathways during trophoblast fusion. (C) The expression patterns of pregnancy loss risk genes in various trophoblast subtypes across different species, specified in Supplementary Data 8. In panels A and B, lines indicate smoothed trends along pseudotime, and shaded bands represent 95% confidence intervals.

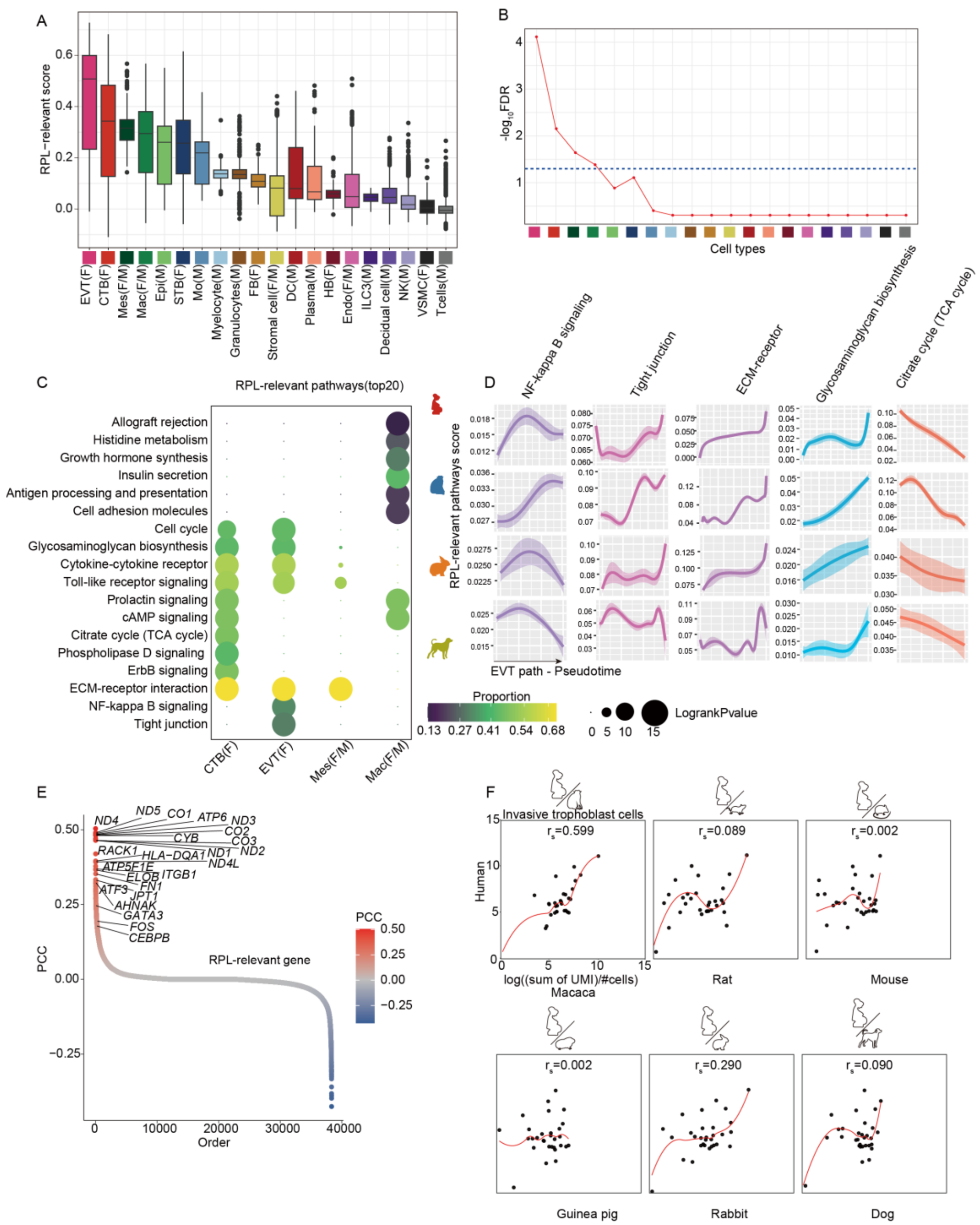

Supplementary Fig.12: GWAS combined with single-cell transcriptomic analysis revealed the placental cell types associated with recurrent pregnancy loss.

(A) Trait-relevant scores of recurrent pregnancy loss-related genes across various cell types. Box plots show the distribution of recurrent pregnancy loss-relevant scores for individual cells in each indicated cell type. Center line, median; box limits, 25th and 75th percentiles; whiskers, 1.5 × IQR; points, outliers. Scores were calculated at the single-cell level using cells derived from 23 independent placental samples. CTB, cytotrophoblasts; EVT, extravillous trophoblasts; STB, syncytiotrophoblasts; FB, fibroblasts; Endo, endothelial cells; Mac, macrophages; dNK, decidual natural killer cells; ILC, innate lymphocyte cells; VSMC, vascular smooth muscle cells; HB, Hofbauer cells; MES, mesenchymal cells; DC, dendritic cells; Epi, epithelial glandular cells. F and M in parentheses indicate the fetal and maternal origin, respectively.

(B) FDR-adjusted significance values showing the association between different placental cell types and recurrent pregnancy loss, as inferred by scPagwas. Cell type-level significance was estimated using the block bootstrap method implemented in scPagwas, and the y-axis indicates $-\log_{10}$(FDR). The dashed line marks FDR = 0.05. (C) The dot plot showing the trait-relevant pathways identified by scPagwas for three cell types. The size of the dots represents the log-ranked *P* value for each pathway, and the color intensity reflects the proportion of cells influenced by genetic effects in each pathway (the pathway-level coefficient $\beta > 0$). (D) The changes in activity of recurrent pregnancy loss-related pathways during CTB differentiation to EVTs across different species. Lines indicate smoothed trends along pseudotime, and shaded bands represent 95% confidence intervals. (E) The trait-relevant genes ranked by the Pearson correlation coefficients (PCCs) using scPagwas across all individual cells. (F) Spearman correlations of the expression (UMI counts) of top 100 susceptibility genes of recurrent pregnancy loss (orthologs, Supplementary Data 8).

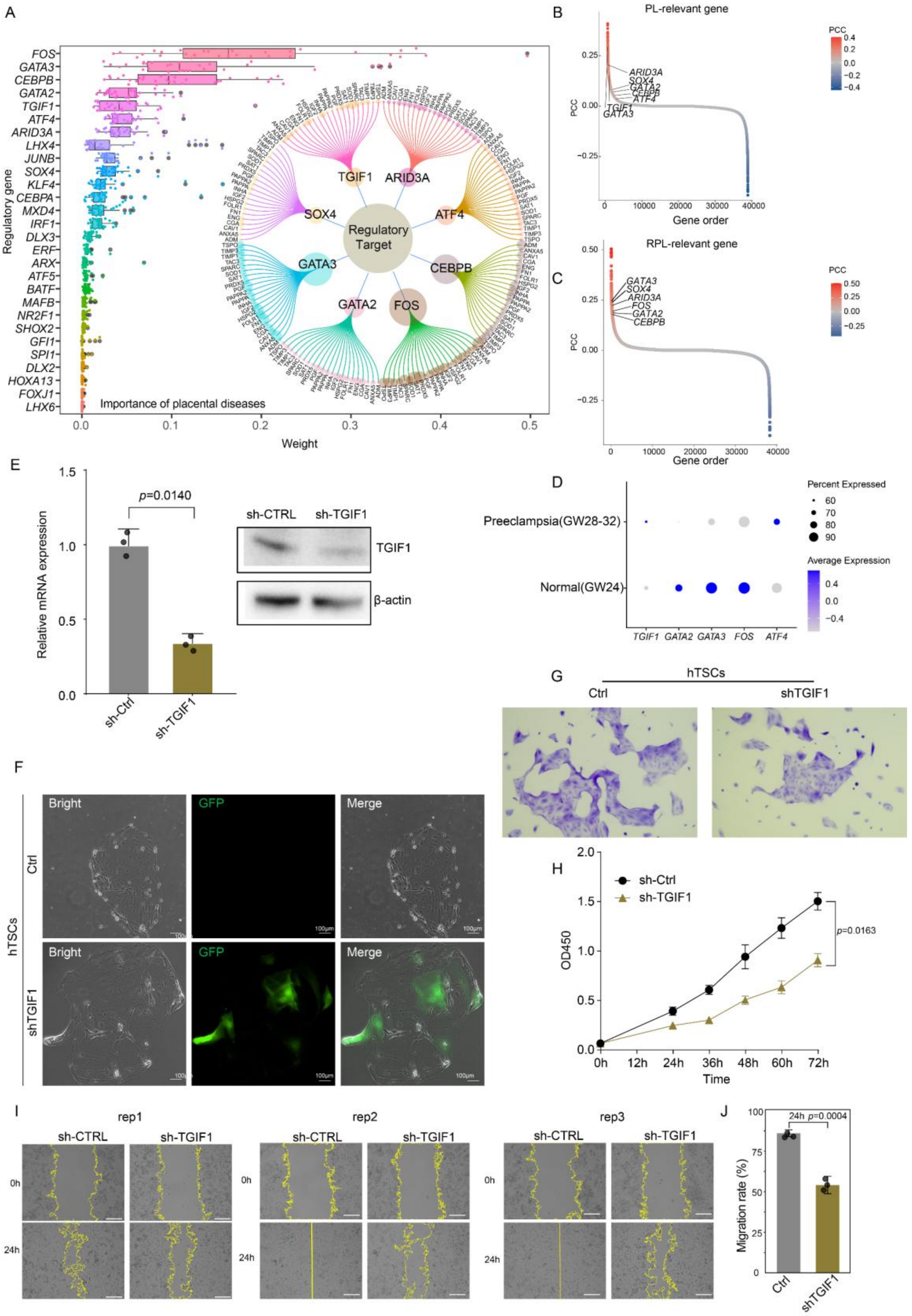

Supplementary Fig.13: The correlation between identified TFs and (recurrent) pregnancy loss.

(A) Distribution of inferred regulatory weights between 28 human-specific TFs and 404 human-biased genes in EVTs. Each dot represents the inferred regulatory weight of one TF-gene pair, and box plots summarize the distribution of weights across all human-biased genes for each TF. Center line, median;

box limits, 25th and 75th percentiles; whiskers, 1.5 × IQR; points, outliers. Regulatory weights were inferred using 3,811 EVT cells derived from six independent human placental samples. (B and C) The ranking chart showing the correlation between several identified TFs and (recurrent) pregnancy loss in GWAS results. TFs of interest, such as *GATA3*, *SOX4*, *ARID3A*, *CEBPB*, and *FOS*, are annotated. (D) Gene expression in EVTs from early PE placentas and their normal counterparts. (E) TGIF1 expression in scramble control (sh-Ctrl) and *TGIF1*-knockdown (sh-*TGIF1*) HTR-8/SVneo cells was measured by qPCR and western blot analysis. (F) Images of the transduced hTSCs. Bar = 100 μm. Dots indicate biologically independent replicates. Statistical significance was assessed using a two-sided Welch's t-test. (G) The clonogenic assay for the control and sh-*TGIF1* hTSCs. (H) The CCK-8 assay showing reduced proliferation of *TGIF1*-knockdown HTR-8/SVneo cells. Data are presented as the mean ± SEM of biological triplicates (n = 3). Statistical significance was assessed using a two-sided Welch's t-test at 72 h. (I) Representative images of the migration assay in HTR-8/SVneo cells at 0 and 24 h after scratching the cell monolayer. The yellow lines indicate the boundary of the wound area. The left and right columns show cells transduced with the scramble control (sh-Ctrl) and *TGIF1*-targeting shRNA (sh-*TGIF1*), respectively. Scale bar, 100 μm. (J) Quantification of wound closure in HTR-8/SVneo cells after 24 h, shown as the percentage of wound healing. Data are presented as the mean ± SEM of biological triplicates (n = 3). Dots indicate biologically independent replicates. Statistical significance was assessed using a two-sided Welch's t-test.

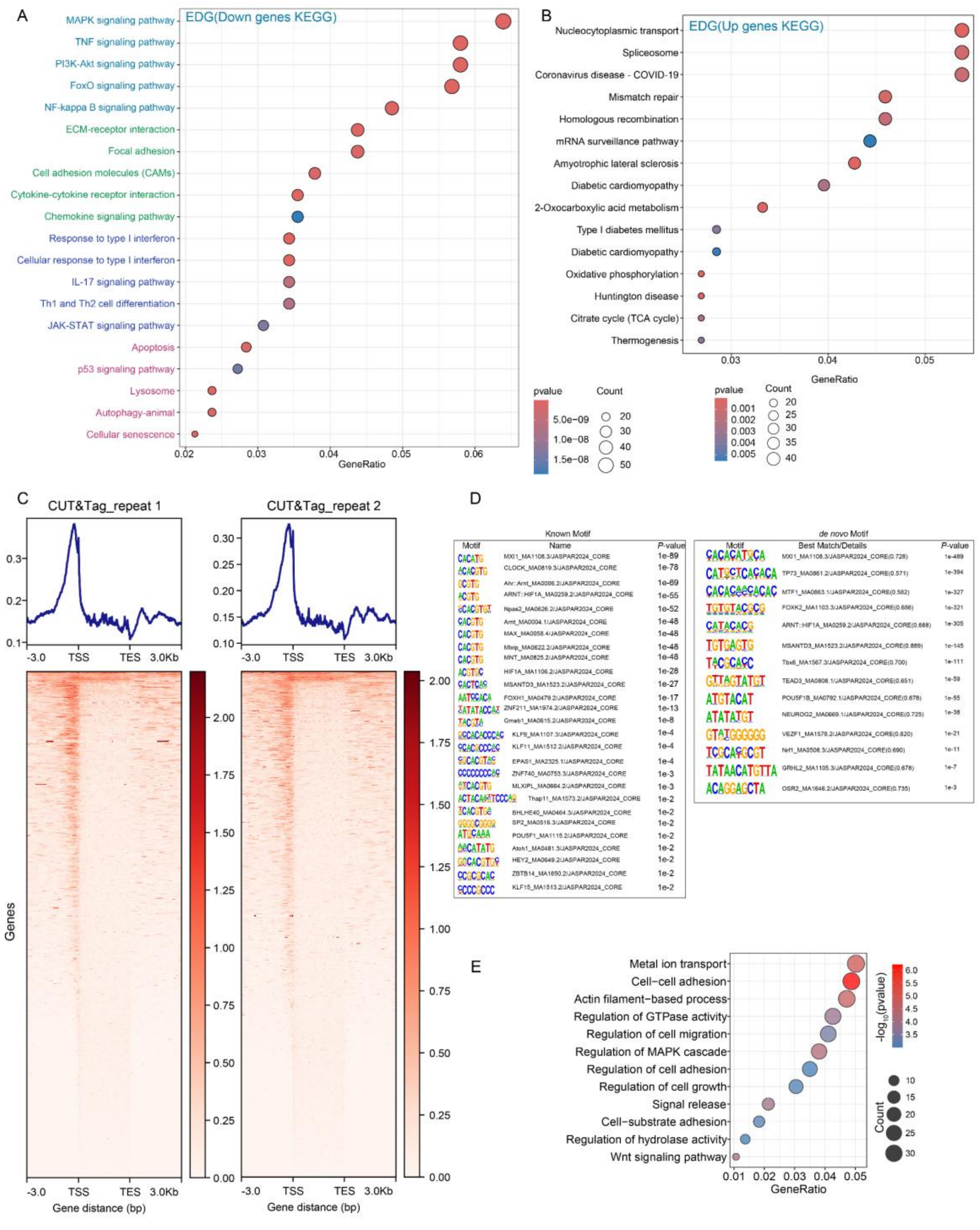

A
EDG(Down genes KEGG)
MAPK signaling pathway
TNF signaling pathway
PI3K-Akt signaling pathway
FoxO signaling pathway
NF-kappa B signaling pathway
ECM-receptor interaction
Focal adhesion
Cell adhesion molecules (CAMs)
Cytokine-cytokine receptor interaction
Chemokine signaling pathway
Response to type I interferon
Cellular response to type I interferon
IL-17 signaling pathway
Th1 and Th2 cell differentiation
JAK-STAT signaling pathway
Apoptosis
p53 signaling pathway
Lysosome
Autophagy-animal
Cellular senescence
0.02 0.03 0.04 0.05 0.06
GeneRatio
pvalue
5.0e−09
1.0e−08
1.5e−08
Count
20
30
40
50
B
EDG(Up genes KEGG)
Nucleocytoplasmic transport
Spliceosome
Coronavirus disease - COVID-19
Mismatch repair
Homologous recombination
mRNA surveillance pathway
Amyotrophic lateral sclerosis
Diabetic cardiomyopathy
2-Oxocarboxylic acid metabolism
Type I diabetes mellitus
Diabetic cardiomyopathy
Oxidative phosphorylation
Huntington disease
Citrate cycle (TCA cycle)
Thermogenesis
0.03 0.04 0.05
GeneRatio
pvalue
0.001
0.002
0.003
0.004
0.005
Count
20
25
30
35
40
C
CUT&Tag_repeat 1
CUT&Tag_repeat 2
-3.0 TSS TES 3.0Kb
Genes
Gene distance (bp)
D
Known Motif
Motif
Name
P-value
de novo Motif
Best Match/Details
E
Metal ion transport
Cell−cell adhesion
Actin filament−based process
Regulation of GTPase activity
Regulation of cell migration
Regulation of MAPK cascade
Regulation of cell adhesion
Regulation of cell growth
Signal release
Cell−substrate adhesion
Regulation of hydrolase activity
Wnt signaling pathway
0.01 0.02 0.03 0.04 0.05
GeneRatio
-log10(pvalue)
Count

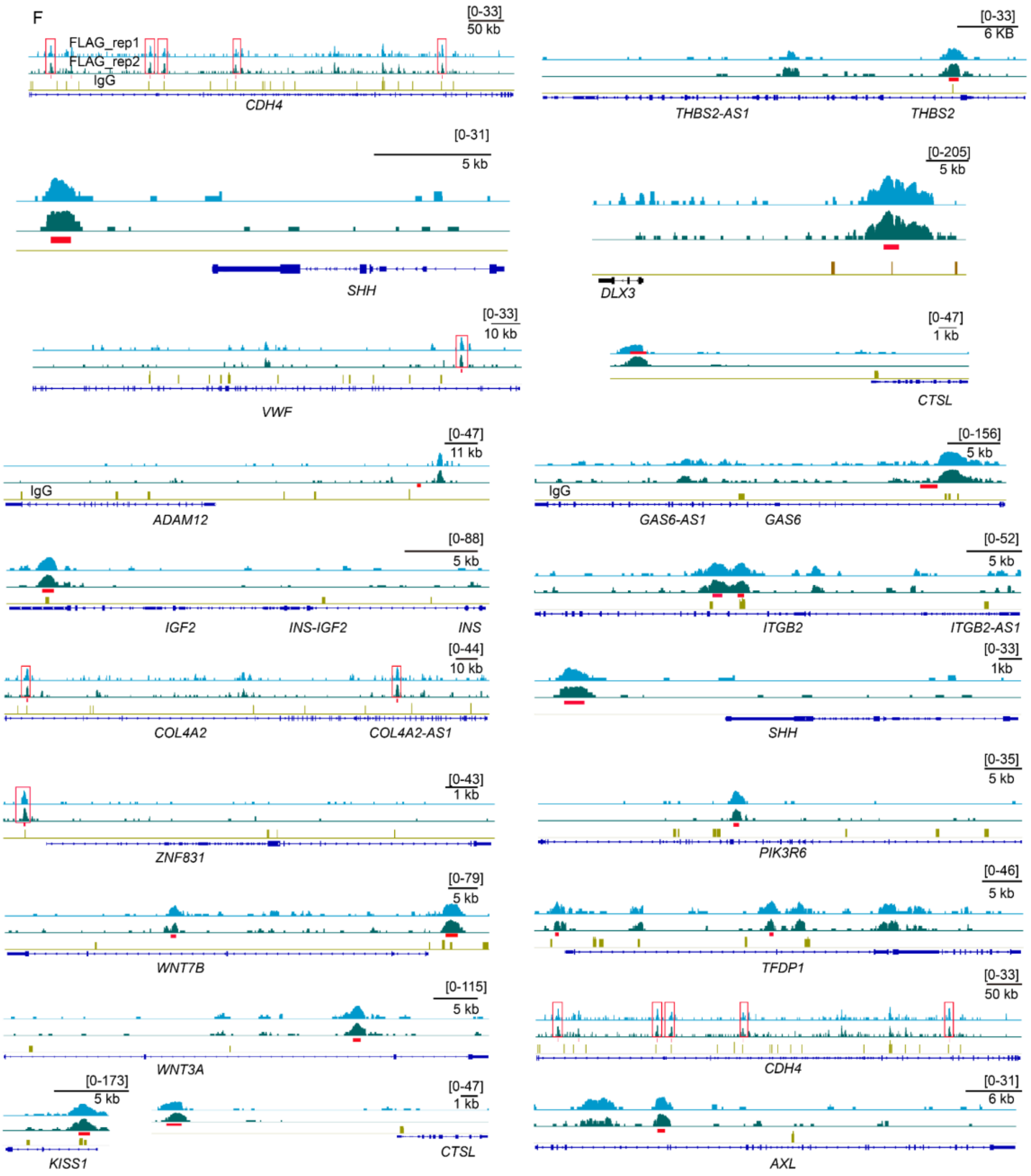

Supplementary Fig.14: Genome-wide identification of the binding motifs of TGIF1.

(A-B) KEGG enrichment analyses of downregulated (A) and upregulated (B) DEGs. The x axis indicates GeneRatio, and dot size indicates the gene count, while the color indicates the *P* value. The enrichment analysis was performed using clusterProfiler. (C) Heatmaps and average profiles of TGIF1 CUT&Tag signal intensity near transcription start sites (TSS) and transcription end sites (TES) in biological duplicates (repeat 1 and repeat 2). (D) The motif enrichment analysis of TGIF1-binding sites performed using HOMER. Known motifs were identified by comparing with reference motif databases, whereas *de novo* motifs were predicted directly from TGIF1-binding sequences and matched to the closest known motifs. *P* values indicate motif enrichment significance. (E) The GO enrichment analysis of the complete set of overlapping genes between TGIF1 peak-associated genes and DEGs, including both upregulated and downregulated ones. The x axis indicates GeneRatio, and dot size indicates the gene count, while the color indicates $-\log_{10}$(*P* value). (F) Genome browser tracks showing representative TGIF1-binding peaks.

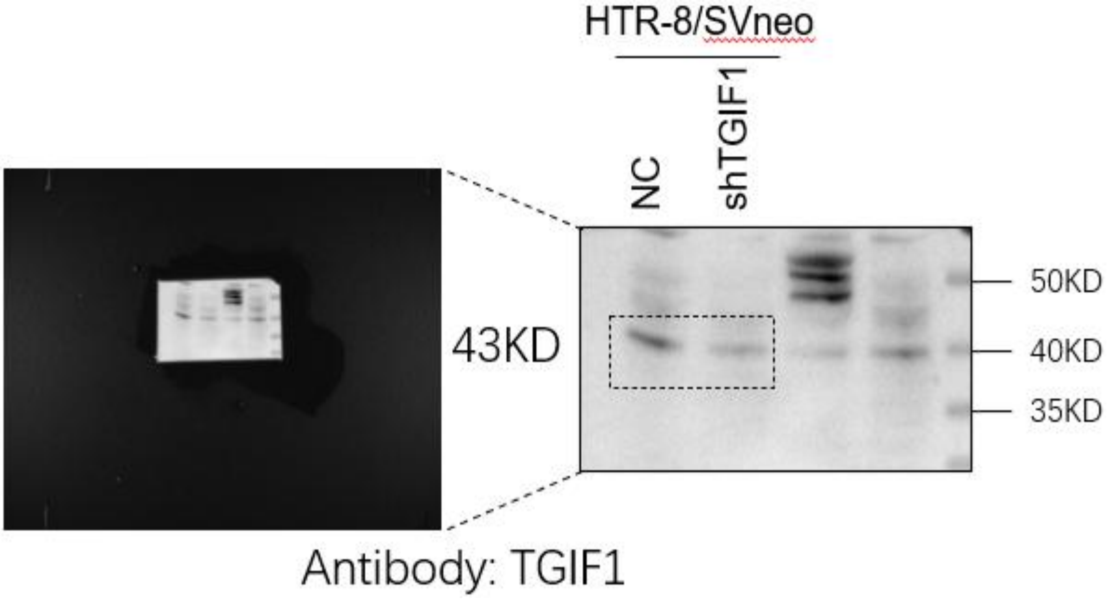

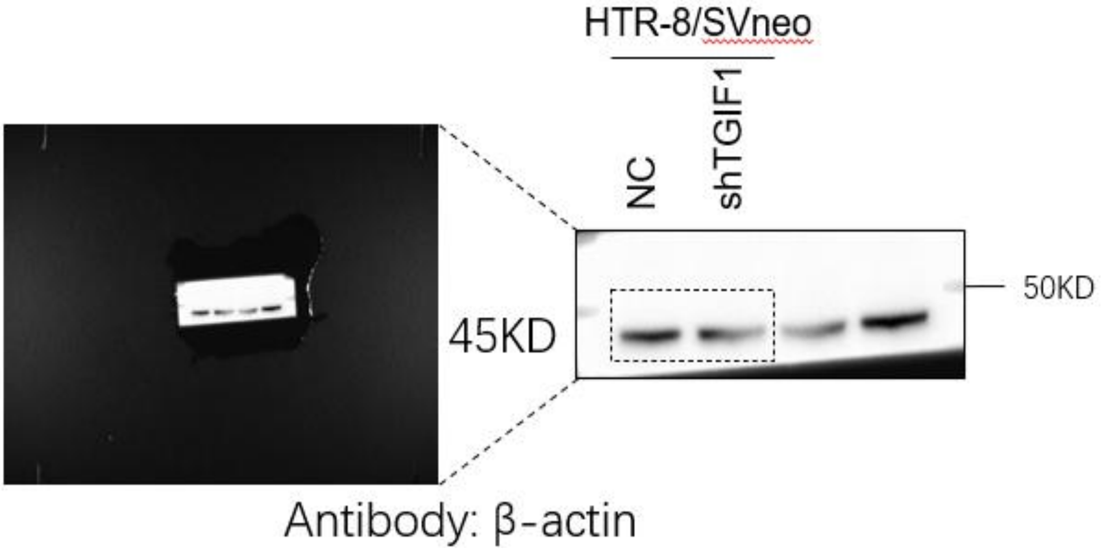

Supplementary Fig.15: Uncropped immunoblots for Supplementary Fig.13E.
