# Supplementary material for: Cross-species insights into placental evolution and diseases at the single-cell resolution
Source: Nat Commun. 2026 May 9;17:6259. doi: 10.1038/s41467-026-72652-w (PMC13377192; doi:10.1038/s41467-026-72652-w)
Supplement: Supplementary file 3 — Description of Additional Supplementary Files [file 41467_2026_72652_MOESM3_ESM.pdf]

Description of Additional Supplementary Files

File Name: Supplementary Data 1
Description: Sample information for all placental tissues used in this study, including sequencing quality metrics, cell numbers, and integration performance evaluation.

File Name: Supplementary Data 2
Description: Detailed information on animal breeding design and tissue collection.

File Name: Supplementary Data 3
Description: Marker genes for all clusters identified from the newly generated datasets in this study

File Name: Supplementary Data 4
Description: Genes associated with trophoblast pseudotime trajectories across all species, together with clustering results and GO enrichment analyses for the clusters.

File Name: Supplementary Data 5
Description: Conserved and divergent genes during trophoblast differentiation, together with GO enrichment analysis results.

File Name: Supplementary Data 6
Description: Conserved and divergent gene sets in invasive EVT cells of discoid placentas across species, together with their enrichDisGeNET enrichment results.

File Name: Supplementary Data 7
Description: Summary information for GWAS datasets of recurrent pregnancy loss and their association analysis results.

File Name: Supplementary Data 8
Description: Transcription factors showing significant activity in invasive trophoblast cells across all discoid placental species.

File Name: Supplementary Data 9
Description: Summary statistics for RNA-seq and CUT&Tag data generated from human trophoblast stem cells.
