# Supplementary material for: Cross-species insights into placental evolution and diseases at the single-cell resolution
Source: Nat Commun. 2026 May 9;17:6259. doi: 10.1038/s41467-026-72652-w (PMC13377192; doi:10.1038/s41467-026-72652-w)
Supplement: Supplementary file 5 — Supplementary Data 2 [file 41467_2026_72652_MOESM5_ESM.docx]

**Animals Breeding Design and Tissue Collection**

**Animals and Tissue Collection**

In this study, maternal-fetal interface samples (including placenta, endometrium, and myometrium) were collected from multiple mammalian species, specifically guinea pigs (*Cavia porcellus*), rabbits (*Oryctolagus cuniculus*), dogs (*Canis lupus familiaris*), cows (*Bos taurus*), goats (*Capra hircus*), and pigs (*Sus scrofa*). The specific collection process is as follows:

**Animal Breeding Design and Preparation (guinea pigs, rabbits, and dogs):** For the breeding experiments of guinea pigs, rabbits, and dogs, the research team carefully designed a schedule for sexual maturity and breeding to ensure physiological consistency across the samples. Guinea pigs reach sexual maturity at approximately 3 months of age, rabbits at around 4 months, and dogs at about 8 months. Upon reaching sexual maturity, male and female animals were housed separately to avoid premature mating, allowing female animals to stabilize their hormonal cycles in an independent environment. During this stage, female animals were housed in individual cages, ensuring they received adequate nutrition and a comfortable living environment to minimize external factors affecting their physiological states. Before entering the breeding phase, female animals were introduced to the male room for sexual preconditioning, where they were exposed to the scent and sounds of male animals for several days to a week, depending on each species' response and maturity rate. This simulated natural mating environments and stimulated hormonal responses. After preconditioning, the first mating occurred at approximately 3.5 months for guinea pigs, 5.5 months for rabbits, and 8.5 months for dogs. During mating, non-cycling female animals were first introduced to the male room for one day to familiarize themselves with the environment and male scent, and then moved to a cage previously used by a prospective male partner for five days to further enhance mating likelihood and ensure precise timing of conception.

**Mating and Pregnancy Monitoring:** After mating, the male and female animals were placed in a specially designed mating cage and continuously monitored via a round-the-clock video surveillance system. The surveillance system captured the exact time of mating, ensuring no disturbance to the animals during this period. If multiple copulations were observed, the first copulation was always used as the standard to calibrate 0 dpc (days post-coitum). To ensure data accuracy, each individual was under strict physiological monitoring throughout the entire pregnancy process, including continuous monitoring of heart rate, respiratory rate, and body temperature, ensuring their health and comfort during the entire gestation period. Post-mating care procedures, including providing a balanced diet, appropriate ambient temperature, and humidity control, were conducted within standardized animal facilities at the Experimental Animal Center of Northwest A&F University, adhering strictly to animal welfare regulations. Sample Collection: During the late stages of pregnancy, to ensure the collection of high-quality placental samples for single-nuclear RNA sequencing (snRNA-seq), the research team conducted sample collection at the optimal time point. For guinea pigs and rabbits, sample collection methods were identical, with placental samples being collected via cesarean section at 25.5 days (n=3) and 45.5 days (n=3) of pregnancy, respectively. Prior to surgery, the animals were administered an appropriate dose of isoflurane anesthesia to ensure pain-free sample collection and minimize stress responses. During surgery, the animals' vital signs, such as heart rate, blood pressure, and oxygen saturation, were continuously monitored to ensure they remained in a stable physiological state throughout the procedure. During the surgery, sterile surgical tools and operating tables were used to prevent any external contamination of the samples. After the cesarean section, the placental samples were carefully extracted and immediately placed in pre-chilled sterile saline for preliminary processing. To ensure sample integrity, the placental samples were transported on ice to the laboratory for further processing, including cell isolation and RNA extraction. For dogs, the sample collection was performed at 50.5 days of pregnancy. The dogs were similarly administered isoflurane anesthesia before surgery, and their vital signs were continuously monitored by a professional veterinary team throughout the procedure. During surgery, the entire uterus was carefully removed, and placental samples were collected by incising the uterine wall. After sample collection, the surgical wound was immediately sutured to ensure the mother did not suffer further health risks due to the surgery. The entire process was conducted in a nationally accredited animal hospital, with all procedures performed by professionally trained veterinarians to minimize harm and stress to the animals and ensure postoperative recovery of the mother.

**Animal Breeding Design and Preparation (Cow, Goat, and Pig):** In a controlled environment, a group of healthy female livestock, including Holstein cows, Saanen dairy goats, and Duroc pigs, were selected for artificial insemination. Thirty days post-insemination, pregnancy status was assessed via ultrasound or palpation to confirm successful fertilization. Sample collection was conducted in strict accordance with the guidelines set forth by the Institutional Animal Care and Use Committee (IACUC) at Northwest A&F University. Holstein cows (N=2), Saanen goats (N=5), and Duroc pigs (N=3) were humanely slaughtered at commercial abattoirs on gestational days 240, 120-140, and 100, respectively. Following euthanasia, the reproductive tracts were immediately retrieved and transported to the laboratory under cold conditions for dissection. In the laboratory, the reproductive organs were carefully dissected to collect maternal-fetal interface tissues. The areas adjacent to the fetus were dissected to isolate the cotyledon and caruncle components. These tissues were meticulously separated using sterile dissection tools to avoid cross-contamination and were subsequently snap-frozen in liquid nitrogen for preservation. Additionally, the inter-cotyledonary placenta adjacent to the fetus was collected and similarly snap-frozen in liquid nitrogen. All snap-frozen tissues were then transferred to storage at -80°C for long-term preservation until further analysis. Fresh cotyledon and inter-cotyledonary placental tissues were reserved for immediate single-nucleus isolation and subsequent analyses
