# Supplementary material for: Cross-species insights into placental evolution and diseases at the single-cell resolution
Source: Nat Commun. 2026 May 9;17:6259. doi: 10.1038/s41467-026-72652-w (PMC13377192; doi:10.1038/s41467-026-72652-w)
Supplement: Supplementary file 13 — Reporting Summary [file 41467_2026_72652_MOESM13_ESM.pdf]

nature portfolio

Corresponding author(s): Yu Jiang, Yi Zheng, Zhenyu Xiao,Xihong Wang

Last updated by author(s): April 12, 2026

Reporting Summary

Nature Portfolio wishes to improve the reproducibility of the work that we publish. This form provides structure for consistency and transparency in reporting. For further information on Nature Portfolio policies, see our Editorial Policies and the Editorial Policy Checklist.

Statistics

For all statistical analyses, confirm that the following items are present in the figure legend, table legend, main text, or Methods section.

n/a	Confirmed	
☐	☒	The exact sample size (*n*) for each experimental group/condition, given as a discrete number and unit of measurement
☐	☒	A statement on whether measurements were taken from distinct samples or whether the same sample was measured repeatedly
☐	☒	The statistical test(s) used AND whether they are one- or two-sided<br>*Only common tests should be described solely by name; describe more complex techniques in the Methods section.*
☒	☐	A description of all covariates tested
☐	☒	A description of any assumptions or corrections, such as tests of normality and adjustment for multiple comparisons
☐	☒	A full description of the statistical parameters including central tendency (e.g. means) or other basic estimates (e.g. regression coefficient) AND variation (e.g. standard deviation) or associated estimates of uncertainty (e.g. confidence intervals)
☐	☒	For null hypothesis testing, the test statistic (e.g. *F*, *t*, *r*) with confidence intervals, effect sizes, degrees of freedom and *P* value noted<br>*Give P values as exact values whenever suitable.*
☒	☐	For Bayesian analysis, information on the choice of priors and Markov chain Monte Carlo settings
☒	☐	For hierarchical and complex designs, identification of the appropriate level for tests and full reporting of outcomes
☐	☒	Estimates of effect sizes (e.g. Cohen's *d*, Pearson's *r*), indicating how they were calculated

Our web collection on statistics for biologists contains articles on many of the points above.

Software and code

Policy information about availability of computer code

Data collection	Libraries for newly generated snRNA-seq data were prepared using the Chromium Single Cell 3' GEM, Library & Gel Bead Kit v3 (10x Genomics, PN-1000075), converted using the MGI Easy Universal DNA Library Preparation Kit, and sequenced on a BGISEQ-500 platform. Microscopy images were acquired using a ZEISS LSM 880 confocal microscope and processed with ZEN (black) software. No custom code was used for data collection.
Data analysis	Data analysis was performed using publicly available software and packages described in the Methods, including PISA for FASTQ reformatting, STAR for alignment, DropletUtils for empty-droplet removal, Souporcell for genotype deconvolution, Seurat (v4.4.0) for quality control, normalization, integration and clustering, bc3net for enrichment testing, EggNOG-mapper and EggNOG for orthology analysis, DESeq2 (v3.40.0), SCENIC (v0.11.0) with arboreto (v0.1.3), loomR (v0.2.1.9000), Scanpy, Slingshot, Monocle2, CytoTRACE, KEGGREST (v1.38.0), AUCell, GENIE3 (v1.24.0), fastp, FeatureCounts, Bowtie2 (v2.4.4), Trimmomatic (v0.39), Samtools (v1.13), MACS2 (v2.1.1), IGV (v2.15.2), Metascape, and standard R/Python functions. No custom code central to the conclusions of this study was generated.

For manuscripts utilizing custom algorithms or software that are central to the research but not yet described in published literature, software must be made available to editors and reviewers. We strongly encourage code deposition in a community repository (e.g. GitHub). See the Nature Portfolio guidelines for submitting code & software for further information.

Data

Policy information about availability of data

All manuscripts must include a data availability statement. This statement should provide the following information, where applicable:

- Accession codes, unique identifiers, or web links for publicly available datasets
- A description of any restrictions on data availability
- For clinical datasets or third party data, please ensure that the statement adheres to our policy

The raw and processed data generated in this study have been deposited in the NCBI database under the accession code PRJNA1177647. Human placental scRNA-seq data used in this study were obtained from the European Genome-phenome Archive (EGA, https://www.ebi.ac.uk/ega/; accession EGAS00001002449). Mouse placental snRNA-seq data were retrieved from GEO (accession: GSE156125). Macaca placental datasets were obtained from GEO (accessions: GSE180637), and the rat scRNA-seq data were also sourced from GEO (accession: GSE206086). GWAS summary statistics for pregnancy loss were downloaded from deCODE (https://www.decode.com/summarydata/), and those for multiple consecutive miscarriages were obtained from Estonian Biobank (http://www.geenivaramu.ee/tools/misc_sumstats.zip). All other relevant data supporting the key findings of this study are provided in the article or as Supplementary Data. Source data are provided with this paper.

Research involving human participants, their data, or biological material

Policy information about studies with human participants or human data. See also policy information about sex, gender (identity/presentation), and sexual orientation and race, ethnicity and racism.

Reporting on sex and gender	This study did not involve recruitment of human participants. Human-derived materials included previously established human trophoblast stem cell (hTSC) lines derived from first-trimester placentas or blastocysts, an authenticated HTR-8/SVneo cell line, and publicly available human placental single-cell datasets. Sex/gender information was not uniformly available for all human-derived materials and was therefore not analysed as a variable in this study.
Reporting on race, ethnicity, or other socially relevant groupings	Not collected and not used as a proxy or covariate. The study does not involve comparisons across socially defined groups.
Population characteristics	Human-derived materials used in this study included previously established hTSC lines derived from first-trimester placentas or blastocysts, an authenticated HTR-8/SVneo cell line, and publicly available human placental single-cell datasets spanning the first, second, and third trimesters. No clinical interventions or patient outcomes were analyzed.
Recruitment	No human participants were recruited for this study. Human-derived materials consisted of previously established hTSC lines, an authenticated human cell line, and publicly available datasets.
Ethics oversight	No new human participants were recruited for this study. Human-derived materials included previously established hTSC lines derived from first-trimester placentas or blastocysts, an authenticated HTR-8/SVneo cell line, and publicly available human placental datasets. No clinical interventions or patient outcomes were analyzed.

Note that full information on the approval of the study protocol must also be provided in the manuscript.

Field-specific reporting

Please select the one below that is the best fit for your research. If you are not sure, read the appropriate sections before making your selection.

[x] Life sciences [ ] Behavioural & social sciences [ ] Ecological, evolutionary & environmental sciences

For a reference copy of the document with all sections, see nature.com/documents/nr-reporting-summary-flat.pdf

Life sciences study design

All studies must disclose on these points even when the disclosure is negative.

Sample size	No formal statistical methods were used to predetermine sample size. For cross-species placental sampling, the number of biological replicates was chosen based on common practice in the field, sample availability, developmental-stage matching, and feasibility of collection across species. For cell-based validation experiments, sample sizes were selected based on common practice in the field, and at least three biologically independent experiments were performed for quantitative analyses. These sample sizes were considered sufficient to detect reproducible differences between groups.
Data exclusions	For newly generated and public single-cell/single-nucleus datasets, samples or cells failing predefined quality-control criteria were excluded. Cells with fewer than 500 detected genes or greater than 20% mitochondrial transcripts were removed, and doublets/ambient RNA-contaminated droplets were filtered using Scrublet and Souporcell as described in the Methods. One rat E19.5 sample (GSM8016898_19_5_7-PD) was excluded because of relatively low cell yield and reduced data quality. No other data were excluded unless stated in the Methods.
Replication	Biological replication was incorporated throughout the study. Cross-species placental analyses included independent biological samples for each species and developmental stage where available. Cell-based validation experiments were independently repeated, with quantitative assays generally performed using at least three biologically independent experiments. Representative microscopy images and blotting results are indicated in the figure legends together with the number of independent repetitions with similar results.

Randomization	Randomization was not relevant for the cross-species placental analyses because samples were collected from predefined species and gestational stages rather than being prospectively assigned to experimental groups. To reduce potential confounding, samples were collected at matched developmental stages within each species and processed using standardized procedures. For cell-based experiments, samples were assigned to predefined treatment groups (for example, Ctrl or shTGIF1) and processed in parallel under the same experimental conditions.
Blinding	Investigators were not blinded to group allocation during data collection or analysis. Blinding was not applied because the study was primarily based on predefined species/sample groups, publicly available datasets, and objective single-cell/single-nucleus sequencing, computational, and molecular readouts. For cell-based experiments, predefined treatment groups were processed in parallel under the same experimental conditions.

Reporting for specific materials, systems and methods

We require information from authors about some types of materials, experimental systems and methods used in many studies. Here, indicate whether each material, system or method listed is relevant to your study. If you are not sure if a list item applies to your research, read the appropriate section before selecting a response.

Materials & experimental systems

n/a	Involved in the study
☐	☒ Antibodies
☐	☒ Eukaryotic cell lines
☒	☐ Palaeontology and archaeology
☐	☒ Animals and other organisms
☒	☐ Clinical data
☒	☐ Dual use research of concern
☒	☐ Plants

Methods

n/a	Involved in the study
☒	☐ ChIP-seq
☒	☐ Flow cytometry
☒	☐ MRI-based neuroimaging

Antibodies

Antibodies used	Primary antibodies used in this study were rabbit anti-TGIF (Abcam, ab52955; 1:50 for CUT&Tag and 1:1000 for immunofluorescence), mouse anti-TGIF (Santa Cruz, sc-17800; 1:1000 for western blotting), rabbit anti-β-actin (Cell Signaling Technology, #4970; 1:1000 for western blotting), and mouse anti-HLA-G (Santa Cruz, sc-21799; 1:1000 for immunofluorescence). An IgG control antibody was used for CUT&Tag (Vazyme, RA1008-01; 1:50).
Validation	Primary antibodies used in this study were validated based on manufacturer documentation, prior literature, and consistency with the expected signal patterns in our experiments. For western blotting, mouse anti-TGIF (Santa Cruz, sc-17800) detected a band at the expected molecular weight, and the signal was reduced upon TGIF1 knockdown in hTSCs, supporting antibody specificity. Rabbit anti-β-actin (Cell Signaling Technology, #4970) was used as a loading control. For CUT&Tag and immunofluorescence, rabbit anti-TGIF (Abcam, ab52955) and mouse anti-HLA-G (Santa Cruz, sc-21799) were used according to manufacturer recommendations and published protocols. Relevant details are provided in the Methods sections “CUT&Tag assay and data analysis”, “Western blotting”, and “Immunofluorescence staining”.

Eukaryotic cell lines

Policy information about cell lines and Sex and Gender in Research

Cell line source(s)	Human trophoblast stem cells (hTSCs) were previously established from human preimplantation embryos as described in previous studies and were generously provided by Dr. Yongli Shan (Guangzhou Institutes of Biomedicine and Health, Chinese Academy of Sciences). HTR-8/SVneo cells were obtained from the National Collection of Authenticated Cell Cultures. HEK293T cells were used for lentiviral packaging.
Authentication	HTR-8/SVneo cells were obtained from the National Collection of Authenticated Cell Cultures. The hTSCs used in this study were previously established and obtained from an external source as described above. No additional cell line authentication was performed by the authors
Mycoplasma contamination	negative
Commonly misidentified lines (See ICLAC register)	No commonly misidentified cell lines listed by ICLAC were used in this study.

Animals and other research organisms

Policy information about studies involving animals; ARRIVE guidelines recommended for reporting animal research, and Sex and Gender in Research

Laboratory animals	Pregnant guinea pigs (Cavia porcellus), rabbits (Oryctolagus cuniculus), dogs (Canis lupus familiaris), cows (Bos taurus), goats (Capra hircus), and pigs (Sus scrofa) were used for placental tissue collection. For guinea pigs, rabbits, and dogs, timed pregnancies were

	established by controlled mating and samples were collected at gestational day 25.5, 45.5, and 50.5, respectively. For cows, goats, and pigs, pregnancies were established by artificial insemination and samples were collected at gestational day 240, 120–140, and 100, respectively. Detailed information on species, breed/strain, age, sex, and sample numbers is provided in Supplementary Data 1 and Supplementary Data 2.
Wild animals	This study did not involve wild animals.
Reporting on sex	The animal experiments involved pregnant female animals for placental tissue collection. Sex was therefore inherent to the study design for maternal samples. Sex was not analysed as an independent variable because the study focused on cross-species placental cell states and trophoblast biology, and fetal sex information was not uniformly available across all newly generated and publicly available datasets.
Field-collected samples	This study did not involve field-collected samples.
Ethics oversight	All animal experimental procedures and sampling protocols were reviewed and approved by the Institutional Animal Care and Use Committee (IACUC) of Northwest A&F University (Approval No. XN2024-0416). All procedures adhered to the Guidelines for the Ethical Treatment of Laboratory Animals established by the Ministry of Science and Technology of China.

Note that full information on the approval of the study protocol must also be provided in the manuscript.

Plants

Seed stocks	*Report on the source of all seed stocks or other plant material used. If applicable, state the seed stock centre and catalogue number. If plant specimens were collected from the field, describe the collection location, date and sampling procedures.*
Novel plant genotypes	*Describe the methods by which all novel plant genotypes were produced. This includes those generated by transgenic approaches, gene editing, chemical/radiation-based mutagenesis and hybridization. For transgenic lines, describe the transformation method, the number of independent lines analyzed and the generation upon which experiments were performed. For gene-edited lines, describe the editor used, the endogenous sequence targeted for editing, the targeting guide RNA sequence (if applicable) and how the editor was applied.*
Authentication	*Describe any authentication procedures for each seed stock used or novel genotype generated. Describe any experiments used to assess the effect of a mutation and, where applicable, how potential secondary effects (e.g. second site T-DNA insertions, mosiacism, off-target gene editing) were examined.*
