# Supplementary material for: Cross-species insights into placental evolution and diseases at the single-cell resolution
Source: Nat Commun. 2026 May 9;17:6259. doi: 10.1038/s41467-026-72652-w (PMC13377192; doi:10.1038/s41467-026-72652-w)
Supplement: Supplementary file 2 — Peer Review file [file 41467_2026_72652_MOESM2_ESM.pdf]

nature portfolio

Peer Review File

Cross-species insights into placental evolution and diseases at the single-cell resolution

Corresponding Author: Professor Yu Jiang

This file contains all reviewer reports in order by version, followed by all author rebuttals in order by version.

Version 0:

Reviewer comments:

Reviewer #1

(Remarks to the Author)
The manuscript by Tan et al uses single cell transcriptomics to examine cross-species evolution of the placenta, and to characterize the key cell types present in different placental types. They propose that trophoblast lineages are the main driver of evolution, and that the unique gene expression profile of human trophoblasts contributes to pre-eclampsia and miscarriage. In the later part of the paper they focus on one gene (TGIF1) identified from their analysis as a regulator of trophoblast function.

There is considerable morphological diversity among placentae from different mammalian species, particularly with reference to how maternal and fetal tissues intermingle. However, the molecular bases for these differences are not well understood. This work begins to address the complexity of the maternal-placental interface across 10 species using single-cell transcriptome analyses. While all placental cell types diverge at the gene expression level as species diverge, trophoblasts are the most distinct between species. The authors use their data to attempt to identify the transcription factor network that underlies trophoblast development, and to implicate specific cell types and gene expression patterns in miscarriage in humans. Overall this is an important study that should be of broad interest.

Specific comments:

I may be misinterpreting this, but it does not appear that the cluster numbers (Fig 1C) in the list and the tSNE plot match each other (as just one example, the large Rabbit cluster top right has no number in the main plot). In the legend it states that cells are colored by cell type, but actually appear to be colored by species primarily.

Would separate plots for each cell type across all species be informative? Perhaps UMAP is better for this than tSNE.

I do not see panel 4I on the figure.

It might be more informative to label pathways in the figures with their descriptive name rather than the KEGG pathway number.

The functional analysis of TGIF1 is quite preliminary. This might be an appropriate level of analysis if they were testing multiple genes, but since they only examine one it seems quite minimal. For example, are any of the putative TGIF1 regulome altered on knock-down? What is the TGIF1 protein level on knock-down? They could also examine invasion, and migration using trans-well assays. With a focus on only one gene – some additional follow-up should be provided.

Additionally, a little more discussion or background on TGIF1 would be helpful, since they end the manuscript by focusing on it. How do known TGIF1-regulated pathways fit in with a potential role in trophoblast development? How does this proposed role for TGIF1 fit with reported placental defects in Tgif1 mutant mice?

Reviewer #2

(Remarks to the Author)

The paper by Guanghui Tan et al. presents a comprehensive single-cell transcriptomic atlas of the mammalian placenta, encompassing approximately 300,000 cells from ten species representing the four primary placental types (discoid, cotyledonary, diffuse, and zonary). The analysis identifies trophoblast lineages as key contributors to placental evolution and reveals gene expression dynamics and regulatory networks driving trophoblast development across diverse placental architectures. The study also suggests that the unique gene expression profile of human trophoblasts may contribute to pregnancy complications such as pre-eclampsia and miscarriage, which lacks functional validation and experimental support. shRNA knockdown of TGIF1 as a critical regulator of trophoblast growth and migration in a cell line is disconnected with the discovery and focus of the evolution perspective of the placenta across species. Its link to adverse pregnancy outcomes lacks functional support.

Major points:
1) The whole paper is descriptive and provides suggestive insights. While it provides value to the placental research community, it falls short to functional validation of the discovery. The main focus is the comparative and evolution of the placenta across species. The authors need to validate their discovery, conserved or divergent evolution of the placenta, across species. The shRNA knockout of TGIF1 is disconnected with the whole manuscript with its thin connection to adverse pregnancy. The demonstration is an immortalized cell line provides unphysiological results. Functional validation in primary cells from at least a few different species is needed to support the claims and discoveries.

2) The data is not accessible to review. The provided accession code PRJNA1177647 does not appear to link to any data on NCBI. This is not appropriate for a data analysis paper.

3) Why did the authors decide to include the datasets from Regan L. Scott et al. for rat placenta, given that Scott et al. removed placental tissue and associated decidua, performing scRNA-seq on uterine tissue at the site of trophoblast invasion? In addition, it is reported that the authors sequenced 4 samples for the timepoint E19.5, why the authors used only 3 samples? Which sample was excluded?

4) The authors combined new generated datasets and datasets already published. For the newly generated datasets, they used PISA for the reads alignment and gene counts. For the published datasets they reported:" We curated the published placental cell atlases from Homo sapiens, Cynomolgus macaque, Mus musculus, and Rattus norvegicus." What does this mean? Did the authors re-analyzed the datasets using PISA? If the authors did not reanalyzed the datasets, considering the differences between PISA and Cell Ranger (https://shiquan.github.io/PISA.html#difference-in-alignment-annotation-between-pisa-and-cellranger), could this generate differences between species?

5) For quality control the authors removed cells with less than 20% of mitochondrial content using ATP6, ATP8, COX1, COX2, COX3, CYTB, ND1, ND2, ND3, ND4, ND4L, ND5, ND6 mitochondrial genes. Why did the authors not include the mitochondrial non-coding genes?

6) Why the authors decided to integrate their datasets using CCA? Did they benchmark the other methods using the BENGAL pipeline (https://github.com/Papatheodorou-Group/BENGAL)?

7) In this atlas the authors should show the results after the integration of all the species to clearly demonstrate that all datasets are comparable. The authors only showed Figure 1C and 1D with all the species and cell types, but from this Figure it is not clear if the batch effect was corrected. In addition, it seems like some cell types were removed (ex. Absence of blood cell for rabbit).

Minor points:

1) The authors reported:" FindClusters functions were then employed to identify clusters based on principal components with a P value < 0.01". What does this mean?

2) The authors should include a Supplementary Table reporting the number of cells per datasets before and after QC.

Version 1:

Reviewer comments:

Reviewer #1

(Remarks to the Author)
The revised manuscript by Tan et al is much improved and they have added a significant amount of work addressing TGIF1 function.

The authors have addressed my first four minor points regarding presentation of the bioinformatic data.

While the expanded analysis of TGIF1 function is impressive, there are still a few mostly relatively minor concerns with the data.

Line 839 – should this refer to supplemental Fig 13E or G?

Line 840 – figure 7F-I. This figure does not have panel I.

I think a few extra lines of description of what is being done in panels 7F-H is needed in the main text. (also for supp 13F-J). Otherwise iit is hard to judge how the conclusions are being drawn.

Figure 7H needs quantification. Additionally these images are not very clear. Supplemental 13I looks much better.

Line 881 – describes broad TGIF1 signal in the gene body, referring to Fig 8B. However, Fig 8B (and supplemental 14C) shows a peak around the TSS. More careful description of this panel and the data in Fig 8D is needed.

Line 884 refers to supplemental 14C for motifs, but that is not what it shows.

The motif analysis (Fig 8C) shows very few motifs – for example, among the known motifs three are exactly the same CACGTG motif, and almost all the rest are a version of this. Since the least significant of these is 1e-28, there are likely a lot of other significantly enriched motifs. It might be better to show a full list in the supplement, and version in Fig 8 that includes the best example of each class but goes further down the list. One question that comes up is whether somewhere in the list they find a TGIF1 (or MEIS TALE HD) binding motif (CTGTCA)?

For the analysis of the overlap between TGIF1 peaks and DEGs (Fig 8E,F), it might make more sense to look at gene set enrichment for TGIF1 bound and up-regulated vs TGIF1 bound and down-regulated. Given the known function of TGIF1 as a repressor, the bound and up-regulated (in the knock-down) might include direct targets.

Fig 8G could be supplemental data, allowing more room for a more thorough analysis of the motifs and binding versus changes in expression.

Reviewer #3

(Remarks to the Author)
This manuscript by Tan et al investigates the molecular evolution of the placenta by collecting single cell sequencing datasets from placental tissue from ten different species of mammals representing the four major placental types. The authors identify trophoblasts as harboring the most gene expression variation across species and propose that these cells are the driver of placental diversification. They then used pseudotime analysis, differential gene expression analysis, and pathway analysis to further investigate how trophoblasts contribute to the development of the different major placental types. During these analyses they note that genes and pathways involved in pre-eclampsia are specifically upregulated in human extravillous trophoblasts. The authors then explore the molecular and evolutionary patterns of pre-eclampsia associated genes across their data. Finally, they explore the function of the human gene TGIF1 in trophoblast function and pre-eclampsia. The authors find that TGIF1-binding peaks within the promoter or body regions of multiple genes closely associated with trophoblast cell proliferation, migration, invasion, and pathogenesis of pre-eclampsia and pregnancy loss.

This novel cell atlas and the resulting analyses are an asset to future studies on placental evolution, development, and pre-eclampsia. This study represents a significant body of work that will be of broad interest to a variety of fields.

The authors responded thoroughly to the comments from previous reviewers, which greatly expanded their work. I provide only the following additional comment:

Most of the datasets used were single-nucleus RNA sequencing, while three (Human, Macaca, and Rat) datasets were single-cell RNA sequencing. These two methodologies capture different transcriptomic profiles (single-nucleus only gets nuclear transcripts, while single cell gets nuclear and cytoplasmic transcripts), and single-nucleus RNA sequencing typically captures fewer genes overall. Normalization can’t fix this library complexity and capture efficiency issue, and these biases could affect differential gene expression analyses, which many of the conclusions in this paper are based on. For example, if the human single-cell dataset has more genes per cell captured and more transcripts per gene captured than the single-nucleus dataset being compared to it, then we are more likely to conclude that the human dataset has a gene present or upregulated comparatively that may actually be an artifact. Adding figures to the supplement on the number of genes captured per cell in each sample, and the average number of transcripts captured per gene in each sample, could add context to help understand what impact methodology could be having, and will generally help quantify data quality. Additionally, adding a caveat somewhere in the paper text about this potential effect on data interpretation would help readers who are not as familiar with these technologies and methods better evaluate the conclusions presented.

Open Access This Peer Review File is licensed under a Creative Commons Attribution 4.0 International License, which permits use, sharing, adaptation, distribution and reproduction in any medium or format, as long as you give appropriate credit to the original author(s) and the source, provide a link to the Creative Commons license, and indicate if changes were made.
In cases where reviewers are anonymous, credit should be given to 'Anonymous Referee' and the source.
The images or other third party material in this Peer Review File are included in the article's Creative Commons license, unless indicated otherwise in a credit line to the material. If material is not included in the article's Creative Commons license and your intended use is not permitted by statutory regulation or exceeds the permitted use, you will need to obtain permission directly from the copyright holder.
To view a copy of this license, visit https://creativecommons.org/licenses/by/4.0/

Reviewer #1

The manuscript by Tan et al uses single cell transcriptomics to examine cross-species evolution of the placenta, and to characterize the key cell types present in different placental types. They propose that trophoblast lineages are the main driver of evolution, and that the unique gene expression profile of human trophoblasts contributes to pre-eclampsia and miscarriage. In the later part of the paper they focus on one gene (TGIF1) identified from their analysis as a regulator of trophoblast function.

There is considerable morphological diversity among placentae from different mammalian species, particularly with reference to how maternal and fetal tissues intermingle. However, the molecular bases for these differences are not well understood. This work begins to address the complexity of the maternal-placental interface across 10 species using single-cell transcriptome analyses. While all placental cell types diverge at the gene expression level as species diverge, trophoblasts are the most distinct between species. The authors use their data to attempt to identify the transcription factor network that underlies trophoblast development, and to implicate specific cell types and gene expression patterns in miscarriage in humans. Overall, this is an important study that should be of broad interest.

Specific comments:

1). I may be misinterpreting this, but it does not appear that the cluster numbers (Fig 1C) in the list and the tSNE plot match each other (as just one example, the large Rabbit cluster top right has no number in the main plot). In the legend it states that cells are colored by cell type, but actually appear to be colored by species primarily.

Response: To improve clarity, we have integrated the UMAP clustering results for each species into the main Fig. 1C, and in Fig. 2A we present the cross-species integrated embedding obtained after mapping expression to 1:1 orthologs. The figure legend and Methods have been updated accordingly. The current revisions are shown in the figure below.

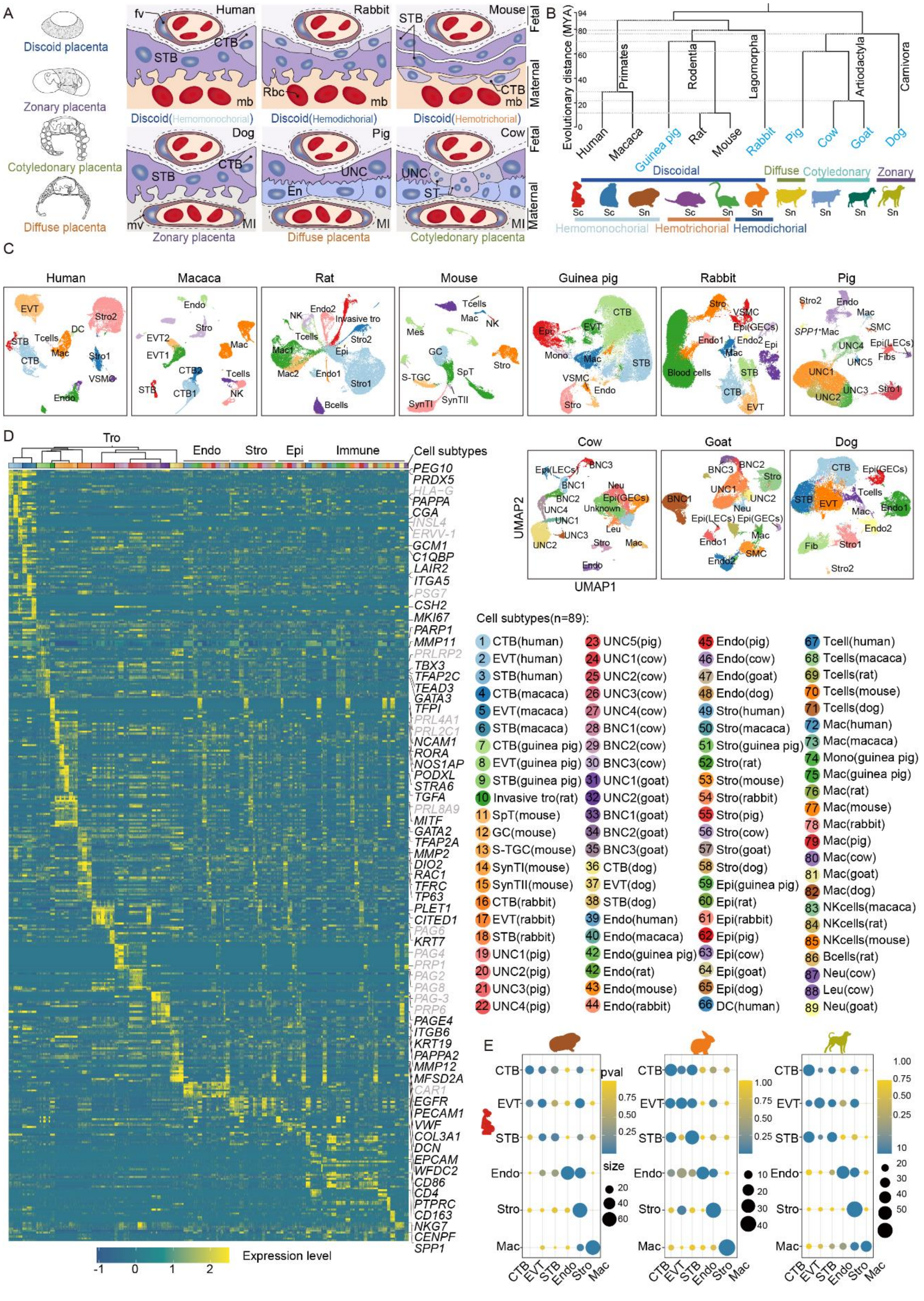

Fig.1: Single-cell / -nucleus transcriptomic mapping and cell type analysis of the mammalian placentas.

(A and B) Left: Schematic representation of different types of placentas. The structures and maternal-fetal

interfaces of discoid, zonary, cotyledonary, and diffuse placentas are shown. FB, fetal blood; fv, fetal vessel; MI. maternal interstitium: mv, maternal vessel; ST, specific trophoblast； MB, maternal blood; En, endometrium; Rbc, red blood cell. Right: Phylogeny of the ten mammalian species was analyzed in this work. The scale bar indicates the estimated divergence time (generated via https://phylot.biobyte.de/). MYA, million years ago. Species names in blue indicate data generated in this study, while species names in black represent those reported by previous studies. "Sc" indicates scRNA-seq, and "Sn" represents snRNA-seq. (C) UMAP plots showing clustering results of single-cell or single-nucleus transcriptomes from the placenta or maternal-fetal interface of ten mammalian species: humans[1], macacas[2], rats[3], mice[4], guinea pigs, rabbits, pigs, cows, goats, and dogs. Each color represents a distinct cell population, including cytotrophoblasts (CTBs), extravillous trophoblasts (EVTs), sncytiotrophoblasts (STB), uninucleate cells (UNCs), binucleate cells (BNCs), stromal cells (Stro, including stromal, fibroblast, and smooth muscle cell subtypes), endothelial cells (Endo), macrophages (Mac), epithelial cells (e.g., Epi, Epi / LEC / GECs comprising luminal and glandular epithelial cells), dendritic (DC), and invasive trophoblasts (Invasive tro). The UMAP plots for biological replicates of each species are shown in Supplementary Figs.1B-K. (D) A heatmap showing the scaled expression levels of cell type-specific markers (left), with each cell type downsampled to 200 cells / nucleus. Columns are color-coded to represent different cell subtypes. Blood cell types were excluded from the analysis, and glandular epithelial cells (GECs) and luminal epithelial cells (LECs) were merged into a single category labeled "Epi". In addition, certain subclusters were combined into broader categories for clarity, such as Endo1 and Endo2 merged into "Endo", and Stro1 and Stro2 merged into "Stro". Expression patterns of 89 representative markers are shown on the right, with more detailed information on marker expression for each cell type provided in Supplementary Figs.1F-K and Supplementary Data 3. Grey genes indicate the missing data owing to the absence of the corresponding orthologue in the species annotation. (E) Dot plots showing the overlap between cell markers for trophoblasts (Tro), stromal cells, as well as endothelial cells reported by previous human scRNA-seq studies and the cluster markers used in the present study. The size of the dots corresponds to the overlap between the cluster gene sets in this study and the gene sets in previous human studies, whereas the color of the dot represents the adjusted *P* value of the one-sided overrepresentation Fisher's exact test.

2). Would separate plots for each cell type across all species be informative? Perhaps UMAP is better for this than tSNE.

Response: We have incorporated the UMAP clustering panels for each species into the main Fig. 1C. In addition, Fig. 2A presents the cross-species integrated embedding obtained after mapping gene expression to the space of 1:1 orthologs, enabling more intuitive comparisons of cell types across species.

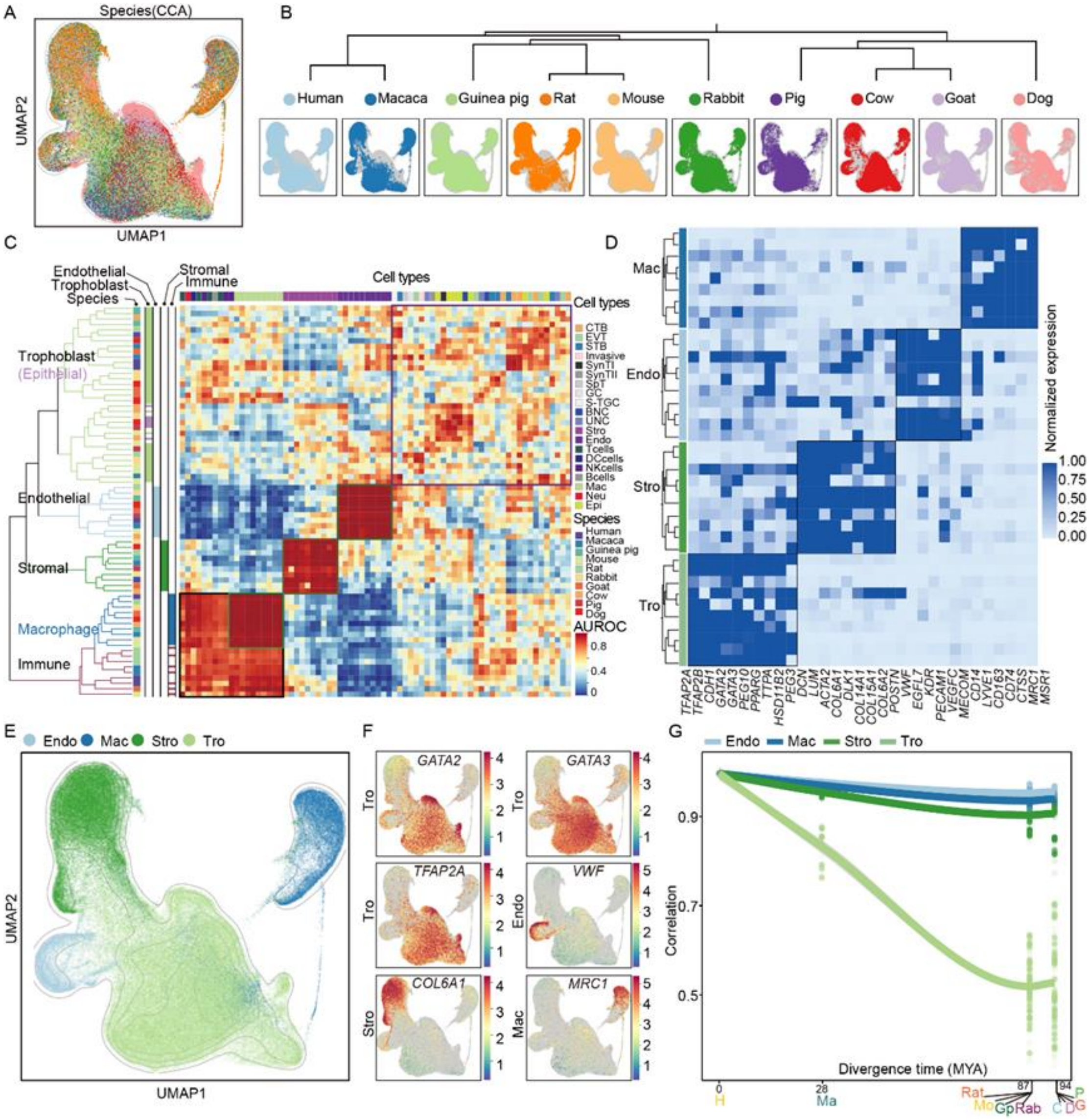

Fig.2: Conserved and divergent cell types during placental evolution

3). I do not see panel 4I on the figure.

Response: The labeling error has been rectified: panel Fig. 4I has been restored and correctly labeled (the panel previously mislabeled as Fig. 4J has now been corrected to 4I). The revised Fig. 4 is displayed below. (lines 593 – 595).

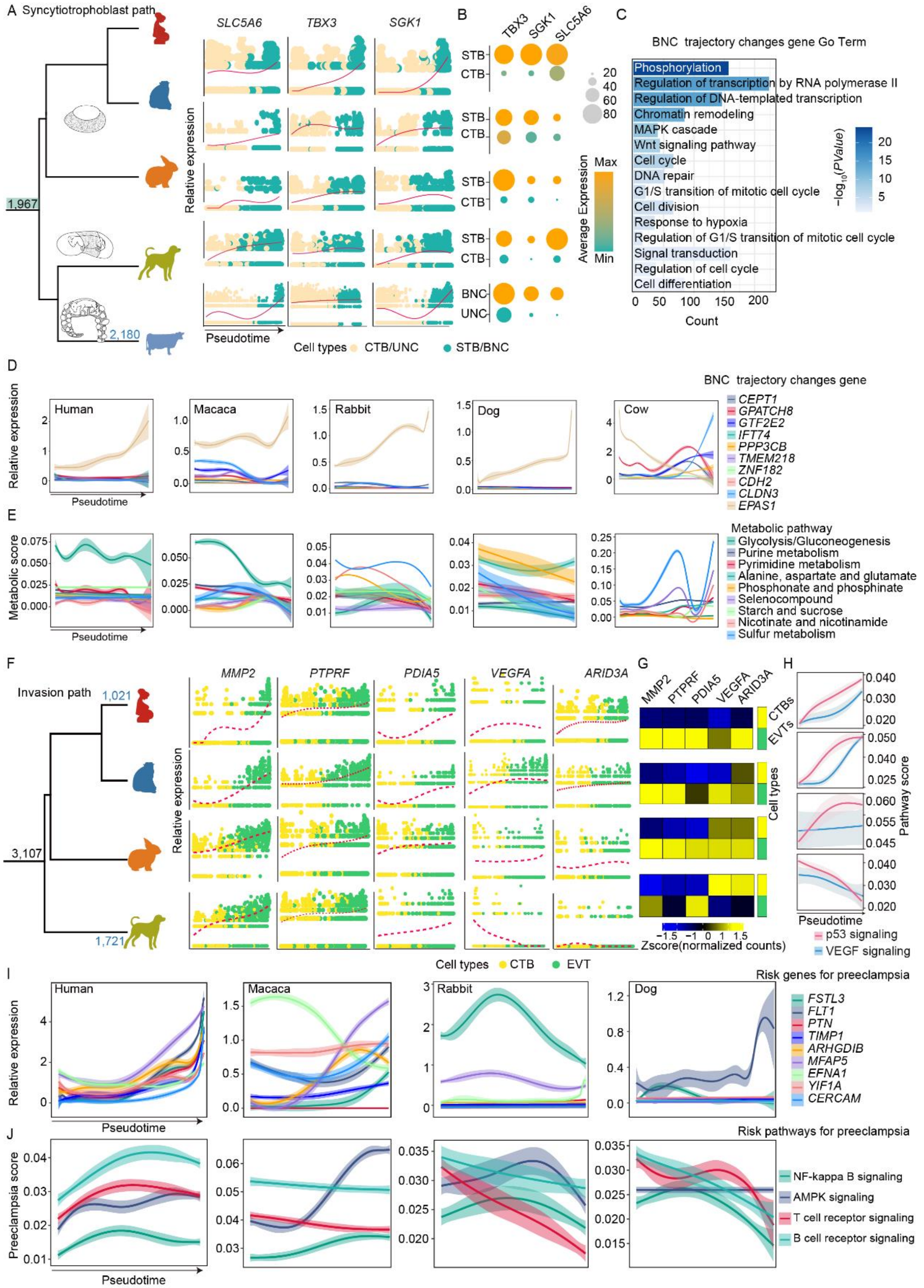

Fig.4: Conserved and divergent gene expression along trophoblast differentiation. (A) Evolutionary relationships among species and the number of conserved (black) and divergent (blue) gene expression trajectories in the fusion pathway. Branch lengths of the evolutionary tree have no biological meaning. The species from the top to the bottom are humans, macacas, rabbits, dogs, and cows. The middle section shows

the expression changes of conserved and divergent genes along pseudotime during trophoblast fusion, with each row corresponding to a species and colors representing different cell types. (B) The dot plot showing the average expression levels of genes across different cell types (STB, CTBs, BNCs, and UNCs) in various species. (C) The bar chart showing the GO enrichment result of trajectory-changing genes in cows, with all enrichment results detailed in Supplementary Data 5. (D) The expression patterns of the exemplary genes along pseudotime in the trophoblast fusion pathways in different species (humans, macacas, rabbits, dogs, and cows). The expression trend in cows differs from that in other species. All related genes are listed in Supplementary Data 5. (E) Examples of the activity scores (AUCell) of conserved and divergent metabolic pathways during trophoblast fusion in cows. Detailed descriptions of metabolic pathways are provided in Supplementary Data 5, and activity scores for all pathways are shown in Supplementary Figs.8C-G. Of these, glycolysis / gluconeogenesis, phosphonate and phosphinate metabolism, and aminoacyl-tRNA biosynthesis were conserved metabolic pathways, while the others were pathways with increased specificity in cows. (F) Evolutionary relationships among species and the number of conserved (black) and divergent (blue) gene expression trajectories in the EVT pathway. The species from the top to the bottom are humans, macacas, rabbits, and dogs. The middle section shows the expression changes of conserved and divergent genes during pseudotime EVT differentiation, with each row corresponding to a species and colors representing different cell types. In the evolutionary tree, the black number (3,107) represents the number of genes with conserved expression patterns across all species in relation to humans (the alignment similarity ≥ 60%), while the blue numbers (1,721 and 1,021) represent the numbers of genes with trajectory changes in the EVT pathway (the alignment similarity ≤ 40%). All related genes are listed in Supplementary Data 5. (G) The heatmap showing the average gene expression levels across different cell types in various species. (H) The pathway scores of VEGF signaling and the p53 signaling pathway during EVT differentiation. The order of species from top to bottom is consistent with the evolutionary tree order in Fig.4F. (I and J) Examples of activity scores for reported PE-related genes (I) and pathways (J) during EVT differentiation across different species. Pathway activity scores for EVT differentiation across all species are shown in Supplementary Data 6.

4). It might be more informative to label pathways in the figures with their descriptive name rather than the KEGG pathway number.

Response: We agree with you and have revised Fig. 4 by replacing the KEGG pathway numbers with their corresponding descriptive pathway names.

5). The functional analysis of TGIF1 is quite preliminary. This might be an appropriate level of analysis if they were testing multiple genes, but since they only examine one it seems quite minimal. For example, are any of the putative TGIF1 regulome altered on knock-down? What is the TGIF1 protein level on knock-down? They could also examine invasion, and migration using trans-well assays. With a focus on only one gene – some additional follow-up should be provided.

Response: To address the reviewer's concern, we made the following changes:To further

elucidate human-specific gene regulatory networks, we applied GENIE3 (GeNeralized Influential Network Inference Engine), a machine learning-based network inference algorithm[124], to the relevant single-cell transcriptomic data. The analysis uncovered several key transcription factors (TFs) that predominantly influenced the expression of human-biased genes, among them well-known trophoblast TFs such as *GATA2/3*, *CEBPB*, *ATF4*, and *FOS* (Fig. 7B). These TFs have been frequently reported to be closely linked with pregnancy-related diseases, including preeclampsia (PE) and pregnancy loss [references 125, 126, 127, 128], aligning with the findings from the GWAS association analysis (Supplementary Figs. 13A-C). Notably, *TGIF1*, which was ranked among the top six TFs in this study, garnered our attention due to the lack of systematic exploration into its role in human or mouse placental trophoblast development. Upon analyzing the transcriptomic data, we discovered that the *TGIF1* target genes in human extravillous trophoblasts (EVTs) encompassed not only genes closely associated with pregnancy-related diseases such as PE and pregnancy loss but also key genes involved in trophoblast development and invasive functions (Fig. 7C, Supplementary Data 10). Furthermore, *TGIF1* expression was found to be significantly higher in placentas from PE patients (Supplementary Fig. 13D).

Based on these findings, we conducted an immunofluorescence analysis on human term placental tissue (gestational week 40) and identified TGIF1 staining in the nuclei of HLA-G$^+$ EVTs (Fig. 7D), suggesting its potential role in EVT function (Fig. 7D). Subsequently, we knocked down *TGIF1* in human trophoblast stem cells (hTSCs) and the HTR-8/SVneo cell line (a human chorionic trophoblast cell line[129]) by introducing a *TGIF1*-shRNA expression vector into the cells via lentiviral transduction ((Fig. 7E, Supplementary Fig. 13E). We observed markedly reduced trophoblast proliferation, invasion, migration, and clonogenic ability upon *TGIF1* downregulation (Figs. 7F-I and Supplementary Figs. 13F-J). In summary, our results indicate that *TGIF1* contributes to the expression of pregnancy disorder-associated genes and regulates invasive trophoblast function, offering insights into the molecular basis of conditions such as PE and pregnancy loss.

TGIF1 target binding to trophoblast functional genes associated with PE

TFs exert their guiding role mostly by recognizing target DNA sequences to modulate chromatin and transcription. To delve into the mechanism by which TGIF1 regulates trophoblast cell proliferation and migration, an RNA-seq analysis was performed on biological triplicates of control and shTGIF1 hTSCs (Supplementary Data 10). A total of 6,486 differentially expressed genes (DEGs, $|Log_2Fold\ Change| \geq 1$, $P_{adj.} \leq 0.05$) were identified, with the majority being downregulated in the shTGIF1 group (Fig. 8A). GO and KEGG enrichment analyses revealed that these downregulated genes were significantly enriched in several key

pathways, including those related to cell proliferation (e.g., MAPK and PI3K-Akt signaling pathways), cell migration and invasion (e.g., ECM-receptor interaction and cell adhesion molecules [CAMs]), as well as immune response and cellular senescence (e.g., response to type I interferon, IL-17 signaling pathway, p53 signaling pathway, and cellular senescence) (Supplementary Figs.14 A and B). These findings are highly consistent with our phenotypic observations that *TGIF1* knockdown impaired trophoblast cell proliferation, migration, and invasion.

Next, a CUT&Tag assay for TGIF1 was conducted on biological duplicates of hTSCs, with the aim of identifying the TGIF1 DNA-binding sites in the human genome. Overall, 3,528 to 4,012 peaks were identified, with an average width of 474.79 bp and an enrichment score of 5.18, corresponding to 3,108 to 3,481 annotated genes. In contrast, only 49 peaks were detected in the IgG control samples, indicating high binding specificity (Supplementary Data 10). Density plots of TGIF1 DNA-binding sites revealed a wide range of binding signals in gene body regions (Fig. 8b). The motif analysis of TGIF1 CUT&Tag peaks showed significant enrichment of multiple known and newly identified transcription factor (TF) binding motifs (Fig. 8c, Supplementary Figs. 14C). A genome distribution analysis revealed that these peaks were highly enriched in intergenic regions, accounting for 35.50% of all peaks. Notably, 9.89% of peaks were located in putative promoter regions 3 kb upstream of transcription start sites (TSS, Fig. 8D). Among the genes directly bound by TGIF1, 986 were differentially expressed between control and shTGIF1 groups, of which 430 and 556 genes were upregulated and downregulated, respectively (Fig. 8E). The GO analysis indicated that these TGIF1 target genes were enriched with biological processes related to metal ion transport, cell-cell adhesion, regulation of GTPase activity, regulation of cell growth, and regulation of cell migration (Fig. 8F).

Specifically, the Integrative Genomics Viewer (IGV) revealed TGIF1-binding peaks within the promoter or body regions of multiple genes closely associated with trophoblast cell proliferation, migration, invasion, and the pathogenesis of preeclampsia (PE) and pregnancy loss (Fig. 8G, Supplementary Figs. 14C). For example, *ADAM12* is a candidate regulator of trophoblast invasion and a first-trimester marker of healthy trophoblasts, with its expression consistently linked to the progression of PE and fetal growth restriction (FGR)[130]. *IGF2* plays crucial roles in placental development, promoting trophoblast proliferation, extravillous trophoblast (EVT) migration, hormone secretion, glucose and amino acid uptake, and reducing apoptosis[131]. *COL4A2*[132], *KISS1*[133], *SHH*[134], *WNT3A*[135], *RGL3*[136], and *ZNF831*[136] have been identified as risk-associated genes for human PE and recurrent miscarriage. Additionally, *GAS6* signaling can induce PE, and inhibition of *AXL* prevents disease progression in pregnant rats[137]. *PIK3R6*, *AXL*, and *WNT7B* are involved in the regulation of the PI3K, TAM, and WNT

signaling pathways, respectively, which are essential for trophoblast invasion and angiogenesis and strongly associated with PE[138, 139]. *PIK3R6* and *TFDP1* are also implicated in cell cycle regulation, potentially affecting trophoblast proliferation dynamics. Furthermore, several additional PE-related candidate genes, including *ITGB2*, *CTSL*, *EGFR*, and *DLX3*, also exhibited clear TGIF1 binding signals (Supplementary Figs. 14E). These findings suggest that *TGIF1* may contribute to the regulation of trophoblast function and PE pathogenesis through its interaction with *cis*-regulatory elements of a set of functionally critical genes.

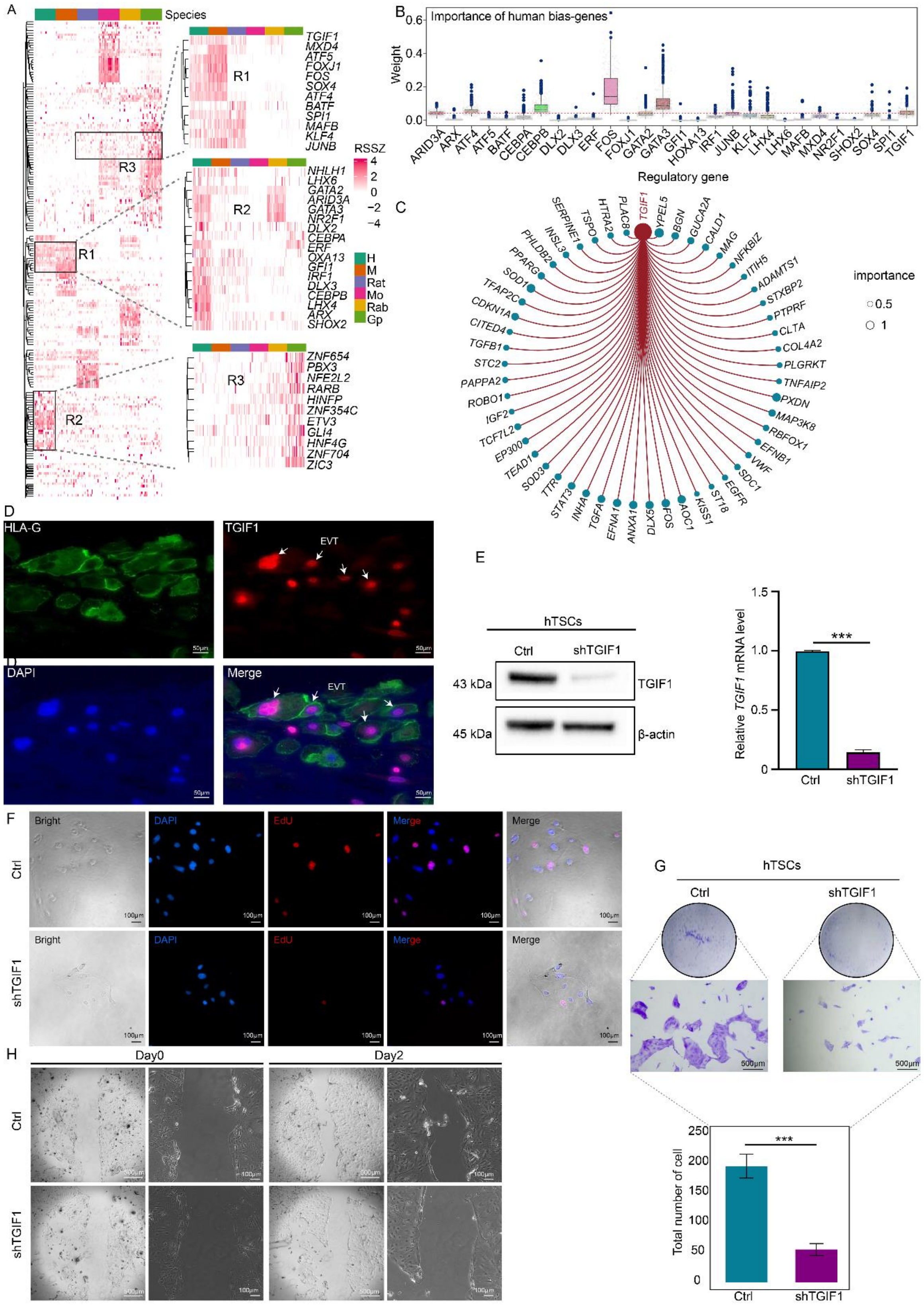

Fig.7: Key regulators of invasive trophoblasts and their potential roles in pregnancy disorders. (A) The heatmap displaying the activity of regulatory factors in invasive trophoblasts across six species. The regulatory

factors are categorized into different regions (R1, R2, and R3) based on conservation, with black boxes highlighting the clusters of highly specific regulatory factors in primates and rodents. (B) The correlation between human-biased genes (avg_$log_2FC \geq 1$) and human-specific regulatory factors. (C) The regulatory network showing predicted *TGIF1* target genes in human EVTs. (D) The immunofluorescence analysis of human placental tissue (gestational week 40) showing TGIF1 staining (red) in the nuclei of HLA-G^+ EVTs (green). Nuclei were counterstained with DAPI (blue). Arrows indicate EVTs co-stained with TGIF1 and HLA-G. (E) TGIF1 knockdown in hTSCs confirmed by Western blotting (left) and qRT-PCR (right). β-actin was used as a loading control. (F) The EdU incorporation assay. Red, blue, and magenta indicate the EdU^+ cells, $DAPI^+$ nuclei, and the merge. (G) The transwell invasion assay showing the impaired invasive ability of TGIF1-deficient hTSCs. Representative images (top) and quantification of invaded cells (bottom) are shown. (H) The migration assay showing the reduced migratory capacity in shTGIF1 hTSCs on days 0 and 2 post-scratch. Scale bars are indicated. Data are presented as the mean ± SEM of biological triplicates (n = 3). ***: $P < 0.001$.

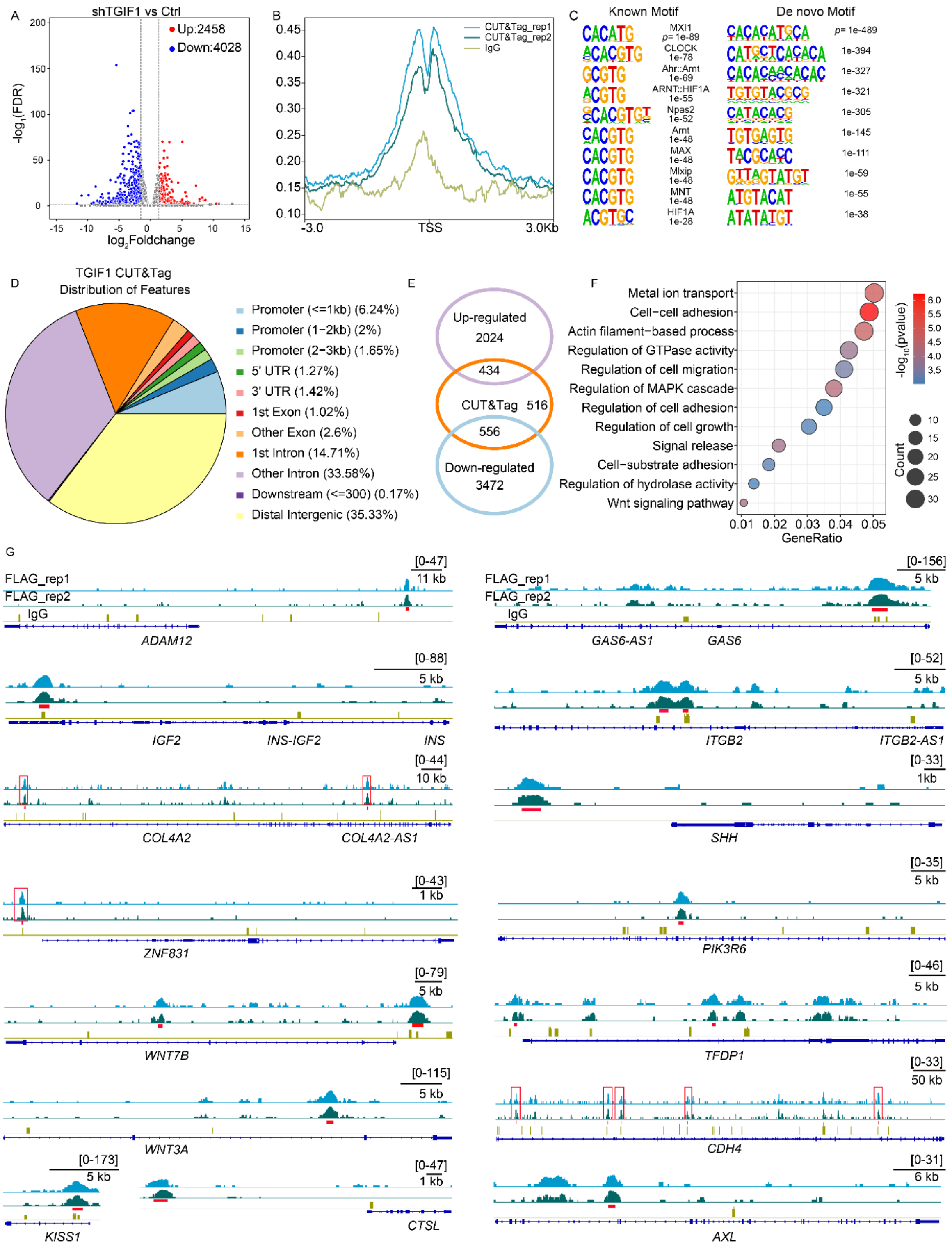

Fig.8: Genome-wide identification of the binding motifs of TGIF1. (A) The volcano plot of DEGs (shTGIF1 vs. control). (B) Distribution of TGIF1 CUT&Tag signals across transcription start sites (TSS) ($\pm$3 kb), showing promoter enrichment. (C) The DNA motif analysis of TGIF1 binding sites. Known motifs: previously reported motifs; *De novo* motifs: newly predicted motifs. (D) The genomic distribution profile of TGIF1 CUT&Tag peaks.

(E) The Venn diagram showing the overlap between genes associated with TGIF1-binding sites and the DEGs identified by RNA-seq. (F) GO terms for the genes associated with TGIF1-binding sites. (G) Chromatin binding peaks of TGIF1 at genomic regions associated with pregnancy disorder-related genes.

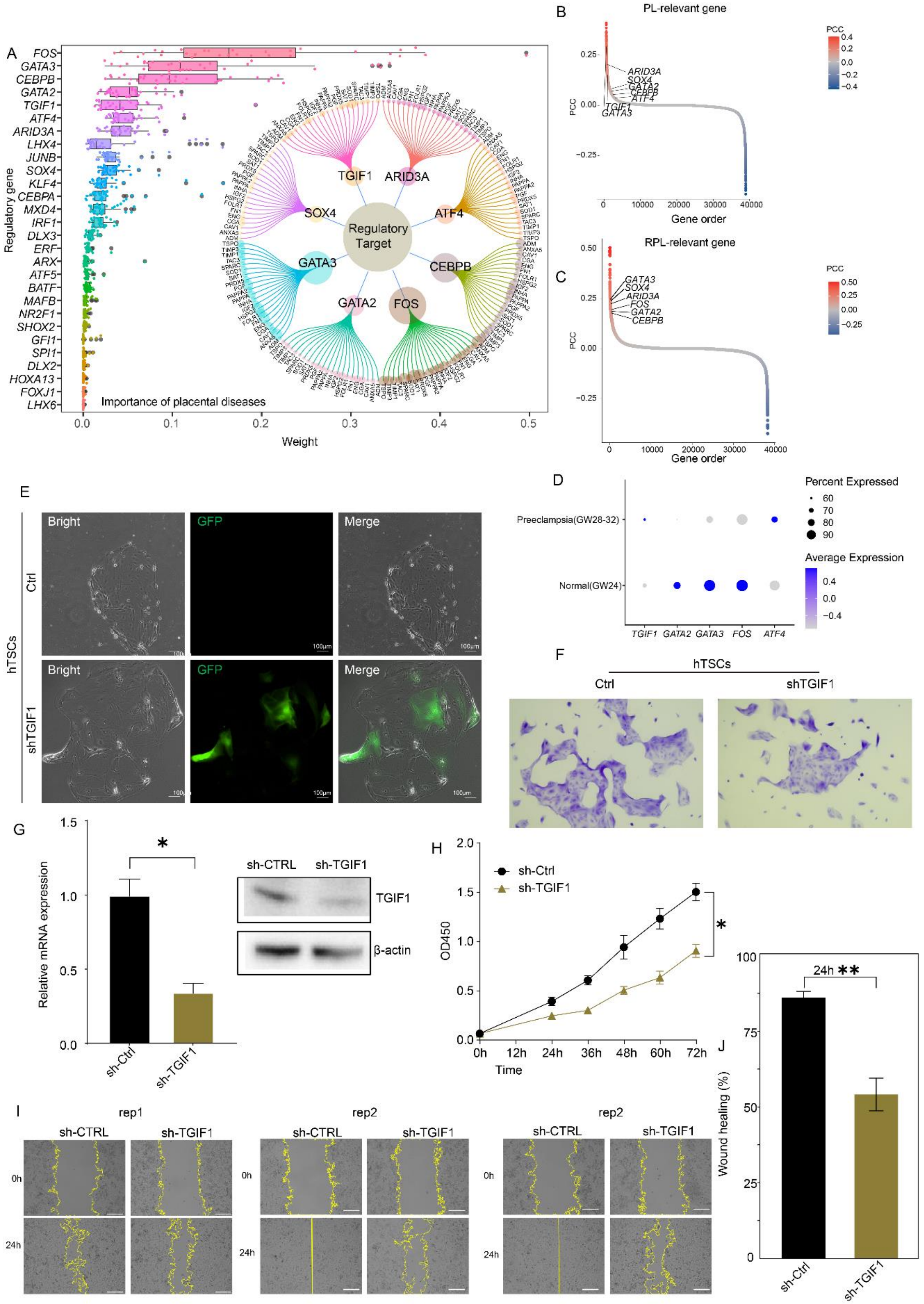

Supplementary Figs.13: The correlation between identified TFs and (recurrent) pregnancy loss.

(A) The analysis of different regulatory factors, revealing that the gene regulatory network demonstrates close

interactions between key TFs such as *TGIF1*, *FOS*, and *GATA3* and various high-risk genes associated with pregnancy-related diseases. (B and C) The ranking chart showing the correlation between several identified TFs and (recurrent) pregnancy loss in GWAS results. TFs of interest, such as *GATA3*, *SOX4*, *ARID3A*, *CEBPB*, and *FOS*, are annotated. (D) Gene expression in EVTs from early PE placentas and their normal counterparts. (E) Images of the transduced hTSCs. Bar = 100 μm. (F) The clonogenic assay for the control and shTGIF1 hTSCs. (G-I) Experiments were performed on HTR-8/SVneo cells to evaluate the effects of *TGIF1* knockdown. G, TGIF1 expression in the scramble control (sh-Ctrl) and *TGIF1*-knockdown (sh-*TGIF1*) groups was measured by qPCR and Western blot analyses. (H) A CCK-8 assay showing that knockdown of *TGIF1* in the HTR-8/SVneo cells significantly reduced cell proliferation after 72 h. (I) Representative images of the migration assay at 0 and 24 h after scratching the cell monolayer. The yellow lines indicate the boundary of the wound area. The left and right columns show cells transduced with the scramble control (sh-Ctrl) and *TGIF1*-target shRNA (sh-*TGIF1*), respectively. Bar = 100 μm. (J) The percentage of wound healing, revealing quantification of wound closure after 24 h. Data are presented as the mean ± SEM of biological triplicates (n = 3). *: $P < 0.05$; **: $P < 0.01$.

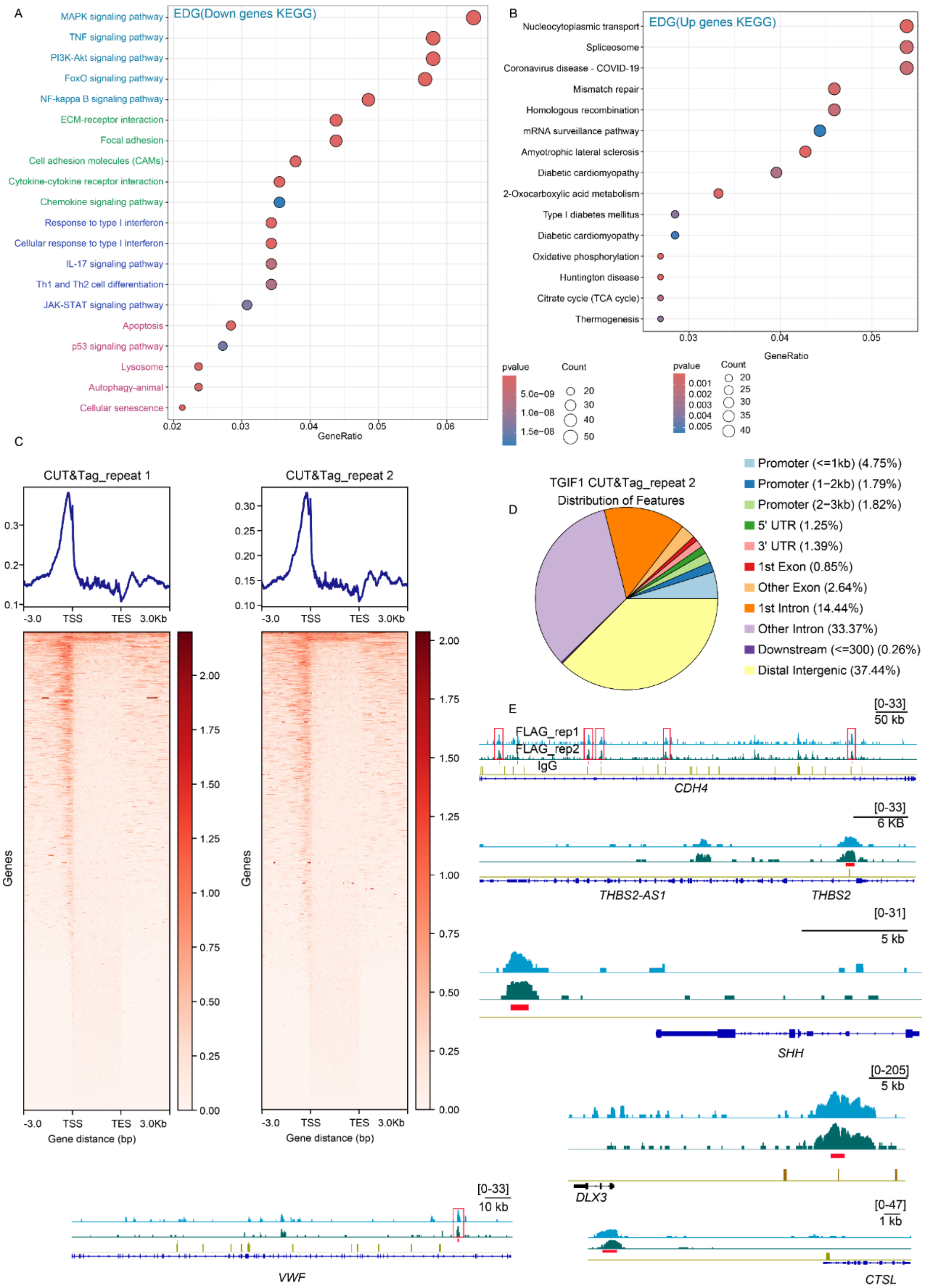

A
EDG(Down genes KEGG)
MAPK signaling pathway
TNF signaling pathway
PI3K-Akt signaling pathway
FoxO signaling pathway
NF-kappa B signaling pathway
ECM-receptor interaction
Focal adhesion
Cell adhesion molecules (CAMs)
Cytokine-cytokine receptor interaction
Chemokine signaling pathway
Response to type I interferon
Cellular response to type I interferon
IL-17 signaling pathway
Th1 and Th2 cell differentiation
JAK-STAT signaling pathway
Apoptosis
p53 signaling pathway
Lysosome
Autophagy-animal
Cellular senescence
GeneRatio
pvalue
5.0e-09
1.0e-08
1.5e-08
Count
B
EDG(Up genes KEGG)
Nucleocytoplasmic transport
Spliceosome
Coronavirus disease - COVID-19
Mismatch repair
Homologous recombination
mRNA surveillance pathway
Amyotrophic lateral sclerosis
Diabetic cardiomyopathy
2-Oxocarboxylic acid metabolism
Type I diabetes mellitus
Diabetic cardiomyopathy
Oxidative phosphorylation
Huntington disease
Citrate cycle (TCA cycle)
Thermogenesis
GeneRatio
pvalue
Count
C
CUT&Tag_repeat 1
CUT&Tag_repeat 2
TSS
TES
Genes
Gene distance (bp)
D
TGIF1 CUT&Tag_repeat 2
Distribution of Features
Promoter (<=1kb) (4.75%)
Promoter (1−2kb) (1.79%)
Promoter (2−3kb) (1.82%)
5' UTR (1.25%)
3' UTR (1.39%)
1st Exon (0.85%)
Other Exon (2.64%)
1st Intron (14.44%)
Other Intron (33.37%)
Downstream (<=300) (0.26%)
Distal Intergenic (37.44%)
E
FLAG_rep1
FLAG_rep2
IgG
[0-33]
50 kb
CDH4
[0-33]
6 KB
THBS2-AS1
THBS2
[0-31]
5 kb
SHH
[0-205]
5 kb
DLX3
[0-47]
1 kb
CTSL
[0-33]
10 kb
VWF

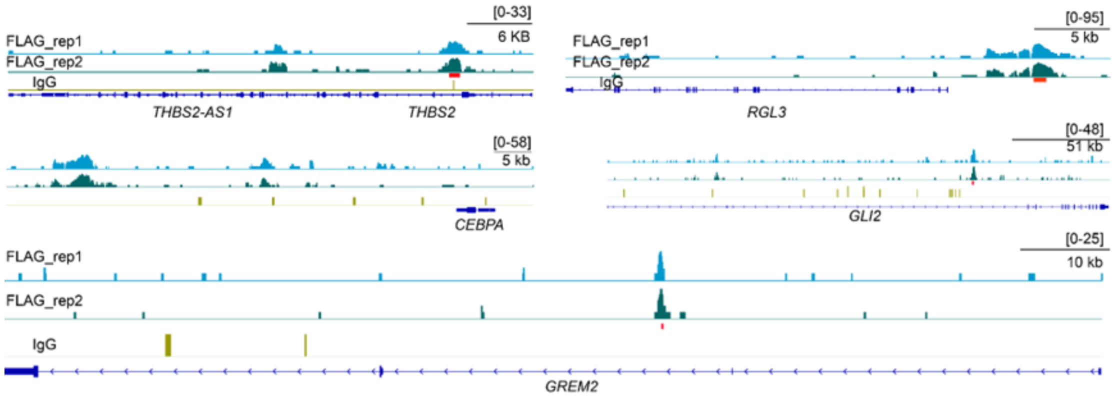

Supplementary Figs.14: Genome-wide identification of the binding motifs of TGIF1.
(A-B) GO and KEGG enrichment analyses of DEGs. (C) Heatmaps and average profiles of TGIF1 CUT&Tag signal intensity near transcription start sites (TSS) and transcription end sites (TES) in biological duplicates (repeat 1 and repeat 2). (D) Genomic distribution of TGIF1 CUT&Tag peaks based on feature annotations. (E) Genome browser tracks showing representative TGIF1 binding peaks.

6) Additionally, a little more discussion or background on TGIF1 would be helpful, since they end the manuscript by focusing on it. How do known TGIF1-regulated pathways fit in with a potential role in trophoblast development? How does this proposed role for TGIF1 fit with reported placental defects in Tgif1 mutant mice?

Response: We fully agree with you that providing additional background and discussion on *TGIF1* would enhance the focus on this gene in the latter part of the manuscript. Some background on *TGIF1*-regulated pathways is as follows:

TGIF1, also known as transforming growth factor β-induced factor or TG interacting factor, is a member of the TALE (three-amino-acid loop extension) subfamily of homeobox genes. The clinical relevance of *TGIF1* has been demonstrated in holoprosencephaly, a congenital malformation of the developing human forebrain[5] and more recently, in myelogenous leukaemia, where *TGIF1* expression has been shown to correlate inversely with patient survival[6, 7]. The biological significance of *TGIF1* has been highlighted in murine placental development using knockout models. Specifically, targeted deletion of *Tgif1* in the mice led to decreased labyrinth vascularity and size as well as decreased expression of the gap-junction protein Connexin 26, resulting in placental insufficiency and severely growth-restricted embryos[8]. In human idiopathic fetal growth restriction (FGR) placentas, TGIF1 mRNA and protein levels are significantly increased compared to those in controls. In BeWo trophoblast cells, siRNA-

mediated knockdown of *TGIF1* resulted in significant downregulation of trophoblast differentiation markers at both mRNA and protein levels[9]. These findings support the notion that *TGIF1* functions as a potential upstream regulator of trophoblast differentiation, and that altered *TGIF1* expression may contribute to abnormal villous trophoblast development (lines 1073 – 1085).

Based on these reports, we systematically investigated the role of *TGIF1* in human trophoblasts using molecular biology approaches. This included stable *TGIF1* knockdown in human trophoblast stem cells (hTSCs) and HTR-8/SVneo cells, followed by RNA-seq to assess global transcriptomic changes and CUT&Tag to identify genome-wide TGIF1-binding sites. Functional assays demonstrated that *TGIF1* knockdown significantly impaired trophoblast proliferation, invasion, migration, and clonogenic ability. The integrative analysis further revealed that TGIF1 directly regulates a set of genes associated with preeclampsia (PE) and pregnancy loss, many of which are functionally linked to the relevant cellular processes. These findings not only validate the significance of *TGIF1* in human placental biology but also provide novel mechanistic insights into how its dysregulation may contribute to the pathogenesis of pregnancy complications (refer to Fig7 and Fig8). This content has been incorporated into the Discussion section of the revised manuscript (lines 1073 – 1085).

Reviewer #2

The paper by Guanghui Tan et al. presents a comprehensive single-cell transcriptomic atlas of the mammalian placenta, encompassing approximately 300,000 cells from ten species representing the four primary placental types (discoid, cotyledonary, diffuse, and zonary). The analysis identifies trophoblast lineages as key contributors to placental evolution and reveals gene expression dynamics and regulatory networks driving trophoblast development across diverse placental architectures. The study also suggests that the unique gene expression profile of human trophoblasts may contribute to pregnancy complications such as pre-eclampsia and miscarriage, which lacks functional validation and experimental support. shRNA knockdown of TGIF1 as a critical regulator of trophoblast growth and migration in a cell line is disconnected with the discovery and focus of the evolution perspective of the placenta across species. Its link to adverse

pregnancy outcomes lacks functional support.

Response: We thank you for this insightful comment. By analyzing invasive trophoblast cells across species with discoid placentation, we observed that human-biased genes were significantly associated with pregnancy-related disorders (Fig. 5E-F). Further integration of cross-species gene regulatory networks revealed *TGIF1* as a candidate transcriptional regulator specifically expressed in human trophoblasts. Network analysis between human-biased genes and upstream regulators highlighted *TGIF1* as one of the top five key regulators, suggesting its potential role in mediating species-specific trophoblast functions in humans. In this sense, functional validation of *TGIF1* in HTR-8/SVneo cells is not disconnected from the discovery but rather an important and direct expansion of our evolutionary analysis. It was based on systematic prioritization from cross-species comparisons, with the aim to validate a key evolutionary finding rather than shifting the focus away from the evolutionary context. To clarify this point, we have added a transitional paragraph to the revised manuscript between the evolutionary and functional sections, explicitly explaining how *TGIF1* was selected and how the functional work built on the evolutionary discovery (lines 823 – 831).

Furthermore, in this revised version, we have conducted a series of experiments, including the knockdown of TGIF1 in human trophoblast stem cells (hTSCs) and HTR-8/SVneo cells, followed by functional assays such as EdU incorporation, transwell invasion, migration, and clonogenic assays to assess its role in trophoblast proliferation, invasion, migration, and clonogenic ability. To elucidate the underlying molecular mechanisms, we further conducted RNA-seq and CUT&Tag analyses to identify the direct TGIF1 targets associated with pregnancy disorders. The overall data validate the significance of *TGIF1* in human placental biology and provide novel mechanistic insights into how its dysregulation may contribute to the pathogenesis of pregnancy complications.

Major points:

1) The whole paper is descriptive and provides suggestive insights. While it provides value to the placental research community, it falls short to functional validation of the discovery. The main focus is the comparative and evolution of the placenta across species.

The authors need to validate their discovery, conserved or divergent evolution of the placenta, across species. The shRNA knockout of TGIF1 is disconnected with the whole manuscript with its thin connection to adverse pregnancy. The demonstration is an immortalized cell line provides unphysiological results. Functional validation in primary cells from at least a few different species is needed to support the claims and discoveries.

Response: We agree, and additional functional validation has been included as follows:

First, *TGIF1* was identified as a candidate regulator through cross-species trophoblast transcriptional network analysis, and it ranked among the top five regulators in terms of predicted regulatory influence. Therefore, functional validation of *TGIF1* is not disconnected from the principal results but rather a direct extension of our evolutionary findings. To enhance the structure and logic of this manuscript, we have added a transitional paragraph between the evolutionary analysis and the functional validation section (lines 823 – 831), explicitly clarifying how *TGIF1* was selected through cross-species screening and why it was chosen for downstream functional studies.

Second, we concur with your assessment that functional validation in HTR-8/SVneo cells, an immortalized cell line, may yield less physiologically relevant results. To enhance clinical relevance and ensure robust functional validation, we have conducted additional experiments using primary human trophoblast stem cells (hTSCs, a widely accepted primary-derived[10, 11, 12] source), to thoroughly investigate the expression and functional roles of *TGIF1* in human trophoblast cells.

(1) Expression validation: We conducted immunofluorescence staining on term human placental tissue (gestational week 40) and confirmed that TGIF1 is expressed in the nuclei of HLA-G^+ extravillous trophoblasts (EVTs) (Fig. 7D), suggesting its potential role in EVT functions.

(2) Knockdown validation: We knocked down *TGIF1* in both human trophoblast stem cells (hTSCs) and the HTR-8/SVneo cell line (a human chorionic trophoblast cell line) by introducing a *TGIF1*-shRNA expression vector into the cells via lentiviral transduction. Q-PCR and Western blotting further verified efficient knockdown at both mRNA and protein levels (Fig. 7E, Supplementary Fig. 13D).

(3) Functional assays: We conducted EdU incorporation, transwell invasion, migration, and

clonogenic assays on both cell populations, and identified that *TGIF1* knockdown significantly suppressed trophoblast proliferation, invasion, migration, and the clonogenic ability (Fig. 7F–I, Supplementary Figs. 13F–J).

(4) Transcriptomic analysis: We performed an RNA-seq analysis on biological triplicates of control and shTGIF1 hTSCs, identifying the overall 6,486 differentially expressed genes (DEGs, $|\mathrm{Log_2Fold\ Change}| \geq 1$, $P_{\mathrm{adj.}} \leq 0.05$), with the majority being downregulated in the shTGIF1 group. GO and KEGG enrichment analyses indicated that these DEGs were involved in key biological processes and pathways such as cell proliferation, adhesion, and invasion (Fig. 8A, Supplementary Figs. 14A–B).

(5) Chromatin-binding analysis: We conducted a CUT&Tag assay for TGIF1 on biological duplicates of hTSCs, identifying over 3,000 high-confidence TGIF1-binding peaks that were mainly enriched in promoter and distal regulatory regions. The motif enrichment analysis revealed several known and novel transcription factor-binding motifs (Fig. 8B–D).

(6) Integration and target gene identification: By integrating the CUT&Tag and RNA-seq data, we identified 986 direct TGIF1 target genes. These included multiple key regulators implicated in trophoblast functions and pregnancy-related disorders such as preeclampsia and miscarriage, including *ADAM1*, *COL4A2*, *KISS1*, *SHH*, *WNT3A*, *RGL3*, *ZNF831*, and *TFDP1* (Fig. 8E–G, Supplementary Fig. 14E). Notably, many of these genes are well-established diagnostic markers for preeclampsia and pregnancy loss[13, 14, 15, 16, 17 17, 18].

The supplemental data offer robust mechanistic evidence that supports the regulatory role of *TGIF1* in trophoblast functions, thus making this study a mechanistically informed and functionally validated investigation into a key human-specific regulator in placental biology.

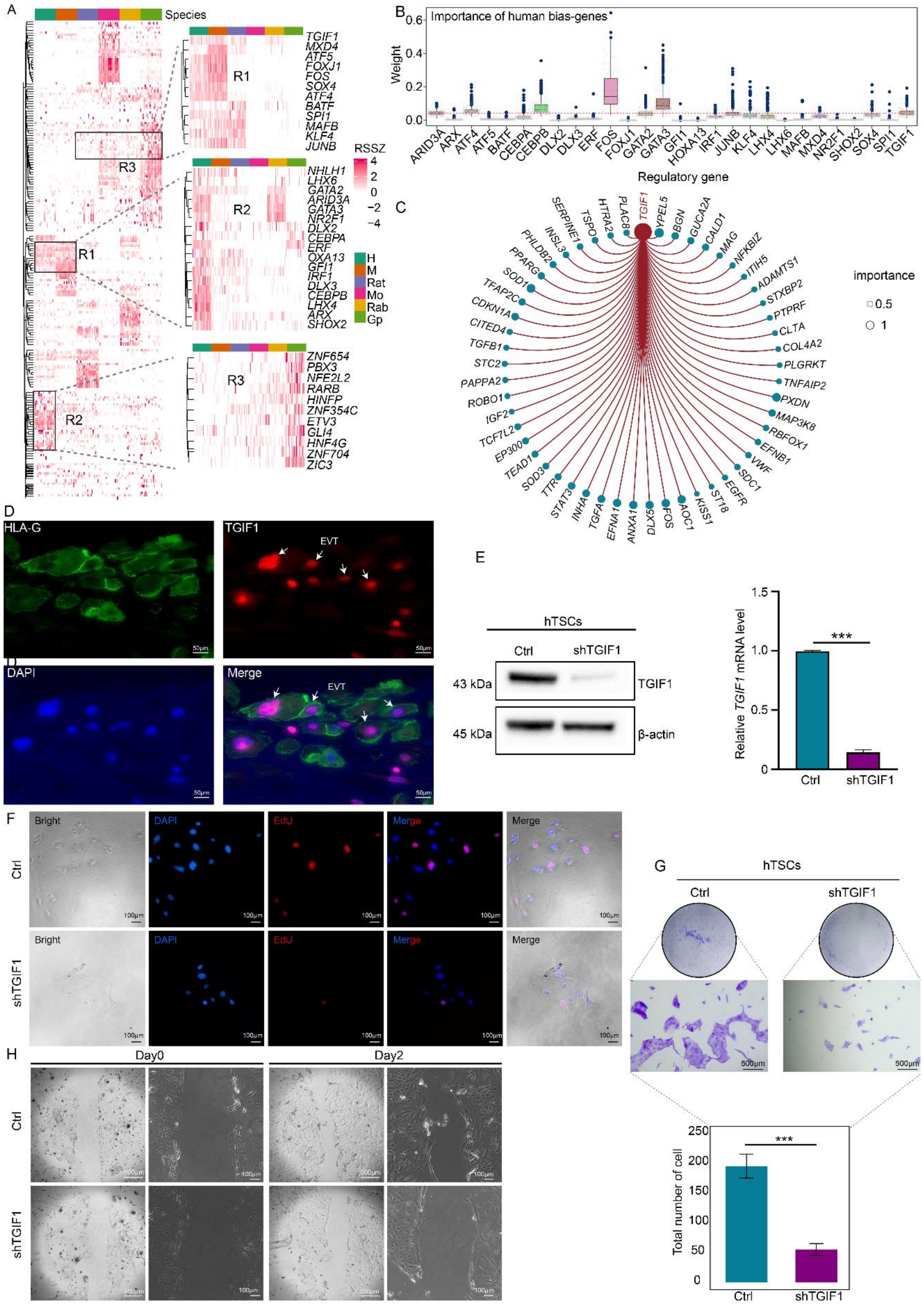

Fig.7: Key regulators of invasive trophoblasts and their potential roles in pregnancy disorders. (A) The heatmap displaying the activity of regulatory factors in invasive trophoblasts across six species. The regulatory

factors are categorized into different regions (R1, R2, and R3) based on conservation, with black boxes highlighting the clusters of highly specific regulatory factors in primates and rodents. (B) The correlation between human-biased genes (avg_log_2FC ≥ 1) and human-specific regulatory factors. (C) The regulatory network showing predicted *TGIF1* target genes in human EVTs. (D) The immunofluorescence analysis of human placental tissue (gestational week 40) showing TGIF1 staining (red) in the nuclei of HLA-G^+ EVTs (green). Nuclei were counterstained with DAPI (blue). Arrows indicate EVTs co-stained with TGIF1 and HLA-G. (E) TGIF1 knockdown in hTSCs confirmed by Western blotting (left) and qRT-PCR (right). β-actin was used as a loading control. (F) The EdU incorporation assay. Red, blue, and magenta indicate the EdU^+ cells, $DAPI^+$ nuclei, and the merge. (G) The transwell invasion assay showing the impaired invasive ability of TGIF1-deficient hTSCs. Representative images (top) and quantification of invaded cells (bottom) are shown. (H) The migration assay showing the reduced migratory capacity in shTGIF1 hTSCs on days 0 and 2 post-scratch. Scale bars are indicated. Data are presented as the mean ± SEM of biological triplicates (n = 3). ***: $P < 0.001$.

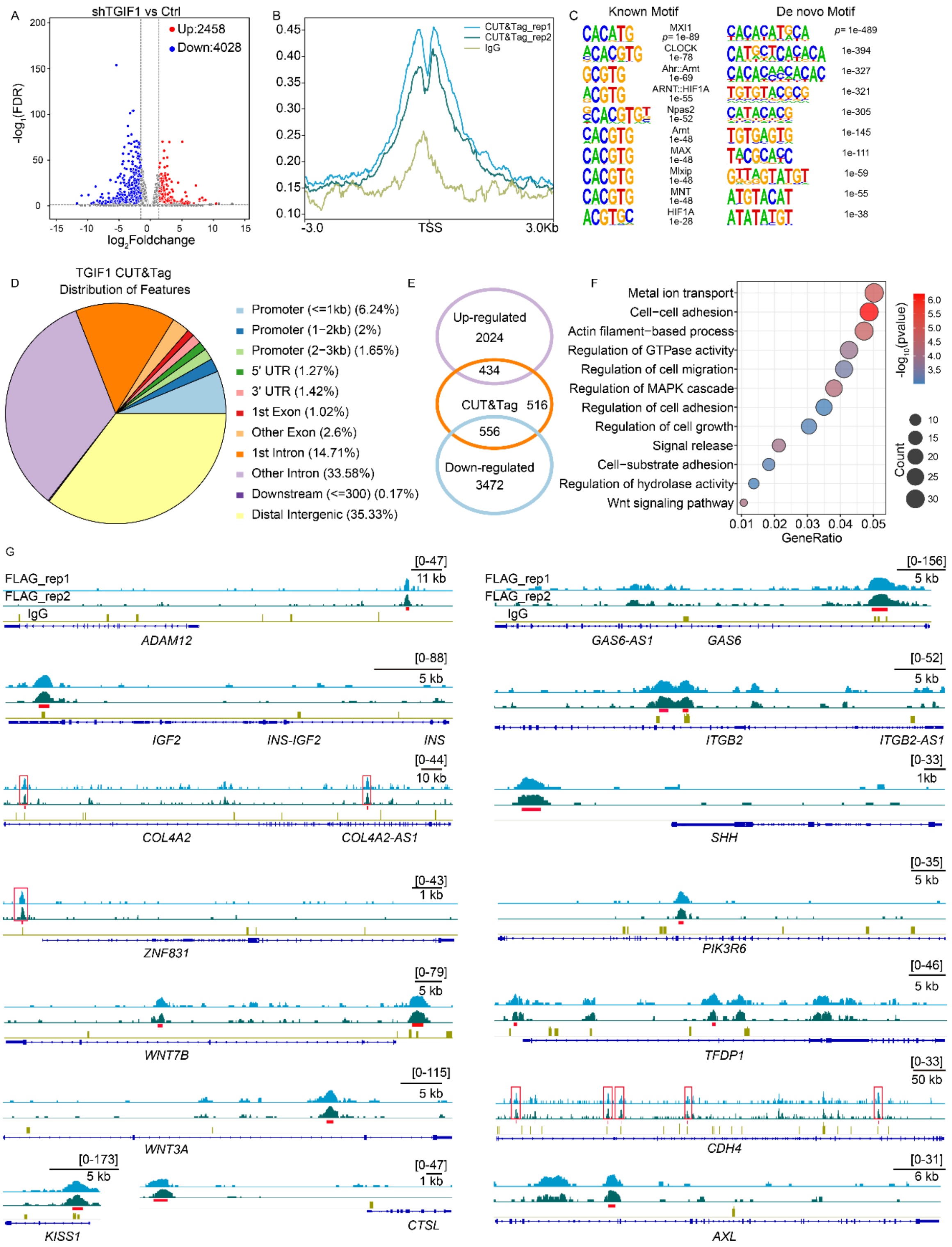

Fig.8: Genome-wide identification of the binding motifs of TGIF1. (A) The volcano plot of DEGs (shTGIF1 vs. control). (B) Distribution of TGIF1 CUT&Tag signals across transcription start sites (TSS) (±3 kb), showing promoter enrichment. (C) The DNA motif analysis of TGIF1 binding sites. Known motifs: previously reported motifs; *De novo* motifs: newly predicted motifs. (D) The genomic distribution profile of TGIF1 CUT&Tag peaks.

(E) The Venn diagram showing the overlap between genes associated with TGIF1-binding sites and the DEGs identified by RNA-seq. (F) GO terms for the genes associated with TGIF1-binding sites. (G) Chromatin binding peaks of TGIF1 at genomic regions associated with pregnancy disorder-related genes.

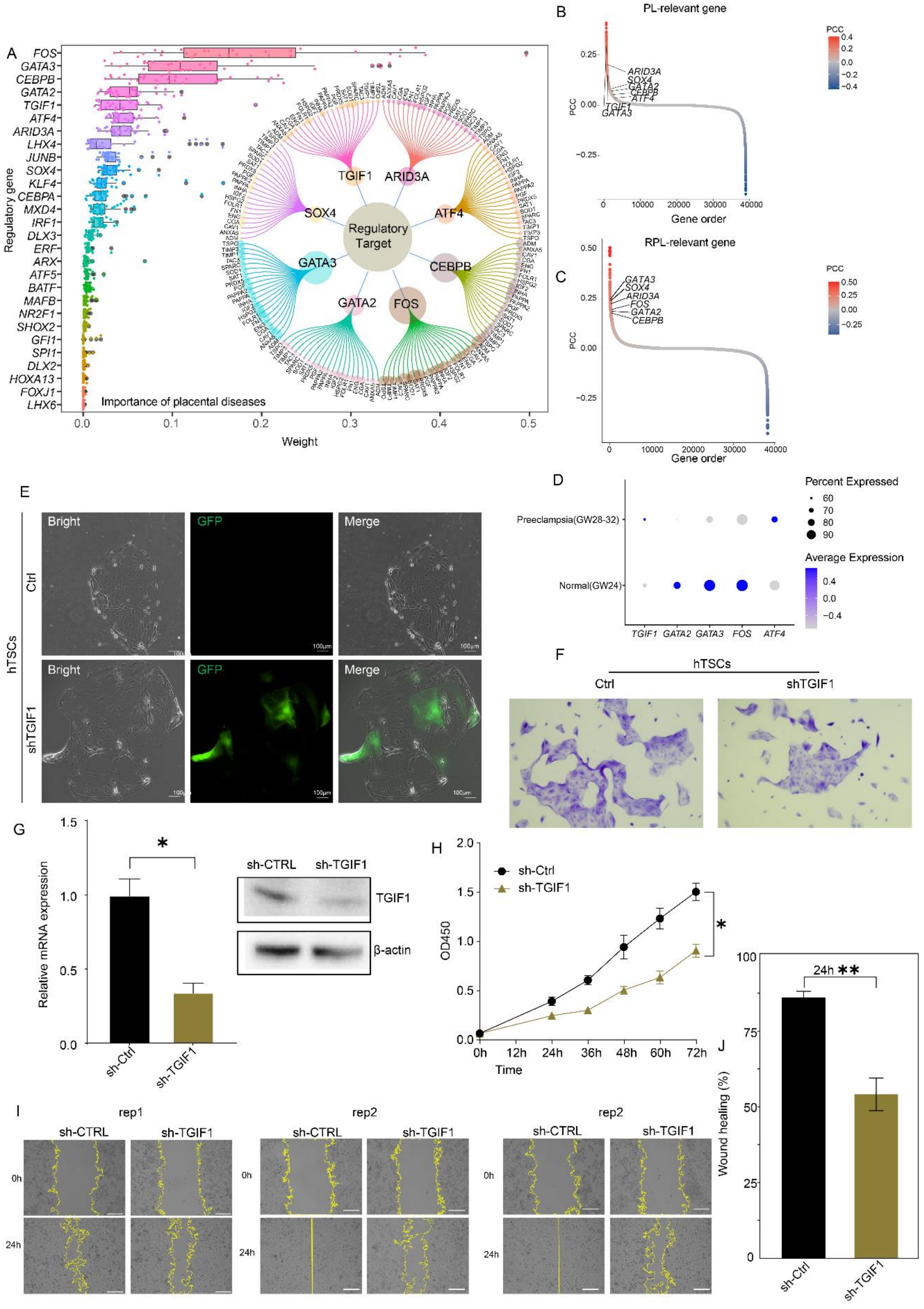

Supplementary Figs.13: The correlation between identified TFs and (recurrent) pregnancy loss.

(A) The analysis of different regulatory factors, revealing that the gene regulatory network demonstrates close

interactions between key TFs such as *TGIF1*, *FOS*, and *GATA3* and various high-risk genes associated with pregnancy-related diseases. (B and C) The ranking chart showing the correlation between several identified TFs and (recurrent) pregnancy loss in GWAS results. TFs of interest, such as *GATA3*, *SOX4*, *ARID3A*, *CEBPB*, and *FOS*, are annotated. (D) Gene expression in EVTs from early PE placentas and their normal counterparts. (E) Images of the transduced hTSCs. Bar = 100 μm. (F) The clonogenic assay for the control and shTGIF1 hTSCs. (G-I) Experiments were performed on HTR-8/SVneo cells to evaluate the effects of *TGIF1* knockdown. G, TGIF1 expression in the scramble control (sh-Ctrl) and *TGIF1*-knockdown (sh-*TGIF1*) groups was measured by qPCR and Western blot analyses. (H) A CCK-8 assay showing that knockdown of *TGIF1* in the HTR-8/SVneo cells significantly reduced cell proliferation after 72 h. (I) Representative images of the migration assay at 0 and 24 h after scratching the cell monolayer. The yellow lines indicate the boundary of the wound area. The left and right columns show cells transduced with the scramble control (sh-Ctrl) and *TGIF1*-target shRNA (sh-*TGIF1*), respectively. Bar = 100 μm. (J) The percentage of wound healing, revealing quantification of wound closure after 24 h. Data are presented as the mean ± SEM of biological triplicates (n = 3). *: $P < 0.05$; **: $P < 0.01$.

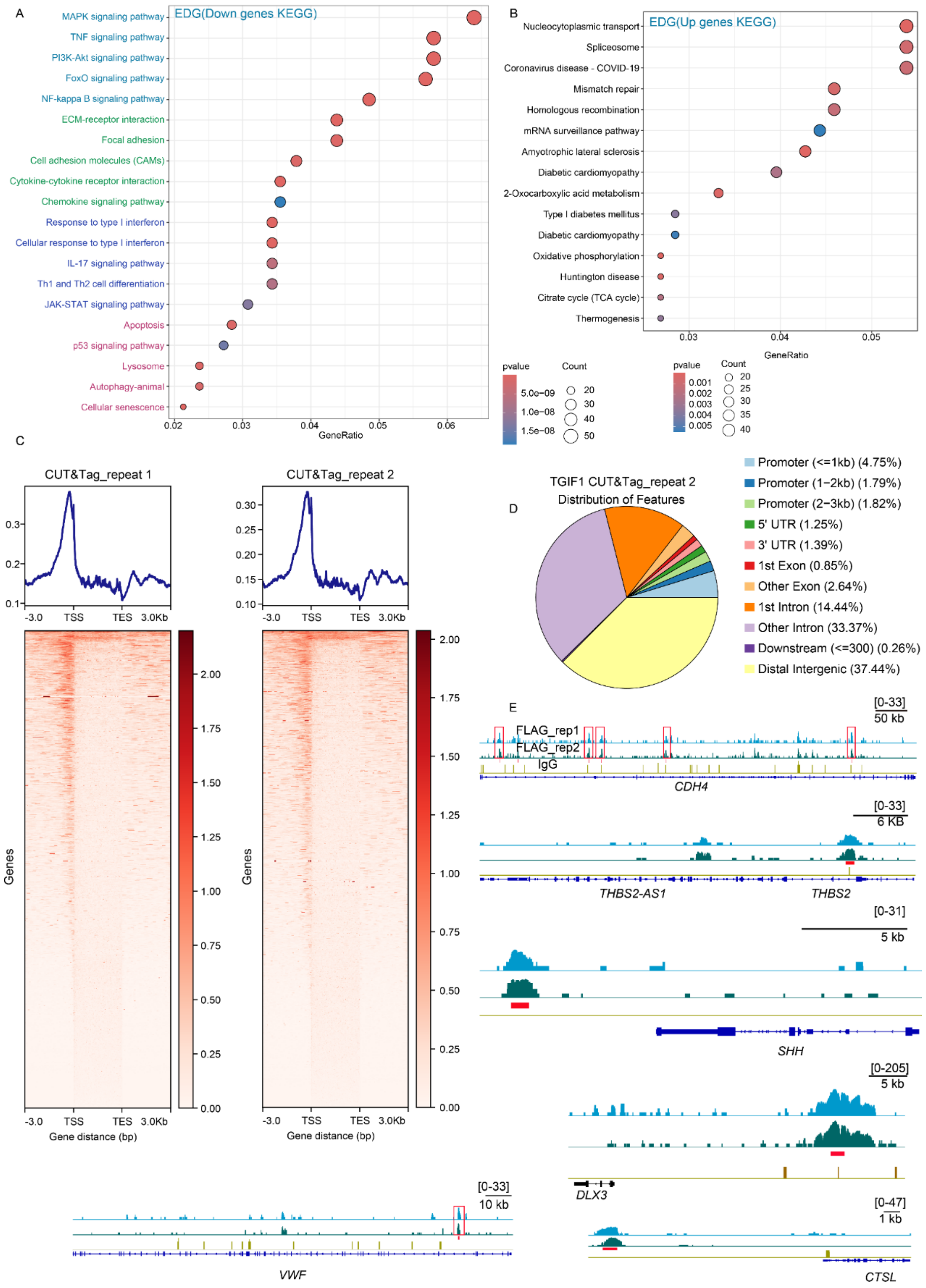
A
EDG(Down genes KEGG)
MAPK signaling pathway
TNF signaling pathway
PI3K-Akt signaling pathway
FoxO signaling pathway
NF-kappa B signaling pathway
ECM-receptor interaction
Focal adhesion
Cell adhesion molecules (CAMs)
Cytokine-cytokine receptor interaction
Chemokine signaling pathway
Response to type I interferon
Cellular response to type I interferon
IL-17 signaling pathway
Th1 and Th2 cell differentiation
JAK-STAT signaling pathway
Apoptosis
p53 signaling pathway
Lysosome
Autophagy-animal
Cellular senescence
0.02 0.03 0.04 0.05 0.06
GeneRatio
pvalue
5.0e−09
1.0e−08
1.5e−08
Count
20
30
40
50
B
EDG(Up genes KEGG)
Nucleocytoplasmic transport
Spliceosome
Coronavirus disease - COVID-19
Mismatch repair
Homologous recombination
mRNA surveillance pathway
Amyotrophic lateral sclerosis
Diabetic cardiomyopathy
2-Oxocarboxylic acid metabolism
Type I diabetes mellitus
Diabetic cardiomyopathy
Oxidative phosphorylation
Huntington disease
Citrate cycle (TCA cycle)
Thermogenesis
0.03 0.04 0.05
GeneRatio
pvalue
0.001
0.002
0.003
0.004
0.005
Count
20
25
30
35
40
C
CUT&Tag_repeat 1
CUT&Tag_repeat 2
-3.0 TSS TES 3.0Kb
Genes
Gene distance (bp)
D
TGIF1 CUT&Tag_repeat 2
Distribution of Features
Promoter (<=1kb) (4.75%)
Promoter (1−2kb) (1.79%)
Promoter (2−3kb) (1.82%)
5' UTR (1.25%)
3' UTR (1.39%)
1st Exon (0.85%)
Other Exon (2.64%)
1st Intron (14.44%)
Other Intron (33.37%)
Downstream (<=300) (0.26%)
Distal Intergenic (37.44%)
E
[0-33]
50 kb
FLAG_rep1
FLAG_rep2
IgG
CDH4
[0-33]
6 KB
THBS2-AS1
THBS2
[0-31]
5 kb
SHH
[0-205]
5 kb
DLX3
[0-33]
10 kb
VWF
[0-47]
1 kb
CTSL

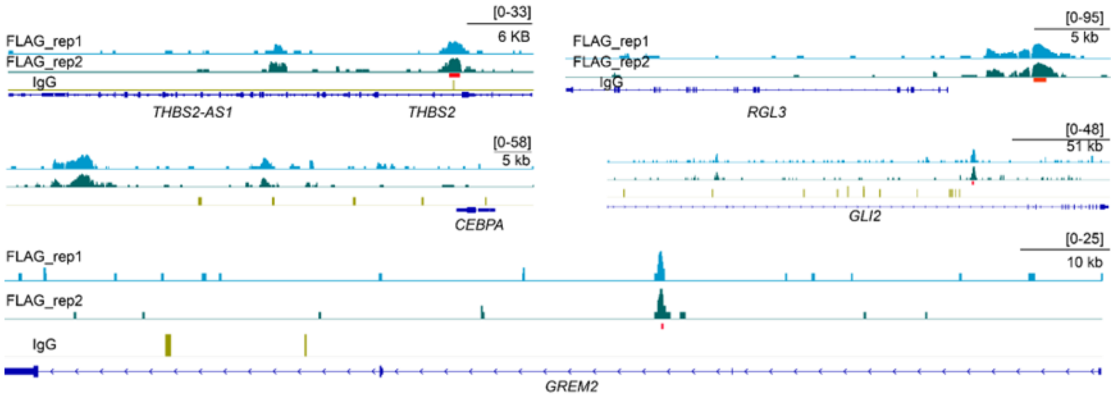

Supplementary Figs.14: Genome-wide identification of the binding motifs of TGIF1.

(A-B) GO and KEGG enrichment analyses of DEGs. (C) Heatmaps and average profiles of TGIF1 CUT&Tag signal intensity near transcription start sites (TSS) and transcription end sites (TES) in biological duplicates (repeat 1 and repeat 2). (D) Genomic distribution of TGIF1 CUT&Tag peaks based on feature annotations. (E) Genome browser tracks showing representative TGIF1 binding peaks.

2) The data is not accessible to review. The provided accession code PRJNA1177647 does not appear to link to any data on NCBI. This is not appropriate for a data analysis paper.

Response: We have kept PRJNA1177647 temporarily non-public to prevent premature release, but have provided a private reviewer-only link (https://dataview.ncbi.nlm.nih.gov/object/PRJNA1177647?reviewer=jsgtf0iutq4ecm439a81pgh6k8) granting full access to all raw data.

3) Why did the authors decide to include the datasets from Regan L. Scott et al. for rat placenta, given that Scott et al. removed placental tissue and associated decidua, performing scRNA-seq on uterine tissue at the site of trophoblast invasion? In addition, it is reported that the authors sequenced 4 samples for the timepoint E19.5, why the authors used only 3 samples? Which sample was excluded?

Response: We confirm that the dataset by Scott et al. originates from the uterine – placental interface, not the placenta proper. We included it specifically to capture invasive trophoblast populations (EVT/GC, etc.) for cross-species comparisons. In the manuscript, we consistently relabel it as "rat uterine – placental interface" and, after re-annotation, restrict analyses to invasive trophoblast subsets. In our evolutionary regression, the x-axis

represents human – other divergence time (MYA). Rat, mouse, guinea pig, and rabbit all fall at roughly the same anchor (~87 – 94 MYA) relative to human, so using rat does not change the x-axis placement or the overall trend and does not affect the conclusions. Moreover, interface samples better capture invasion-related adaptive features, which aligns with our aim to compare the cross-species adaptive evolution of invasive trophoblasts.

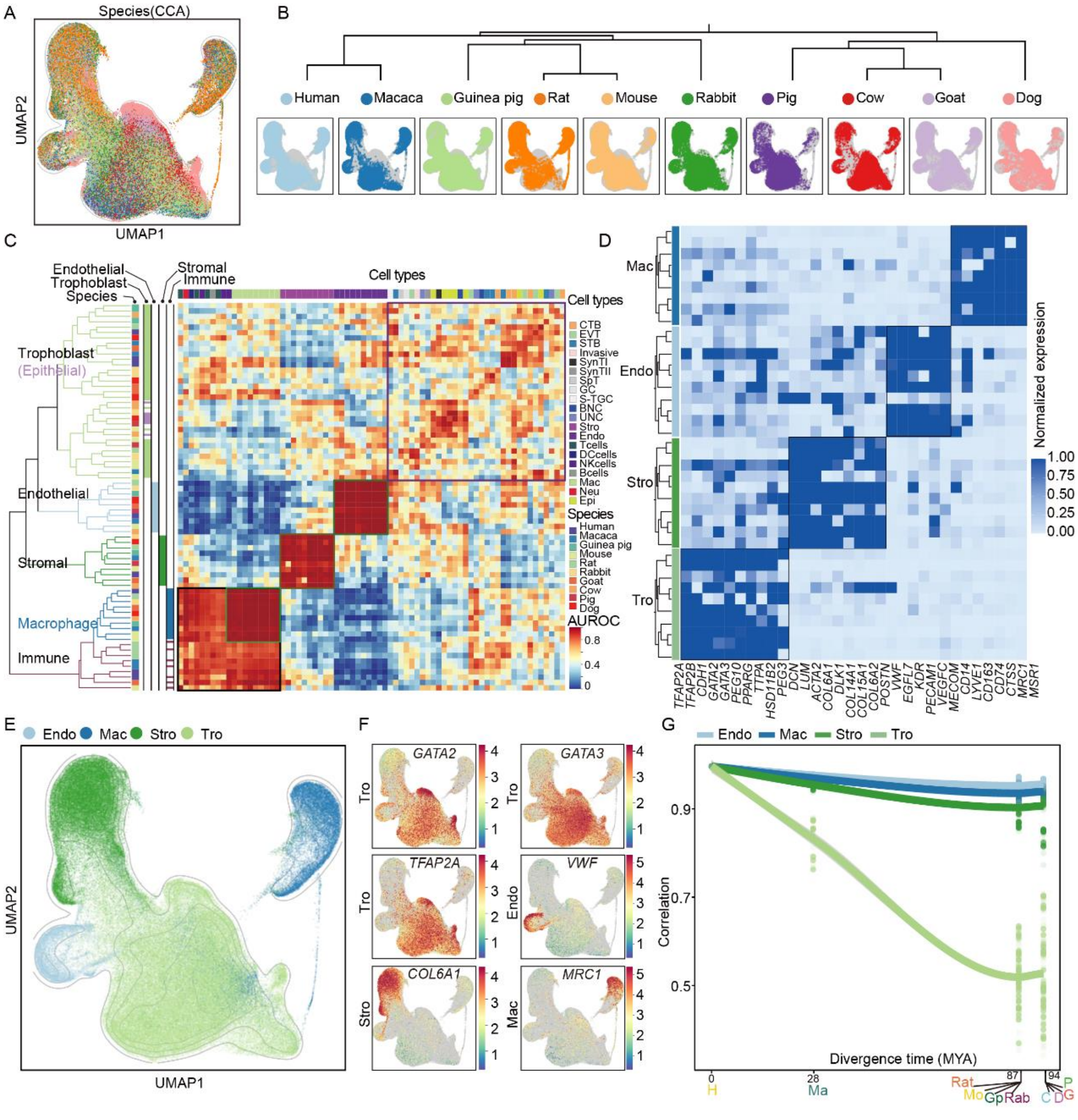

Fig.2: Conserved and divergent cell types during placental evolution

At the E19.5 time point, we are aware that the initial dataset comprised four biological replicates (GSM6241484_19_5_1-PD, GSM6241485_19_5_2-PD, GSM6241486_19_5_3-PD, and GSM8016898_19_5_7-PD). We excluded sample

GSM8016898_19_5_7-PD, from subsequent analyses due to reservations about its data representativeness and quality. In this particular sample, the percentage of cells with high mitochondrial content (>20%) was relatively low at 20.39%, and the total number of cells captured (7,808) was significantly fewer than in other samples (16,000 – 19,000), which could limit its ability to represent the full spectrum of transcriptional heterogeneity. Additionally, the median gene and UMI counts in this sample were marginally lower, indicating potential RNA degradation or reduced library complexity. Consequently, to ensure uniformity and reliability in cross-sample comparisons, we decided to exclude this sample from the final analysis. We apologize for the omission not being explicitly stated in the original manuscript and have clarified this in the revised version (lines 1142-1144, Supplementary Data 1).

Table S1 A summary of sequencing quality metrics for rat placental samples (E19.5)

sample	cell_number	avg_nCount_RNA	avg_nFeature_RNA	percent_low_quality(⩾20)	median_percent_mito
GSM6241484_19_5_1-PD	16481	7467.852	1806.166	29.82	12.589
GSM6241485_19_5_2-PD	19716	6576.741	1622.704	28.61	13.341
GSM6241486_19_5_3-PD	16618	7571.134	1755.217	31.8	13.354
GSM8016898_19_5_7-PD	7808	7314.513	1796.652	20.39	12.605

4) The authors combined new generated datasets and datasets already published. For the newly generated datasets, they used PISA for the reads alignment and gene counts. For the published datasets they reported:" We curated the published placental cell atlases from Homo sapiens, Cynomolgus macaque, Mus musculus, and Rattus norvegicus." What does this mean? Did the authors re-analyzed the datasets using PISA? If the authors did not reanalyzed the datasets, considering the differences between PISA and Cell Ranger (https://shiquan.github.io/PISA.html#difference-in-alignment-annotation-between-pisa-and-cellranger), could this generate differences between species?

Response: While we did not perform a systematic comparison between PISA and Cell Ranger, we thoroughly examined the official PISA user guide, which details the principal distinctions between the two pipelines, particularly regarding gene annotation and UMI processing. To guarantee the highest level of consistency and comparability across species, we reprocessed all publicly available datasets utilized in this study, encompassing those from humans, macaques, mice, and rats. This involved downloading or obtaining the original FASTQ files and aligning the reads using the PISA pipeline for both mapping and gene counting. We recognize the inadequate description of data processing in the original manuscript and have clarified the procedures applied to both newly generated and public datasets in the revised version (lines 1136-1142).

5) For quality control the authors removed cells with less than 20% of mitochondrial content using ATP6, ATP8, COX1, COX2, COX3, CYTB, ND1, ND2, ND3, ND4, ND4L, ND5, ND6 mitochondrial genes. Why did the authors not include the mitochondrial non-coding genes?

Response: We adhered to the quality control practices commonly employed for widely used single-cell transcriptomic analysis tools, such as Seurat and Scanpy, as well as those outlined in pertinent review articles[19, 20, 21, 22]. Throughout this study, we consistently utilized mitochondrial protein-coding genes (e.g., *ATP6*, *COX1*, *ND1*) to estimate mitochondrial content across all species. We excluded mitochondrial non-coding genes (such as tRNAs and rRNAs) from this calculation because these transcripts generally exhibit very low abundance in scRNA-seq data, and their annotations differ significantly across species. By not including them, we aimed to maintain consistency and comparability in mitochondrial quality control across all datasets included in the cross-species analysis.

Furthermore, when processing the downloaded public datasets[1, 3, 4], we specifically retrieved and analyzed the original count matrices to determine whether their criteria for mitochondrial gene filtering aligned with ours. Our analysis confirmed that mitochondrial protein-coding genes (e.g., *ATP6*, *COX1*, *ND1*) were also utilized to evaluate mitochondrial content during cell filtering in those studies.

6) Why the authors decided to integrate their datasets using CCA? Did they benchmark the other methods using the BENGAL pipeline (https://github.com/Papatheodorou-Group/BENGAL)?

Response: We did not utilize the BENGAL tool in this study. Instead, we employed the widely recognized scIB framework to benchmark single-cell transcriptomic data integration methods[23]. The scIB framework has been widely adopted by many researchers in the single-cell field[24, 25, 26, 27]. We systematically evaluated several commonly used integration strategies, such as CCA, scVI, Harmony, BBKNN, fastMNN, and Scanorama. The performance of each method was assessed across multiple biological conservation metrics (Isolated labels, Silhouette label, cLISI) and batch correction metrics (Silhouette batch, iLISI, Graph connectivity). These metrics were combined into an aggregate score to rank the overall performance. In our dataset, the benchmarking results indicated that CCA consistently performed well across key metrics and achieved the highest aggregate score (Table S2). Based on this, we selected CCA as the primary integration method in this study. We have detailed this benchmarking process in the revised manuscript and provided the corresponding evaluation results in the supplementary materials (lines 1199-1206, Supplementary Data 1).

Table S2. Benchmarking of data integration methods

**Metric type**	Bio conservation	Bio conservation	Bio conservation	Batch correction	Batch correction	Batch correction	Aggregate score	Aggregate score	Aggregate score
**Methods**	Isolated labels	Silhouette label	cLISI	Silhouette batch	iLISI	Graph connectivity	Bio conservation	Batch correction	Total
**Seurat(CCA)**	0.85596175	0.63799761	0.98296936	0.86496312	0.21452523	0.95571152	0.82564291	0.67839996	0.75202143
**scVI**	0.7125953	0.57097634	0.99922644	0.86767172	0.03630994	0.99266370	0.76093269	0.63221512	0.69657391
**Harmony**	0.52868706	0.52698570	0.99479969	0.89390973	0.13228754	0.87968623	0.68349082	0.63529450	0.65939266
**BBKNN**	0.5262205	0.47203607	0.99664647	0.88071238	0.03968789	0.91706394	0.66496768	0.61248807	0.63872788
**fastMNN**	0.427455	0.46928608	0.99719219	0.91211739	0.03383968	0.88502051	0.63131109	0.61032586	0.62081847
**Scanorama**	0.41916242	0.45544320	0.99890133	0.92471964	0.02812550	0.72206265	0.62450232	0.55830260	0.59140246

cLISI: cell Labeling In Silico Integration Score;

iLISI: integration Labeling In Silico Integration Score

7) In this atlas the authors should show the results after the integration of all the species to clearly demonstrate that all datasets are comparable. The authors only showed Figure 1C and 1D with all the species and cell types, but from this Figure it is not clear if the batch effect was corrected. In addition, it seems like some cell types were removed (ex. Absence of blood cell for rabbit).

Response: We have revised Fig. 1 as bellow; and the cross-species CCA integration is presented in Fig. 2A, demonstrating alignment and comparability across species.

Because blood cells are not structural components of the placenta, exhibit high cross-species variability, and introduce noise, we uniformly excluded them from all species prior to cross-species integration (see Methods; pre-filtering compositions in Fig. 1 and Supplementary Fig. 1).

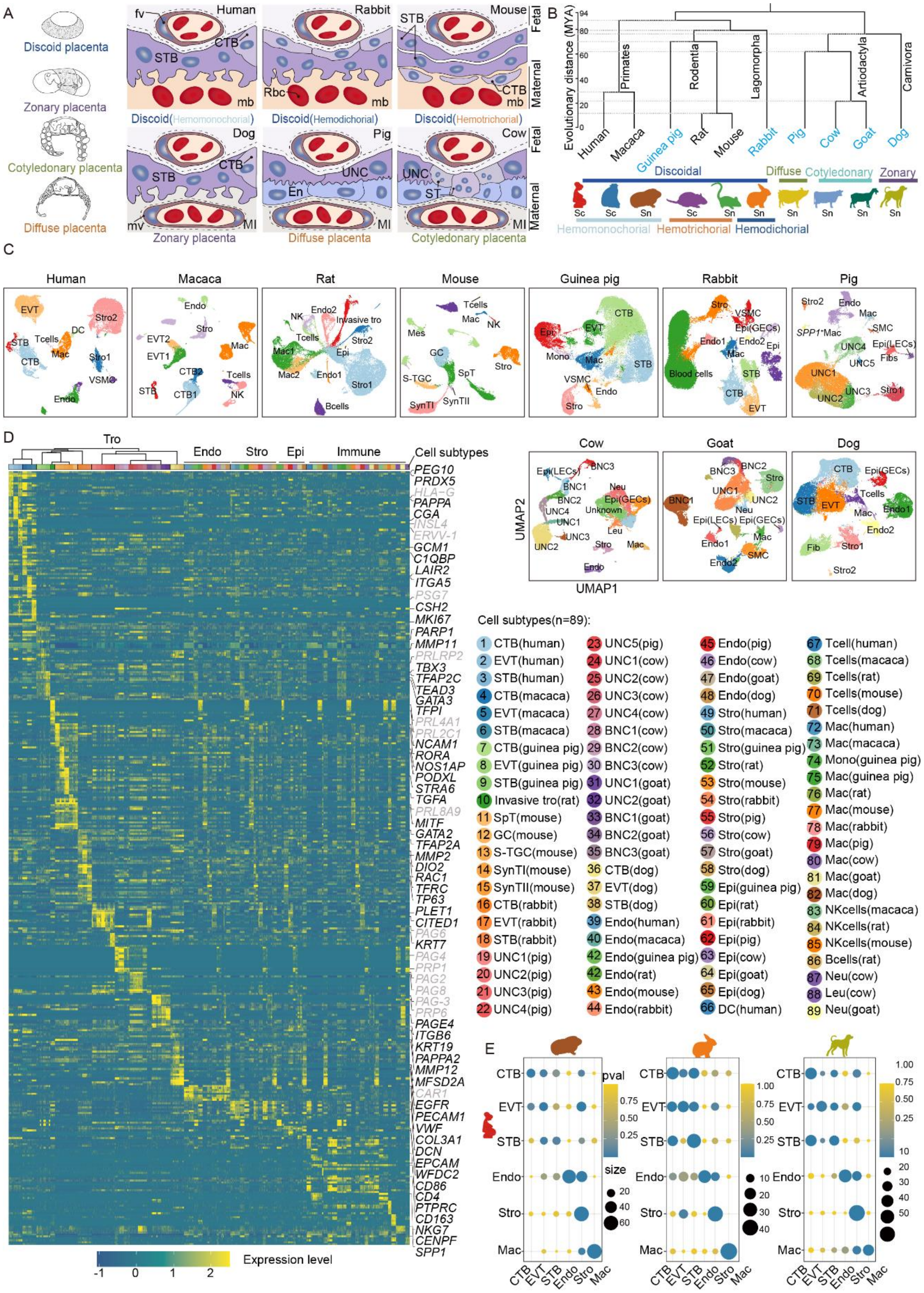

Fig.1: Single-cell / -nucleus transcriptomic mapping and cell type analysis of the mammalian placentas.

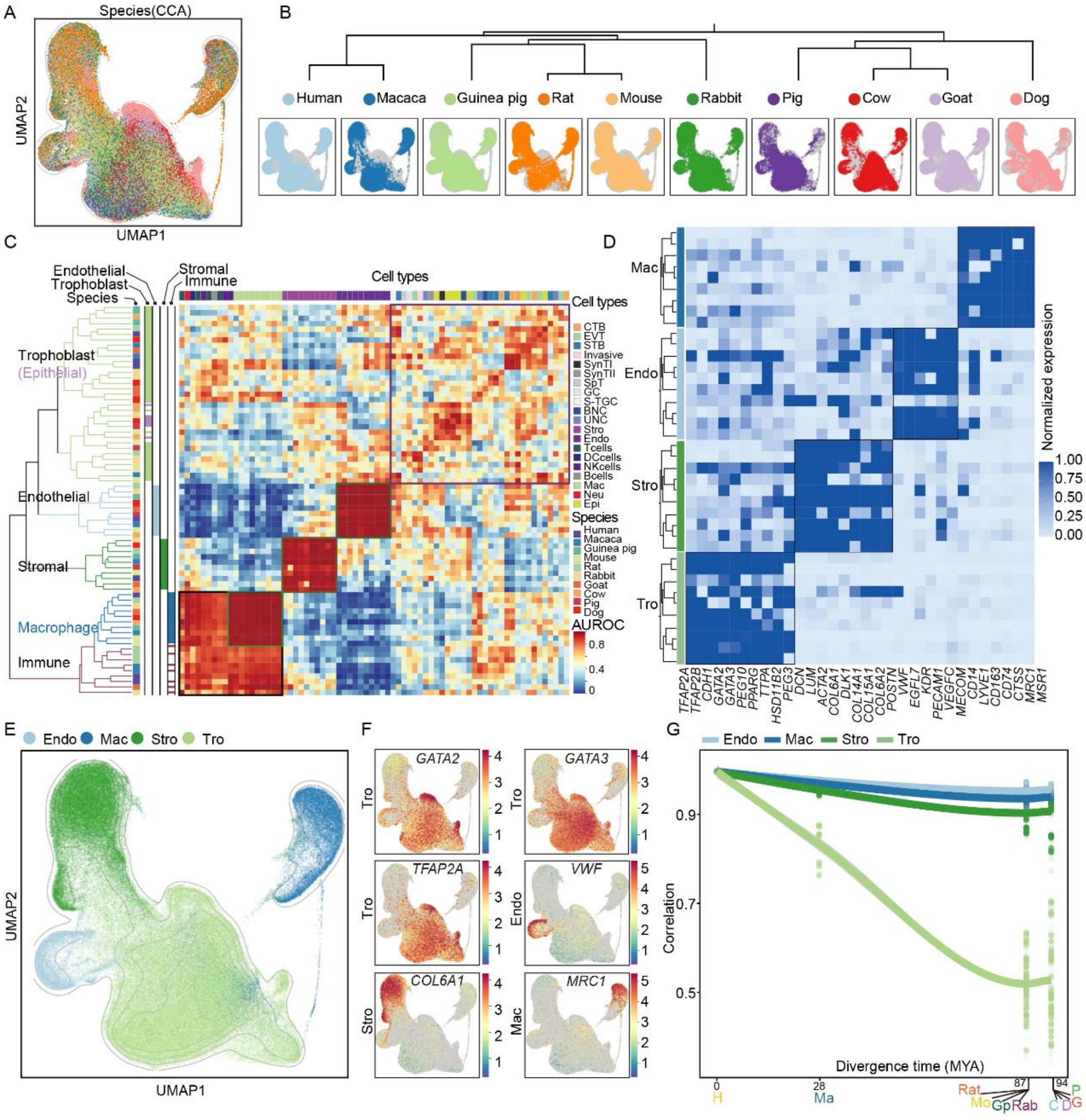

Fig.2: Conserved and divergent cell types during placental evolution

Minor Points:

1) The authors reported:" FindClusters functions were then employed to identify clusters based on principal components with a P value < 0.01". What does this mean?

Response: We drew on a phrase in a previous publication[19] that described clustering "based on principal components with a *P* value < 0.01". We acknowledge that this phrase might be misleading. In our analysis, we performed the JackStraw analysis to select principal components with *P* < 0.01, and then used these components as input for the

Seurat's FindClusters function. The clustering itself does not depend on P values, but instead uses graph-based clustering (FindNeighbors) built from the selected PCs. In the revised manuscript, we have clarified that $P < 0.01$ refers to the PC selection criterion, rather than a parameter of the clustering algorithm (lines 1156-1159).

2) The authors should include a Supplementary Table reporting the number of cells per datasets before and after QC.

Response: We fully agree with your suggestion and have included a Supplementary Table in the revised manuscript, which reports the number of cells per dataset before and after quality control (QC) (Supplementary Data 1).

References

1. Tsang JCH, *et al.* Integrative single-cell and cell-free plasma RNA transcriptomics elucidates placental cellular dynamics. *Proceedings of the National Academy of Sciences* **114**, E7786-E7795 (2017).
2. Jiang X, *et al.* Identifying a dynamic transcriptomic landscape of the cynomolgus macaque placenta during pregnancy at single-cell resolution. *Developmental Cell* **58**, 806-821.e807 (2023).
3. Scott RL, Vu HTH, Jain A, Iqbal K, Tuteja G, Soares MJ. Conservation at the uterine–placental interface. *Proceedings of the National Academy of Sciences* **119**, e2210633119 (2022).
4. Marsh B, Blelloch R. Single nuclei RNA-seq of mouse placental labyrinth development. *eLife* **9**, e60266 (2020).
5. El-Jaick KB, *et al.* Functional analysis of mutations in TGIF associated with holoprosencephaly. *Mol Genet Metab* **90**, 97-111 (2007).
6. Bertolino E, Reimund B, Wildt-Perinic D, Clerc RG. A novel homeobox protein which recognizes a TGT core and functionally interferes with a retinoid-responsive motif. *J Biol Chem* **270**, 31178-31188 (1995).
7. Hamid R, Patterson J, Brandt SJ. Genomic structure, alternative splicing and expression of TG-interacting factor, in human myeloid leukemia blasts and cell lines. *Biochim Biophys Acta* **1779**, 347-355 (2008).
8. Bartholin L, *et al.* Maternal Tgif is required for vascularization of the embryonic placenta. *Dev Biol* **319**, 285-297 (2008).
9. Pathirage NA, *et al.* Homeobox gene transforming growth factor β-induced factor-1 (TGIF-1) is a regulator of villous trophoblast differentiation and its expression is increased in human idiopathic fetal growth restriction. *Molecular Human Reproduction* **19**, 665-675 (2013).
10. Yu X, *et al.* Acetyl-CoA metabolism maintains histone acetylation for syncytialization of human placental trophoblast stem cells. *Cell Stem Cell* **31**, 1280-1297.e1287 (2024).
11. Chen Y, *et al.* SP6 controls human cytotrophoblast fate decisions and trophoblast stem cell establishment by targeting MSX2 regulatory elements. *Developmental Cell*

59, 1506-1522.e1511 (2024).

12. Wu H, Wang Y, Wang H. Generation of Human Trophoblast Stem Cell-Dependent Placental In Vitro Models. *Methods in molecular biology (Clifton, NJ)* **2767**, 43-52 (2024).
13. Chen CP. Placental abnormalities and preeclampsia in trisomy 13 pregnancies. *Taiwan J Obstet Gynecol* **48**, 3-8 (2009).
14. Hiden U, Bilban M, Knöfler M, Desoye G. Kisspeptins and the placenta: regulation of trophoblast invasion. *Rev Endocr Metab Disord* **8**, 31-39 (2007).
15. Pan Y*, et al.* Dysfunction of Shh signaling activates autophagy to inhibit trophoblast motility in recurrent miscarriage. *Exp Mol Med* **53**, 52-66 (2021).
16. Steinthorsdottir V*, et al.* Genetic predisposition to hypertension is associated with preeclampsia in European and Central Asian women. *Nat Commun* **11**, 5976 (2020).
17. Honigberg MC*, et al.* Polygenic prediction of preeclampsia and gestational hypertension. *Nat Med* **29**, 1540-1549 (2023).
18. Christians JK, Beristain AG. ADAM12 and PAPP-A: Candidate regulators of trophoblast invasion and first trimester markers of healthy trophoblasts. *Cell Adh Migr* **10**, 147-153 (2016).
19. Wang F*, et al.* Endothelial cell heterogeneity and microglia regulons revealed by a pig cell landscape at single-cell level. *Nat Commun* **13**, 3620 (2022).
20. Waickman AT*, et al.* mRNA-1273 vaccination protects against SARS-CoV-2-elicited lung inflammation in nonhuman primates. *JCI Insight* **7**, (2022).
21. Hochane M*, et al.* Single-cell transcriptomics reveals gene expression dynamics of human fetal kidney development. *PLoS Biol* **17**, e3000152 (2019).
22. Ilicic T*, et al.* Classification of low quality cells from single-cell RNA-seq data. *Genome Biology* **17**, 29 (2016).
23. Luecken MD*, et al.* Benchmarking atlas-level data integration in single-cell genomics. *Nature Methods* **19**, 41-50 (2022).
24. Zhang S*, et al.* Cross-species single-cell analysis reveals divergence and conservation of peripheral blood mononuclear cells. *BMC genomics* **25**, 1169 (2024).
25. Korsunsky I*, et al.* Cross-tissue, single-cell stromal atlas identifies shared pathological fibroblast phenotypes in four chronic inflammatory diseases. *Med* **3**, 481-518.e414 (2022).
26. Qin Y*, et al.* 48-Hour and 24-Hour Time-lapse Single-nucleus Transcriptomics Reveal Cell-type specific Circadian Rhythms in Arabidopsis. *Nat Commun* **16**, 4171 (2025).
27. He Z*, et al.* An integrated transcriptomic cell atlas of human neural organoids. *Nature* **635**, 690-698 (2024).

Response to the reviewers' comments

Below we provide the point-by-point response.

***Note*: Changes in the revised manuscript are highlighted in red.**

REVIEWERS' COMMENTS

Reviewer #1 (Remarks to the Author):

The revised manuscript by Tan et al is much improved and they have added a significant amount of work addressing TGIF1 function.

We thank the reviewer for the encouraging remarks and acknowledging the improvements made to the manuscript. We are pleased that the additional work addressing TGIF1 function has strengthened the study. We appreciate the reviewer's time and thoughtful evaluation throughout the review process.

The authors have addressed my first four minor points regarding presentation of the bioinformatic data.

While the expanded analysis of TGIF1 function is impressive, there are still a few mostly relatively minor concerns with the data.

Line 839 – should this refer to supplemental Fig 13E or G?

Response: The citation in line 839 was indeed incorrect. After reordering the panels of Supplementary Fig. 13, the relevant result now corresponds to Supplementary Figs. 13E and 13F, and the text has been corrected accordingly. The entire manuscript has also been checked, and no other similar issues were found.

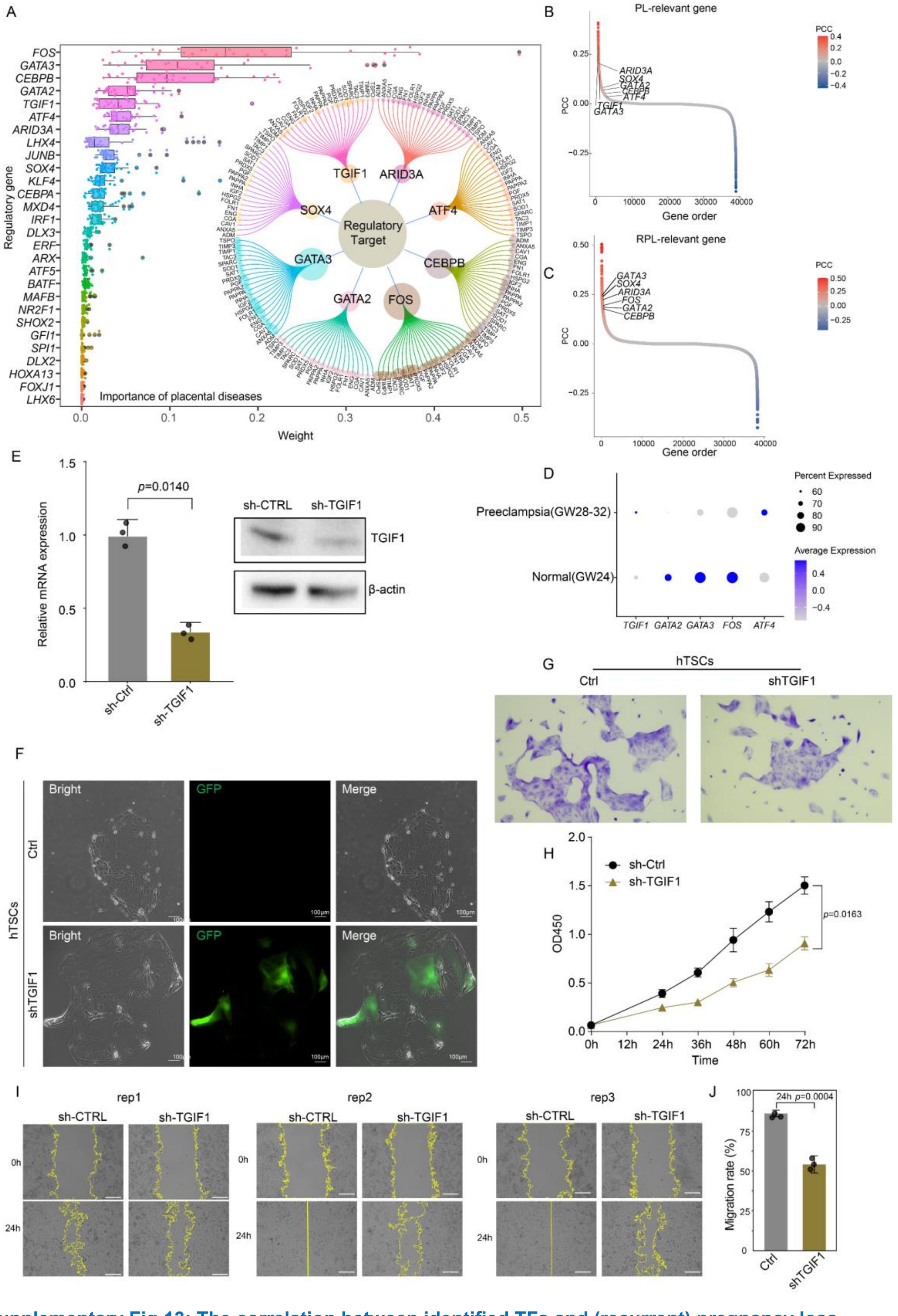

Supplementary Fig.13: The correlation between identified TFs and (recurrent) pregnancy loss.
(A) Distribution of inferred regulatory weights between 28 human-specific TFs and 404 human-biased genes in EVTs. Each dot represents the inferred regulatory weight of one TF-gene pair, and box plots summarize the

distribution of weights across all human-biased genes for each TF. Center line, median; box limits, 25th and 75th percentiles; whiskers, 1.5 × IQR; points, outliers. Regulatory weights were inferred using 3,811 EVT cells derived from six independent human placental samples. (B and C) The ranking chart showing the correlation between several identified TFs and (recurrent) pregnancy loss in GWAS results. TFs of interest, such as *GATA3*, *SOX4*, *ARID3A*, *CEBPB*, and *FOS*, are annotated. (D) Gene expression in EVTs from early PE placentas and their normal counterparts. (E) TGIF1 expression in scramble control (sh-Ctrl) and *TGIF1*-knockdown (sh-*TGIF1*) HTR-8/SVneo cells was measured by qPCR and western blot analysis. (F) Images of the transduced hTSCs. Bar = 100 μm. Dots indicate biologically independent replicates. Statistical significance was assessed using a two-sided Welch's t-test. (G) The clonogenic assay for the control and sh-*TGIF1* hTSCs. (H) The CCK-8 assay showing reduced proliferation of *TGIF1*-knockdown HTR-8/SVneo cells. Data are presented as the mean ± SEM of biological triplicates (n = 3). Statistical significance was assessed using a two-sided Welch's t-test at 72 h. (I) Representative images of the migration assay in HTR-8/SVneo cells at 0 and 24 h after scratching the cell monolayer. The yellow lines indicate the boundary of the wound area. The left and right columns show cells transduced with the scramble control (sh-Ctrl) and *TGIF1*-targeting shRNA (sh-*TGIF1*), respectively. Scale bar, 100 μm. (J) Quantification of wound closure in HTR-8/SVneo cells after 24 h, shown as the percentage of wound healing. Data are presented as the mean ± SEM of biological triplicates (n = 3). Dots indicate biologically independent replicates. Statistical significance was assessed using a two-sided Welch's t-test.

Line 840 – figure 7F-I. This figure does not have panel I.

Response: We thank the reviewer for pointing out this discrepancy. In the previous version, 'Panel I' was mentioned in the text but absent in the figure layout. Following the reviewer's suggestion, we have incorporated the statistical quantification for the transwell migration assay into panel G (so there is no panel I in the updated version).

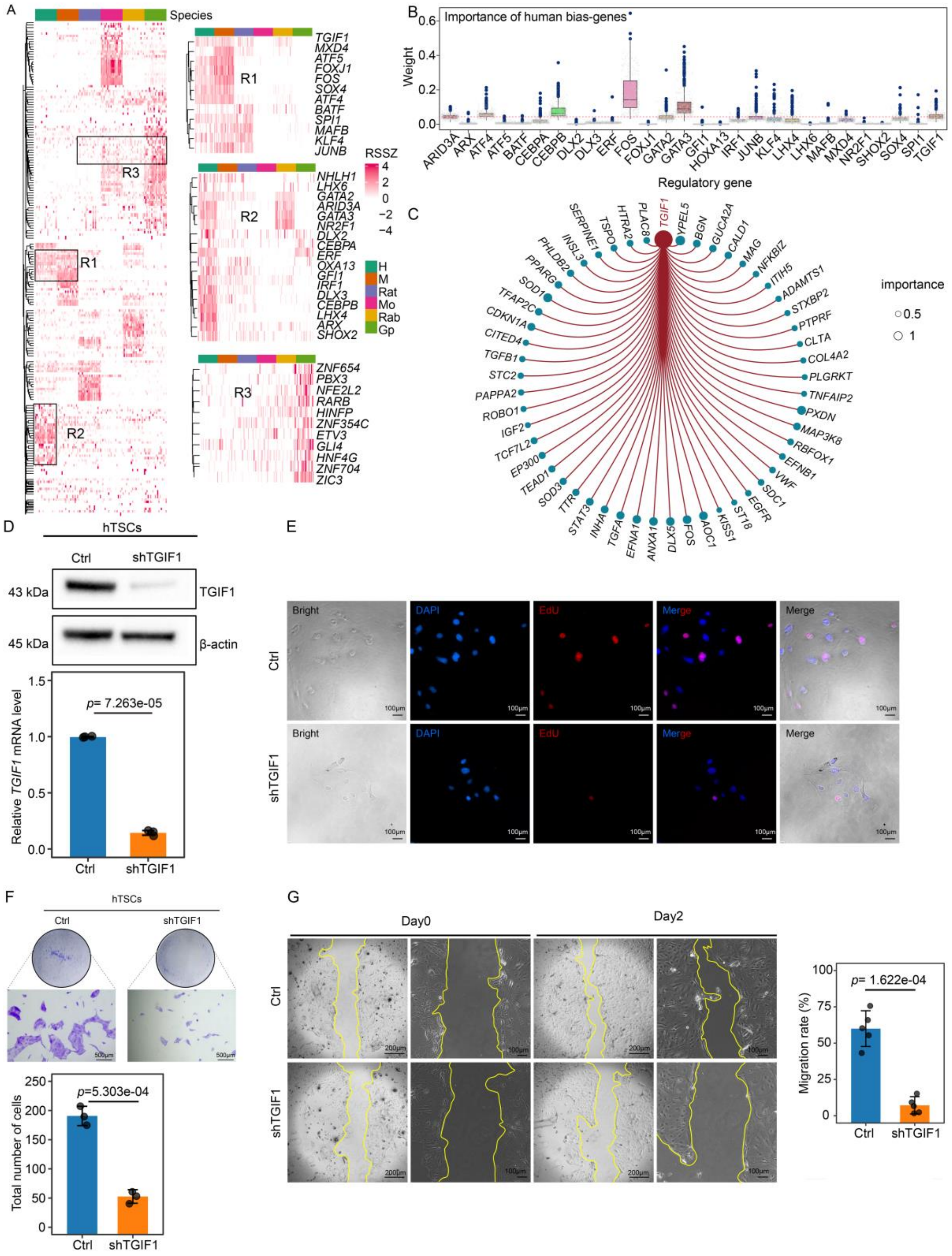

Fig.7: Key regulators of invasive trophoblasts and their potential roles in pregnancy disorders. (A) The heatmap displaying the activity of regulatory factors in invasive trophoblasts across six species. The regulatory factors are categorized into different regions (R1, R2, and R3) based on conservation, with black boxes highlighting the clusters of highly specific regulatory factors in primates and rodents. (B) Distribution of inferred

regulatory weights between 28 human-specific TFs and 404 human-biased genes (avg_log2FC ⩾ 1) in EVTs. Each dot represents the inferred regulatory weight of one TF-gene pair, and box plots summarize the distribution of weights across all human-biased genes for each TF. Center line, median; box limits, 25th and 75th percentiles; whiskers, 1.5 × IQR; points, outliers. Regulatory weights were inferred using 3,811 EVT cells derived from six independent human placental samples. (C) The regulatory network showing predicted TGIF1 target genes in human EVTs. (D) TGIF1 knockdown in hTSCs confirmed by Western blotting (top) and qRT-PCR (bottom). β-actin was used as a loading control. Data are presented as the mean ± SEM of biological triplicates (n = 3). Dots indicate biologically independent replicates. Statistical significance was assessed using a two-sided Welch's t-test. (E) The EdU incorporation assay. Red, blue, and magenta indicate the EdU^+ cells, $DAPI^+$ nuclei, and the merge. Representative images from two biologically independent experiments with similar results are shown. (F) The transwell migration assay showing the impaired migratory ability of *TGIF1*-knockdown hTSCs. Representative images (top) and quantification of migrated cells (bottom) are shown. Data are presented as the mean ± SEM of biological triplicates (n = 3). Dots indicate biologically independent replicates. Statistical significance was assessed using a two-sided Welch's t-test. (G) The migration assay showing the reduced migratory capacity in sh*TGIF1* hTSCs on days 0 and 2 post-scratch. Scale bars are indicated. Representative images are shown on the left, and quantification is shown on the right. Scale bars are indicated. Data are presented as the mean ± SEM of five biological replicates (n = 5). Dots indicate biologically independent replicates. Statistical significance was assessed using a two-sided Welch's t-test.

I think a few extra lines of description of what is being done in panels 7F-H is needed in the main text. (also for supp 13F-J). Otherwise it is hard to judge how the conclusions are being drawn.

Response: Thank you very much for this helpful suggestion. In response, we have revised the main text to provide a more detailed explanation of the experiments shown in Fig. 7E-G and Supplementary Fig. 13F-J, and have explicitly clarified how the conclusions were drawn (Lines 700-715).

The revised sentences are as follows:

"Based on these findings, we further explored the functional magnitude of *TGIF1* by performing knockdown experiments in both human trophoblast stem cells (hTSCs) and the HTR-8/SVneo cell line (a human chorionic trophoblast cell line) by introducing a *TGIF1*-shRNA expression vector into the cells via lentiviral transduction (Fig.7D, Supplementary Fig.13E and 13F). We observed that *TGIF1* knockdown significantly reduced the proportion of EdU^+ cells (Fig. 7E), indicating impaired cell proliferation. Consistently, the clonogenic and CCK-8 cell viability

assays demonstrated that *TGIF1* downregulation markedly compromised the clonogenic ability and the overall cell viability, respectively (Supplementary Fig. 13G, H). To assess the impact of *TGIF1* downregulation on cell motility, we performed Transwell migration (without Matrigel coating) and wound healing assays (Fig. 7F-G; Supplementary Fig. 13I, J). In hTSCs, *TGIF1* knockdown significantly decreased the number of migrating cells (Fig. 7F). Similarly, the wound healing assay revealed a markedly reduced migration rate in *TGIF1*-knockdown cells (Fig.7G; Supplementary Fig. 13I, J). Notably, *TGIF1* knockdown also impaired cell adhesion, as evidenced by partial cell detachment and increased cell death during the wound healing process (Fig.7G)."

Figure 7H needs quantification. Additionally these images are not very clear. Supplemental 13I looks much better.

Response: In the revised version, we have replaced the original images with high-resolution micrographs consistent with the visualization style in Supplemental Figure 13I. We have also readjusted the layout to ensure that cellular details and the wound gap are clearly discernible. Furthermore, we have incorporated the statistical quantification for the transwell migration assay into panel G. The analysis demonstrated a significant difference in migration capacity between two groups (*P* < 0.05). The corresponding figure legends (Lines 1571-1575) and the main text (Lines 710-715) have been updated to reflect these changes.

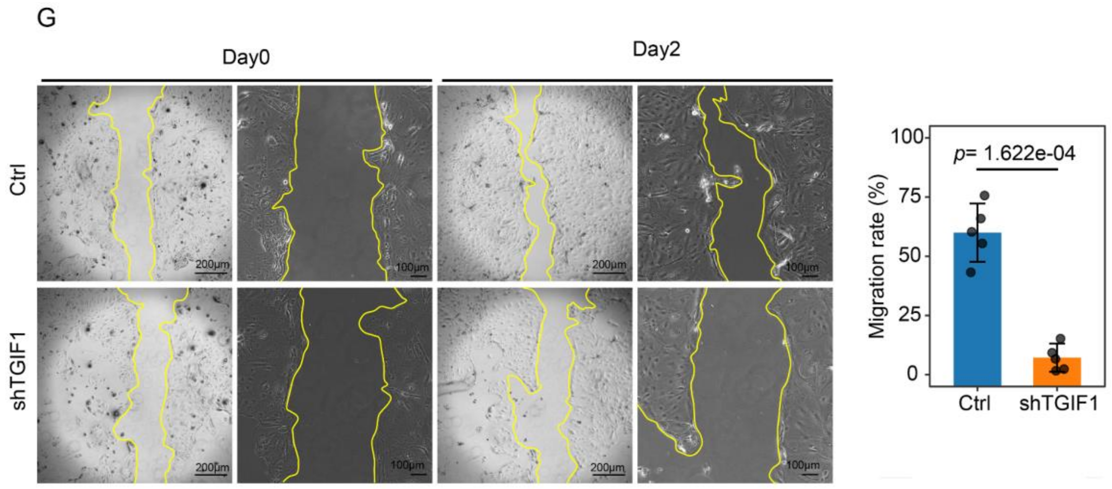

Figure 7G The migration assay showing the reduced migratory capacity in sh*TGIF1* hTSCs on days 0 and 2

post-scratch. Scale bars are indicated. Representative images are shown on the left, and quantification is shown on the right. Scale bars are indicated. Data are presented as the mean ± SEM of five biological replicates (n = 5). Dots indicate biologically independent replicates. Statistical significance was assessed using a two-sided Welch's t-test.

Line 881 – describes broad TGIF1 signal in the gene body, referring to Fig 8B. However, Fig 8B (and supplemental 14C) shows a peak around the TSS. More careful description of this panel and the data in Fig 8D is needed.

Response: We sincerely thank the reviewer for this critical observation. We agree that the term 'broad spectrum within the gene body' was inaccurate, as Figure 8B clearly demonstrates the sharp and characteristic enrichment of TGIF1 binding specifically at the transcription start site (TSS). In the revised manuscript, we have corrected this description to accurately reflect the TSS-proximal enrichment pattern. We have also reverified and updated the genomic distribution percentages to ensure full consistency with Figure 8D. These revisions ensure a more precise interpretation of our CUT&Tag data (Lines 740-748).

Line 884 refers to supplemental 14C for motifs, but that is not what it shows.

Response: We sincerely apologize for this reference error. The reviewer is correct that Supplementary Fig. 14C shows the CUT&Tag signal intensity heatmaps rather than the motif analysis results. In the revised manuscript, we have corrected the reference to Supplementary Fig. 14E that contains the motif enrichment analysis result. We have also carefully checked the relevant figure references in the revised manuscript to ensure consistency.

The motif analysis (Fig 8C) shows very few motifs – for example, among the known motifs three are exactly the same CACGTG motif, and almost all the rest are a version of this. Since the least significant of these is 1e-28, there are likely a lot of other significantly enriched motifs. It might be better to show a full list in the supplement, and version in Fig 8 that includes the best example of each class but goes further down the list. One question that comes up is whether somewhere in the list they find a TGIF1 (or MEIS TALE HD) binding motif (CTGTCA)?

Response: We thank the reviewer for this helpful suggestion. We agree that the original Fig. 8C overrepresents closely related CACGTG/E-box-like motifs because multiple top-ranked HOMER hits corresponded to highly similar motif classes. We have therefore revised Fig. 8C to display representative known and *de novo* motifs from distinct motif families/classes, rather than repeatedly showing closely related motifs. In addition, the full ranked lists of significantly enriched known and *de novo* motifs have been provided in Supplementary Figure 14D. We also specifically examined the full HOMER output for TGIF1/MEIS/TALE homeodomain-related motifs, including CTGTCA-like sequences, but did not detect significant enrichment of these motifs in this dataset.

For the analysis of the overlap between TGIF1 peaks and DEGs (Fig 8E,F), it might make more sense to look at gene set enrichment for TGIF1 bound and up-regulated vs TGIF1 bound and down-regulated. Given the known function of TGIF1 as a repressor, the bound and up-regulated (in the knock-down) might include direct targets.

Response: We sincerely thank the reviewer for this insightful and constructive suggestion. We fully agree that TGIF1 predominantly functions as a transcriptional repressor, and thus the genes that were both bound by TGIF1 and upregulated upon its knockdown represent the most likely direct targets. During the revision, we have performed separate GO enrichment analyses for TGIF1-bound/up-regulated and TGIF1-bound/down-regulated gene sets. As expected, the bound/up-regulated targets were significantly enriched with biological processes such as small GTPase-mediated signal transduction, cytoskeleton organization, cell growth, and cell cycle regulation. These pathways are highly consistent with the defective migration and proliferation phenotypes observed in our *TGIF1*-knockdown trophoblast cells. Furthermore, we have provided genome browser tracks (IGV) to visualize representative TGIF1-binding peaks at the promoter regions of key target genes (e.g., *WNT3A*, *ZNF831*, *ITGB2*, *CTSL*, *EGFR*, and *DLX3*). These new analyses and visualization have been integrated into the revised Figure 8E, Supplementary Figure 14E-F, and the corresponding Results

section (Lines 750-772).

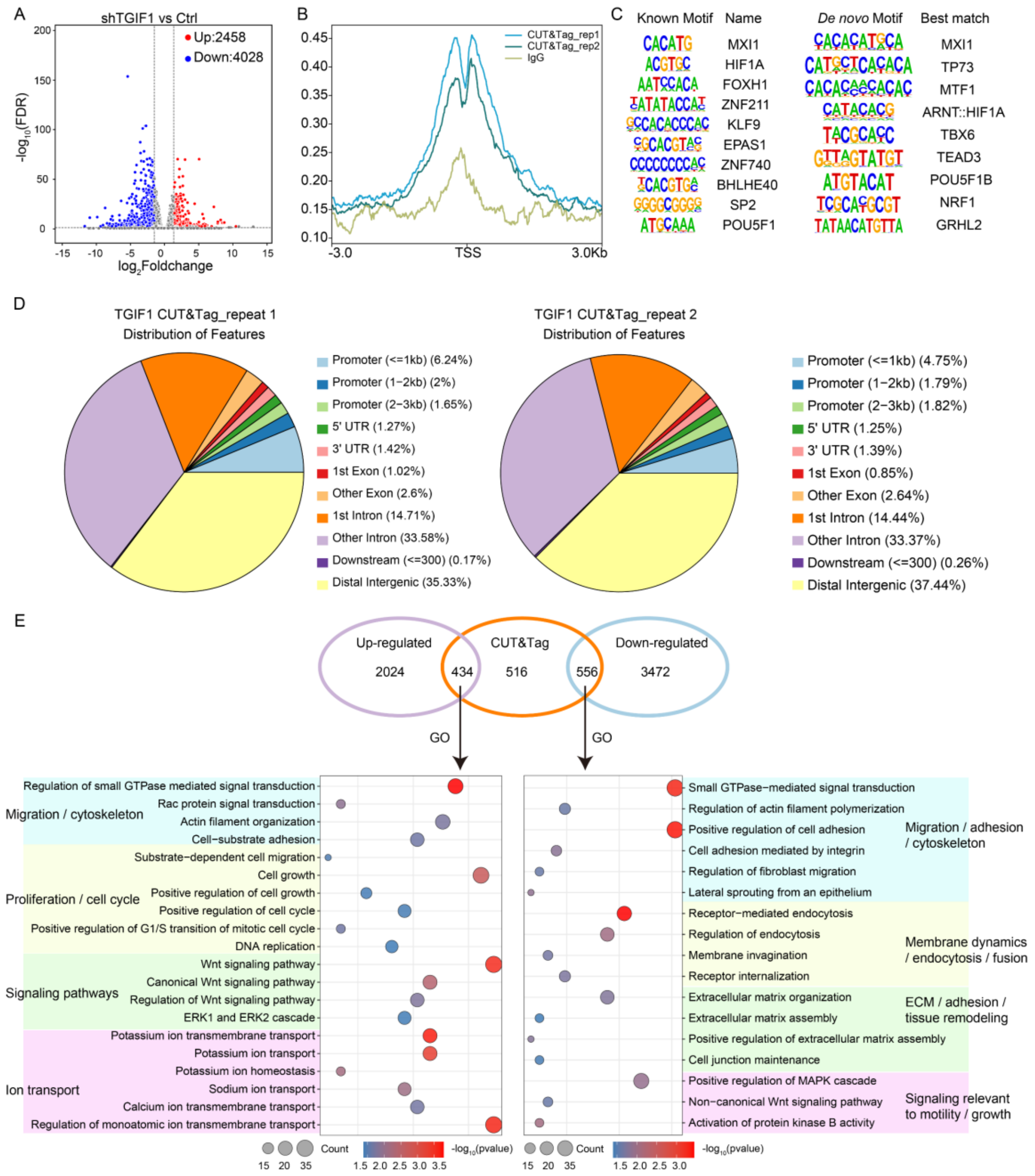

Fig.8: Genome-wide identification of the binding motifs of TGIF1. (A) The volcano plot of DEGs (sh*TGIF1* vs control). (B) Distribution of TGIF1 CUT&Tag signals across transcription start sites (TSS) (±3 kb), showing promoter enrichment. (C) Representative enriched known and *de novo* motifs identified from TGIF1 CUT&Tag peaks using HOMER. To avoid overrepresentation of highly similar motifs, representative motifs from distinct motif classes are shown. Full ranked motif enrichment results are provided in Supplementary Fig. 14D. (D) The genomic distribution profile of TGIF1 CUT&Tag peaks (repeat 1 and repeat 2). (E) The Venn diagram showing the overlap between TGIF1 peak-associated genes and the DEGs identified by RNA-seq, as well as GO analysis of the overlapping gene sets.

Fig 8G could be supplemental data, allowing more room for a more thorough analysis of the motifs and binding versus changes in expression.

Response: Following the reviewer's suggestion, we have removed Figure 8G (genome browser IGV tracks) to Supplementary Figure 14F.

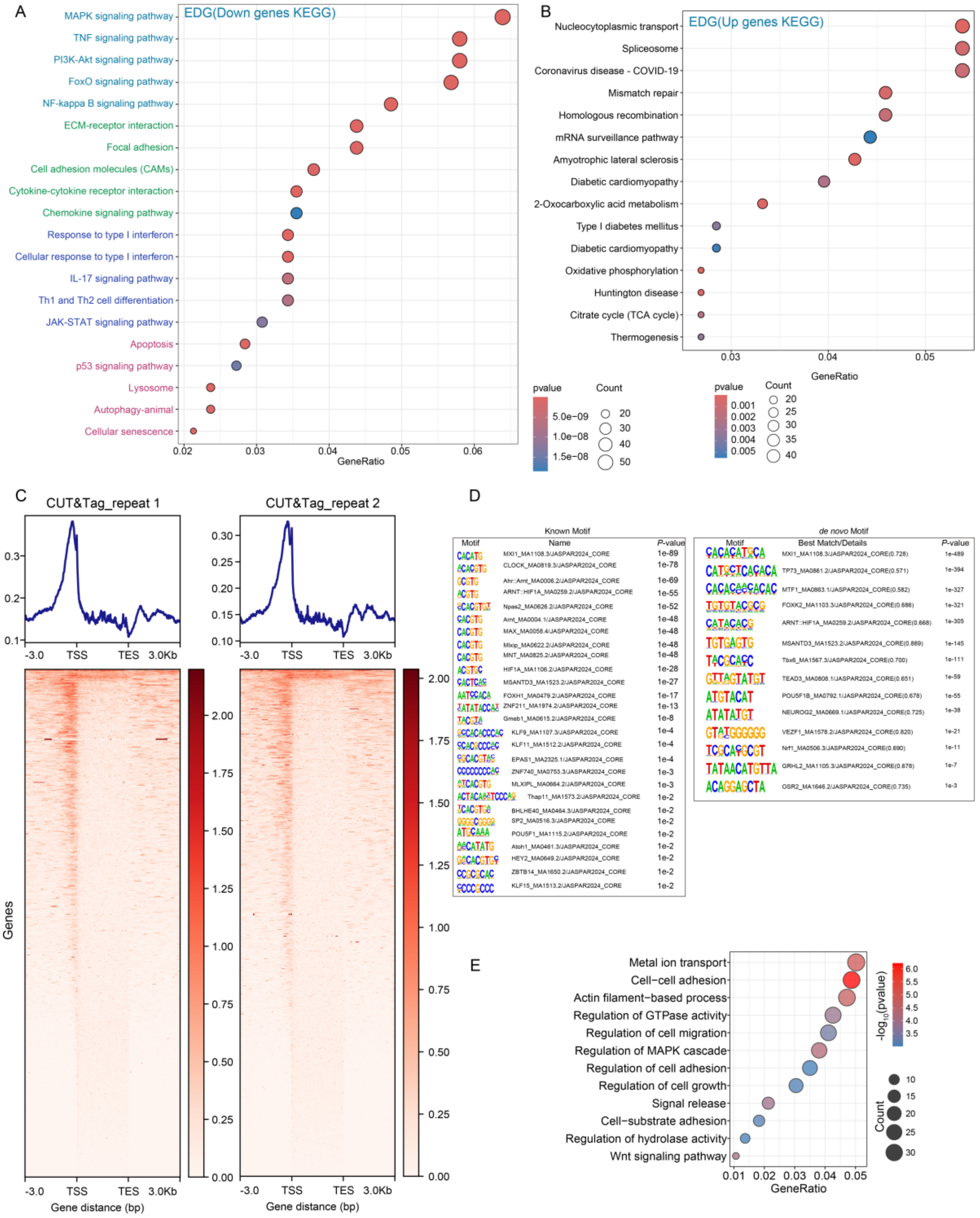

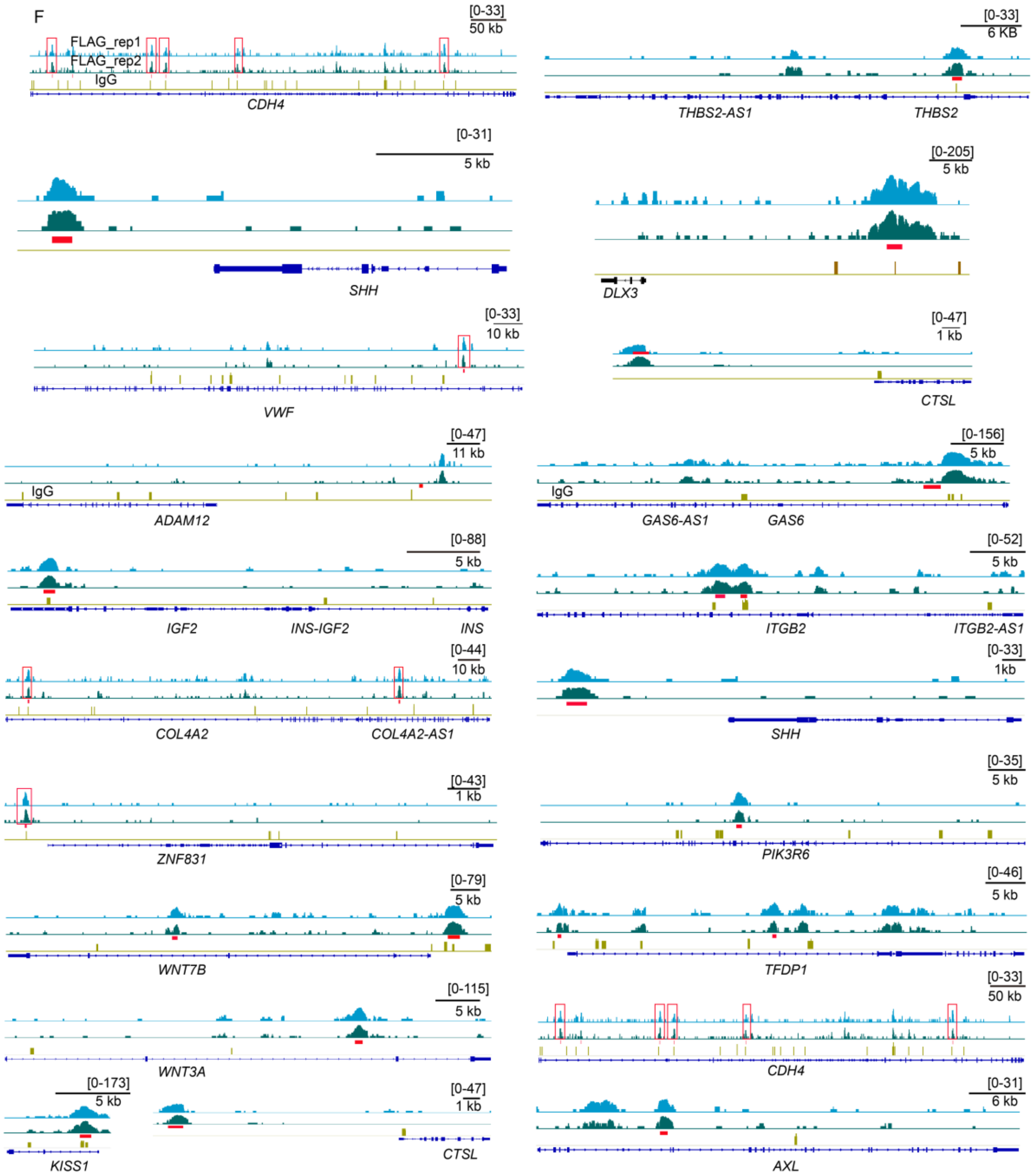

Supplementary Fig.14: Genome-wide identification of the binding motifs of TGIF1.

(A-B) KEGG enrichment analyses of downregulated (A) and upregulated (B) DEGs. The x axis indicates GeneRatio, and dot size indicates the gene count, while the color indicates the *P* value. The enrichment analysis was performed using clusterProfiler. (C) Heatmaps and average profiles of TGIF1 CUT&Tag signal intensity near transcription start sites (TSS) and transcription end sites (TES) in biological duplicates (repeat 1 and repeat 2). (D) The motif enrichment analysis of TGIF1-binding sites performed using HOMER. Known motifs were identified by comparing with reference motif databases, whereas *de novo* motifs were predicted directly from TGIF1-binding sequences and matched to the closest known motifs. *P* values indicate motif enrichment significance. (E) The GO enrichment analysis of the complete set of overlapping genes between

TGIF1 peak-associated genes and DEGs, including both upregulated and downregulated ones. The x axis indicates GeneRatio, and dot size indicates the gene count, while the color indicates $-\log_{10}$(P value). (F) Genome browser tracks showing representative TGIF1-binding peaks.

Reviewer #3 (Remarks to the Author):

This manuscript by Tan et al investigates the molecular evolution of the placenta by collecting single cell sequencing datasets from placental tissue from ten different species of mammals representing the four major placental types. The authors identify trophoblasts as harboring the most gene expression variation across species and propose that these cells are the driver of placental diversification. They then used pseudotime analysis, differential gene expression analysis, and pathway analysis to further investigate how trophoblasts contribute to the development of the different major placental types. During these analyses they note that genes and pathways involved in pre-eclampsia are specifically upregulated in human extravillous trophoblasts. The authors then explore the molecular and evolutionary patterns of pre-eclampsia associated genes across their data. Finally, they explore the function of the human gene TGIF1 in trophoblast function and pre-eclampsia. The authors find that TGIF1-binding peaks within the promoter or body regions of multiple genes closely associated with trophoblast cell proliferation, migration, invasion, and pathogenesis of pre-eclampsia and pregnancy loss.

This novel cell atlas and the resulting analyses are an asset to future studies on placental evolution, development, and pre-eclampsia. This study represents a significant body of work that will be of broad interest to a variety of fields.

The authors responded thoroughly to the comments from previous reviewers, which greatly expanded their work. I provide only the following additional comment:

Most of the datasets used were single-nucleus RNA sequencing, while three (Human, Macaca, and Rat) datasets were single-cell RNA sequencing. These two

methodologies capture different transcriptomic profiles (single-nucleus only gets nuclear transcripts, while single cell gets nuclear and cytoplasmic transcripts), and single-nucleus RNA sequencing typically captures fewer genes overall. Normalization can't fix this library complexity and capture efficiency issue, and these biases could affect differential gene expression analyses, which many of the conclusions in this paper are based on. For example, if the human single-cell dataset has more genes per cell captured and more transcripts per gene captured than the single-nucleus dataset being compared to it, then we are more likely to conclude that the human dataset has a gene present or upregulated comparatively that may actually be an artifact. Adding figures to the supplement on the number of genes captured per cell in each sample, and the average number of transcripts captured per gene in each sample, could add context to help understand what impact methodology could be having, and will generally help quantify data quality. Additionally, adding a caveat somewhere in the paper text about this potential effect on data interpretation would help readers who are not as familiar with these technologies and methods better evaluate the conclusions presented.

Response: We thank the reviewer for this valuable comment. We agree that single-nucleus RNA sequencing (snRNA-seq) and single-cell RNA sequencing (scRNA-seq) have inherent differences in transcript capture range and library complexity, and that these technical differences may affect the interpretation of gene detection rates and expression levels in cross-species and cross-dataset comparisons. In response to the reviewer's suggestion, we have added supplementary data summarizing quality control- and capture-related metrics for each sample, including (1) the number of genes detected per cell/nucleus in each sample and (2) the average number of transcripts captured per gene in each sample (Supplementary Data 1). These results provide readers with more direct context for evaluation of potential differences in capture efficiency and library complexity among datasets generated using different sequencing strategies, and help assess their possible impact on downstream analyses.

In addition, we have added a corresponding caveat to the Discussion (Lines 942-947), as follows:

“Nevertheless, a limitation of this study is that the integrated datasets were generated using both snRNA-seq and scRNA-seq that differ in transcript capture range, library complexity, and gene detection sensitivity. Although normalization and integration were performed to reduce technical bias, these differences may still affect the cross-species comparisons and the differential expression analyses. The corresponding results should hence be interpreted with appropriate caution.”
